# Phenotypic Diversity in GNAO1 Patients: A Comprehensive Overview of Variants and Phenotypes

**DOI:** 10.1155/2023/6628283

**Published:** 2023-08-07

**Authors:** Maria Sáez González, Kes Kloosterhuis, Laura van de Pol, Frank Baas, Harald Mikkers

**Affiliations:** ^1^Department of Clinical Genetics, Leiden University Medical Centrum, Leiden, Netherlands; ^2^Department of Cell & Chemical Biology, Leiden University Medical Centrum, Leiden, Netherlands; ^3^Department of Child Neurology, Emma Children's Hospital, Amsterdam UMC Location University of Amsterdam, Amsterdam, Netherlands; ^4^Department of Child Neurology, Amsterdam UMC, Location Vrije Universiteit Amsterdam, Amsterdam, Netherlands

## Abstract

GNAO1 disorder is a rare autosomal dominant neurodevelopmental syndrome that is clinically manifested by developmental delay, (early onset) epilepsy, and movement disorders. Clinical symptoms appear very heterogeneous in nature and severity, as well as the response of GNAO1 patients to available medication varies. Pathogenic *GNAO1* variants have been found mainly scattered throughout the gene although certain mutation hotspots affecting the function of the encoded G*α*o proteins exist. *GNAO1* variants only partially explain the diverse phenotypic spectrum observed but full stratification has been hampered by the limited number of patients. The aim of this review was to generate a comprehensive overview of the germline variants in *GNAO1* and provide insight into the phenotypic diversity of the GNAO1 disorder. We compiled a list of 398 *GNAO1* germline variants. In addition, we provide the *GNAO1* variants and associated phenotypes of 282 GNAO1 patients reported in case reports, whole genome sequencing studies, genetic variant databases, and 8 novel GNAO1 patients that were not described before. This has resulted in a list of 107 (likely) pathogenic *GNAO1* variants. Available phenotypic data was utilized to quantitatively assess the genetic and phenotypic diversity of the GNAO1 disorder and discuss the outcomes. This inventory forms the basis for a *GNAO1* variant database that will be updated continuously. Moreover, it will aid genetic diagnostics, medical decision-making, prognostication, and research on the mechanisms underlying the GNAO1 disorder.

## 1. Introduction

In 2013, Nakamura and colleagues discovered that heterozygous *de novo* mutations in *GNAO1,* a gene encoding the 354 amino acid- (AA-) sized guanine nucleotide-binding protein subunit alpha (other), cause a rare disorder hallmarked by early-onset epileptic encephalopathy, developmental delay, and movement disorder (MD) [1]. Consequently, *GNAO1* was named early infantile epileptic encephalopathy gene 17 (*EIEE17).* Propelled by advances in whole genome sequencing diagnostics, many additional *GNAO1* variants have been found since. Unfortunately, data on *GNAO1* variants and accompanying clinical phenotypes are scattered over multiple sources, which are not easily accessible and often incomplete. The lack of a full overview of variants and accompanying phenotypes in rare disorders like the GNAO1 disorder, where the number of patients is very limited and the phenotype is heterogeneous, has hindered diagnostics, treatment decision-making, prognostication, and research into the underlying pathogenic mechanisms. To create a comprehensive overview of all germline *GNAO1* variants, we have gathered all publicly available *GNAO1* variant data from various genome variation databases including ClinVar, Decipher, dbSNP55, gnomAD, Leiden Open Variation Database (LOVD), and VarSome. In addition, we describe variant data and phenotypes from *GNAO1* patients collected from case studies, whole genome sequencing reports, and through physicians in the Netherlands as well as parents of GNAO1 patients with the help of the Dutch (GNAO1.nl) and Spanish (GNAO1.es) GNAO1 patient organizations with full consent from the local ethical boards and legal guardians.

A total of 398 germline *GNAO1* missense and nonsense mutations, indels, and variants predicted to affect the splicing of *GNAO1* are present in the various genomic databases and reported GNAO1 patients (Supporting Information: Table S1). All variants were deposited into the LOVD database (databases.lovd.nl/shared/genes/GNAO1). A large majority of *GNAO1* variants were predicted to have a deleterious effect when analysed using prediction algorithms like Polyphen2-HVID and VEST4, and the highly performing metapredictors BayesDel-AFF, ClinPred and REVEL [2, 3]. *GNAO1* variants were collated on basis of the American College of Medical Genetics and Genomics (ACMG) classification of pathogenicity [4]. If access to additional experimental evidence and/or inheritance patterns of variants provided new insights into the classification, we reevaluated the classification and changed these according to current ACMG guidelines. All (likely) pathogenic variants are summarized in Supporting Information, Table S2. To further enable genotype and phenotype enumerations and stratifications, we also established a comprehensive overview of genotypes and concomitant neurodevelopmental phenotypes of 282 patients with a (likely) pathogenic *GNAO1* variant or a variant of uncertain significance (VUS) (Supporting Information: Table S3).

## 2. *GNAO1* and the Encoded G*α*o Proteins

**Figure 1:**
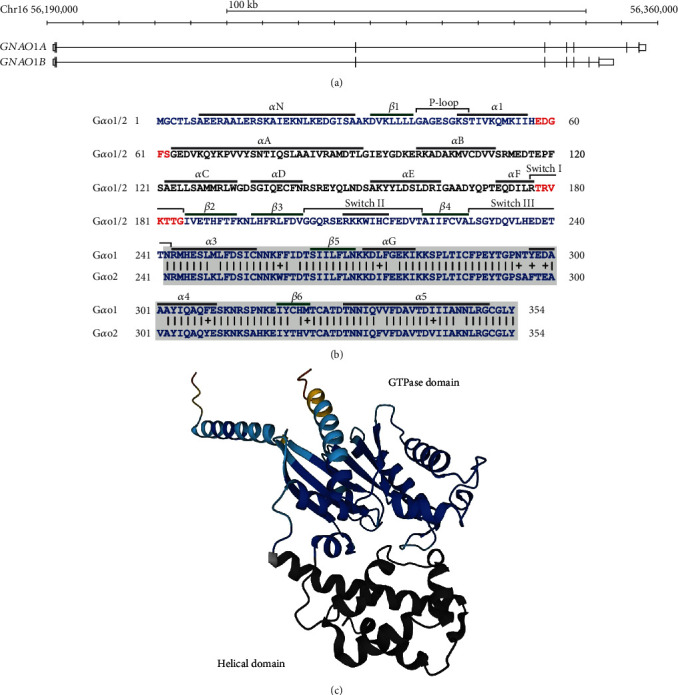
Structure of *GNAO1* and the encoded proteins. (a) Overview of the *GNAO1* locus (chr16: 56,190,000-56,360,000 hg38) containing *GNAO1A* and *GNAO1B*. (b) Alignment of the *GNAO1* encoded human G*α*o1 and G*α*o2 protein sequences. The protein sequence before the start of the alignment (AA242) is shared by G*α*o1 and G*α*o2. AAs in the helical domain are shown in black, the linker region in red, and the GTPase region in blue. Indicated are the alpha helices and beta strands that were determined on the basis of the AlphaFold structure (A0A3Q1MSI3) and other important regions, whose positions were derived from Johnston et al. [8]. (c) Predicted structure of bovine G*α*o1 by AlphaFold (A0A3Q1MSI3). The helical domain is dark gray, and the GTPase part is depicted in the colours for the per-residue confidence score (pLDDT) given by Alphafold. Blue (very high: pLDDT > 90), cyan (high: 90 > pLDDT > 70) and yellow (low: 70 > pLDDT > 50).

**Figure 2:**
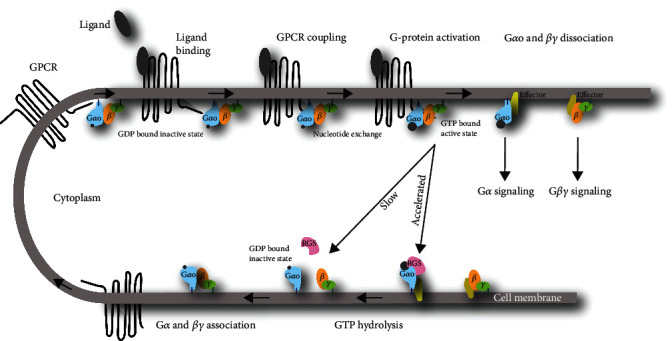
Classical view of G*α*o signaling. Overview of the different stages of the G*α*o activation cycle and the canonical signaling of GPCRs through G*α*o. Of note and not depicted: G*α*o likely performs also GPCR-independent functions like KEDLR-mediated activation of RAB4 that is independent of G*βγ* [57].

Transcription of the *GNAO1* locus generates the *GNAO1A* (NM_020988.3) and *GNAO1B* (NM_138736.3) isoforms through alternative splicing. The isoforms share the first 6 exons but have distinct isoform-specific 3′ coding exons (Figure 1(a)). Translation of *GNAO1A* (NM_020988.3) and *GNAO1B* (NM_138736.3) yield very similar proteins, namely, G*α*o1 and G*α*o2, respectively. Both proteins are 354AA and diverge only in 20 AAs, which are found scattered over the variant carboxy-terminal region from AA242 through AA354 (Figure 1(b)). G*α*o proteins are catalytically active G alpha subunits that associate with G*βγ* heterodimers to form heterotrimeric G proteins, which transduce signals from the activated G protein-coupled receptor (GPCR) into the cell. More than 200 GPCRs, including acetylcholine, dopamine, GABA, and opioid receptors, activate G*α*o [5]. G*α*o proteins are located at the inner membrane close to the GPCRs by virtue of G-myristylation and S-palmitoylation of residues Gly2 and Ser3 [6, 7]. The structure of G*α*o consists of one GTPase and one helical domain, which are connected by two linkers (Figure 1(c)). The GTPase domain consists of 6 helices that envelope 6 *β*-sheets (*β*1-*β*6), while the helical domain contains 6 helices (*α*A–*α*F). The G*α*o heterotrimer binds GDP in the inactive form; upon ligand binding by an adjacent GPCR, the G protein associates with the GPCR, and GDP is exchanged for the more abundant GTP (classical view) (Figure 2). Alternatively, the GDP-bound G protein is already associated to an inactive GPCR, and upon ligand binding, the GPCR activates the bound G protein (prebound view). Substitution of GDP with GTP results in the dissociation of the heterotrimer into the G*α*o subunit and a G*βγ* dimer that each modulates specific signalling routes through downstream subunit-specific effector molecules. Eventually, G*α*o inactivates itself by intrinsic hydrolysis capacity, which can be accelerated by GTPase activating proteins (GAPs) that contain a regulator of G protein signalling (RGS) motif.

Exchange of GDP for GTP, dissociation of the heterotrimer, and GTP hydrolysis reverting the protein to a GDP-bound state are meticulously orchestrated by consecutive allosterical changes coordinated by the three switch regions, switch I (AA177-184), switch II (AA203-220), and switch III (AA 228-242) [8]. Switch sections are flexible regions that create important interfaces for the binding of GDP/GTP, Mg^2+^, and G*βγ* but also effector molecules, guanosine dissociation inhibitors (GDI), GTPase-accelerating proteins, and chaperones [8]. Besides the switch regions, G*α*o proteins contain other regions, such as a P-loop (AA40-47), providing additional anchor points for guanine nucleotides and Mg^2+^ [9], GPCRs [10], chaperones [11], and RGS proteins [12].

## 3. Which *GNAO1* Isoform Is Responsible for the GNAO1 Disorder?

An unresolved point of debate has been whether only mutations in *GNAO1A* cause a neurodevelopmental disorder (NDD) or whether *GNAO1B* variants also provoke NDD. Both *GNAO1* isoforms are ubiquitously transcribed in the brain and encode very similar protein products. Therefore, genetic variants in either isoform could hypothetically cause the GNAO1 disorder. Recently, it was proposed that *GNAO1A* is the main pathogenic isoform as p.E246K variants are only observed in the *GNAO1A* isoform in patients with an NDD phenotype [13]. However, the higher prevalence of *GNAO1A* mutations could be merely explained by differences in the mutation load due to isoform-specific divergent genomic sequences or different functions of the G*α*o isoforms, which would lead to different clinical phenotypes when mutated.

Evaluation of all pathogenic and likely pathogenic *GNAO1* variants in exons 7 and 8 that allow for discrimination between the two *GNAO1* isoforms (Figure 1(a)) should elucidate which variant causes an NDD phenotype. We identified 90 patients with a variant in the last 2 exons of either *GNAO1* variant in the collated database and from unique variant submissions in ClinVar. *GNAO1A* variants are much more frequently observed in these 90 NDD patients than variants in *GNAO1B*. Six out of 90 isoform-specific variants are in *GNAO1B.* This underrepresentation of *GNAO1B* variants indicates that mutations in *GNAO1A* are likely the predominant cause of the GNAO1 disorder. To further investigate this phenomenon, we examined the *GNAO1B* variants found in patients with a NDD phenotype. One of the *GNAO1B* variants, *c*.770*A* > *G*; p.N257S, is classified as benign, whereas *c*.1045*C* > *T*; p.R349W is predicted to be possibly damaging and classified as a variant of uncertain significance (VUS) in ClinVar. Since *c*.1045*C* > *T* is not *de novo* in a Dutch patient and inherited from a nonmosaic father without a phenotype, we propose to reclassify this variant as likely benign. The other four *GNAO1B* variants, namely, *c*.818*A* > *T*; p.D273V, *c*.856*A* > *G*; p.I286V, *c*.901*G* > *C*; p.V301L, and *c*.877 + 5*A* > *G* are classified as VUS. Of these variants, solely *c*.818*A* > *T* (p.D273V) is predicted to be deleterious. Interestingly, an identical variant was also found in *GNAO1A* of a patient with a GNAO1 neurodevelopmental phenotype [14], indicating a potential contribution of *GNAO1B* to the development of NDD. One explanation for the lower frequency of *GNAO1B* variants in patients with a neurodevelopmental phenotype could be a lower frequency of germline variants in *GNAO1B*, caused by either larger deleterious effects of variants that are incompatible with life or lower mutation loads due to isoform-specific DNA sequences or chromatin structures. However, the presence of 132 *GNAO1B-*specific germline and only 70 *GNAO1A*-specific variants in the genome variant databases strongly argues against either hypothesis (Fisher exact test *p* value < .00001) and implies the existence of increased tolerance for and reduced pathogenicity of *GNAO1B* variants during development in addition to the absence of lower mutation rates in the *GNAO1B*. This view is further supported by the presence of the *c*.736*G* > *A* (p.E246K) variant (rs775322429) in *GNAO1B*, which is identical to the highly pathogenic *c*.736*G* > *A* (p.E246K) mutation in *GNAO1A,* in other population cohorts such as gnomAD and TOPMed (Supporting Information: Table S1).

Taken together, available *GNAO1* variant data indicate that *GNAO1A* prevails as the predominant pathogenic isoform. However, rare *GNAO1B* variants, like *c*.818*A* > *T* (p.D273V), should not be neglected as NDD-causal variants at this stage. *GNAO1B* variants may be less pathogenic, yielding more subtle phenotypes, which may appear only later in life as has been reported for specific *GNAO1A* missense variants or deletions [15, 16]. To formally exclude a causal role of specific *GNAO1B* variants, more experimental and functional evidence is required.

## 4. Distribution of Pathogenic Variants in G*α*o1

**Figure 3:**
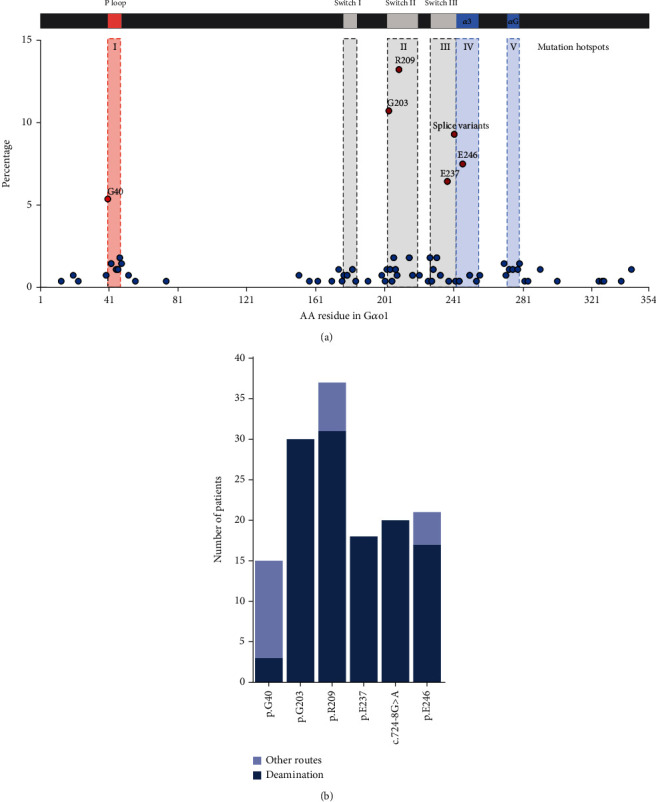
Enumeration of pathogenic and likely pathogenic *GNAO1A* germline variants. (a) Distribution of (likely) pathogenic *GNAO1* variants across G*α*o1 that have been found in 282 GNAO1 patients. Variants are either (likely) pathogenic variants or variants provoking a typical GNAO1 phenotype. If additional, indisputably unique, patients (submissions) were found in ClinVar these were included as well. Indicated functional G*α*o domains were derived from [8]. Mutation hotspots are indicated by dotted lines. (b) Number and frequency of *GNAO1A* variants in mutation hotspots that are caused by either CpG deamination (red bar) or other mutation routes (gray bar).

Analysis of the genetic variants underlying the GNAO1 disorder has been rather rudimentary due to the limited number of patients included. Mutation hotspots have been confined to the P-Loop, switch II, and switch III regions [14], but a larger dataset is expected to provide a more in-depth overview of the distribution of pathogenic variants and may reveal additional *GNAO1A* regions that are frequently mutated in NDD patients. To establish the distribution of *GNAO1A* variants across G*α*o1, we collated all NDD patients with (likely) pathogenic *GNAO1A* variants, patients with variants provoking a typical GNAO1 disorder phenotype, and patients with *GNAO1A* variants submitted to ClinVar that are indisputably unique. The distribution of the variants observed in 282 patients across G*α*o1 revealed a desert of pathogenic variants in the helical domain ranging from AA77-151. Since 62 variants in this region have been identified in the germline of different population cohorts (Supporting Information: Table S1), it seems unlikely that variation within the helical domain confers a severe phenotype. Our analysis revealed five mutation hotspots, all of which locate to the GTPase domain (Figure 3(a)). Most of the mutations are in previously recognized mutation hotspots, which affect G*α*o1 regions that are critical for guanine binding and G*βγ* dissociation, like the P-loop (10.7%; hotspot I), switch II (31.4%; hotspot II) and switch III (20.4%; hotspot III) domains [8], and in the *α*3 helix (8.9%; hotspot IV) that is connected to the switch III region. In addition to these hotspots, the *α*G helix that ranges from AA272 to 279 is frequently mutated (4.6%; hotspot V). Analysis of each mutation hotspot reveals that certain AAs are much more frequently mutated in hotspot regions I through IV. Half of the P-loop variants concern p.G40 (p.G40E/R/W) substitutions, whereas p.G203 (p.G203R) and p.R209 (p.R209C/G/H/L/P) are predominantly mutated in the switch II region (34.5% and 42.5%, respectively). Mutations in switch III concern mainly p.E237K (32.1%) or a splice variant (*c*.724 − 8*G* > *A*) (46.4%) that creates a new splice acceptor leading to the insertion of a proline and glutamine between AA241 and AA242 [17], whereas most of the hotspot IV variants affect p.E246.

As expected, every frequent single AA substitution contains a CpG-containing codon. CpG sequences have a 10-fold higher mutation rate and account for one-third of all single nucleotide variants causing a genetic disorder [18]. CpG mutations are the consequence of 5′-methylcytosine deamination, a process in which the cytosine is converted into uracil. Methylated CpGs are more frequently mutated. This fits with the observation that most CpG mutations, except for *c*.625*C* > *T* (p.R209C), are caused by CpG deamination of the antisense strand. Interestingly, only 20.0% of the G40 mutations are the result of CpG deamination, whereas CpG deamination is the chief origin of the other frequent variants (Figure 3(b)). In contrast to the sequence encoding the P-loop region, the switch II, switch III, and the *α*3 helix contain additional codons with a CpG motif that could lead to missense variants, for example, p.R206, p.E239, p.T241, and p.D252. However, germline variants affecting these AAs are either never found (p.E239K), only found in the healthy population (p.T241M), or less frequently observed in NDD patients (p.R206E; p.D252N). The reduced frequency of p.R206E may be directly related to the caused pathogenic effect regarding severity and age of onset. For example, patients with p.R206Q manifest abnormalities at the age of 15 years or beyond [16], whereas p.G203R patients develop symptoms during the first couple of months after birth.

A pathogenic impact of p.G40, p.G203, p.R209, p.E237, and p.E246 variants is expected from the known function of the AAs and the regions in which they reside. The P-loop plays an important role in guanine nucleotide binding and is required for instigating an active conformation that is mediated through loss of the interaction with switch I and establishing a new interaction with the *α*3 helix [19, 20]. After this switch, the *γ*-phosphate of GTP is secured by the G204-R209-E246 triad that governs the dissociation of G*βγ* [20]. Upon binding of GTP, G204 enables the formation of a salt bridge between R209 and E246 that locks G*α*o in an active conformation [20, 21]. In addition, G204 forms a direct polar interaction with E237 in switch III [20]. Thus, substitutions within or close to this G204-R209-E246 triad hinder the most likely formation of a polar network between the *γ*-phosphate of GTP and the G*βγ*-binding interphase, reducing G*βγ*-dissociation.

Besides missense mutations, nonsense variants are sporadically observed in patients with a NDD phenotype, such as *c*.529*C* > *T* p.R177^∗^ (ClinVar), *c*.616*C* > *T* p.R206^∗^ [22], and c.765dupT; p.N256^∗^ [16]. Furthermore, *GNAO1A* variants that likely generate a truncated out-of-frame protein, either caused by a deletion such as c.676_677del p.V226Rfs^∗^6 (ClinVar) or by single nucleotide variants that affect splicing including *c*.723 + 1*G* > *A* [23], *c*.723 + 1*G* > *T* [24] and *c*.723 + 2 *T* > *A* (this report) are observed in NDD patients. Splice variants that lead to a frameshift have been classified as (likely) pathogenic, whereas nonsense variants have been classified from likely benign to likely pathogenic. However, in view of a recent study postulating the haploinsufficiency of *GNAO1* [15], all variants leading to a premature translation-termination leading to nonsense-mediated mRNA decay (NMD) are likely pathogenic. *GNAO1* haploinsufficiency is often manifested as a very mild movement disorder with relatively late onset, which may explain the lack of an obvious linkage of nonsense variants to the development of NDD.

## 5. Phenotypes of the GNAO1 Disorder

The main GNAO1-associated phenotypes are epilepsy, movement disorder, and global developmental delay. A report on 46 GNAO1 patients indicated a large degree of phenotypic heterogeneity between GNAO1 patients [14]. Although a correlation between phenotypes and variants provoking a loss-of-function or gain-of-function/normal function was postulated [25], a mutually exclusive correlation between phenotype and genotype has never been found. To further investigate the heterogeneity, the collected phenotypic data of the 282 patients with a potentially pathogenic *GNAO1A* variant (Supporting Information: Table S3), including 8 patients who have not been described earlier (Table 1), were used.

### 5.1. Epilepsy

**Figure 4:**
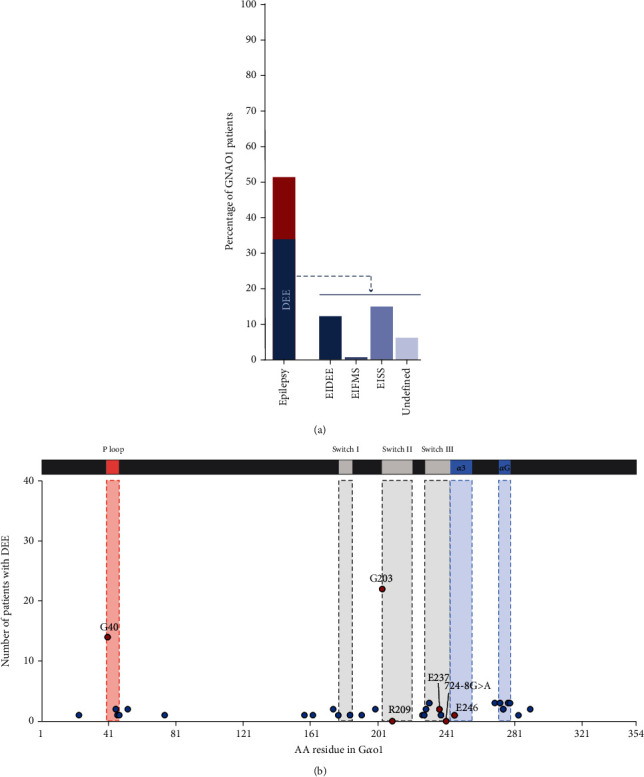
Enumeration of epilepsy in GNAO1 patients. Frequency at which an epileptic phenotype is observed in GNAO1 patients (*n* = 232) and distribution of the type of DEE. The type of DEE was deduced from the available EEG description and age of onset. Unfortunately, a detailed description of DEE subtypes and EEG recordings was not always available. Furthermore, overlapping symptoms between subtypes make it more difficult to correctly group DEE subtypes. (b) Number of patients with DEE (*n* = 50) caused by missense mutations or indels. Percentages of GNAO1 patients with DEE are indicated for AA variants that have been found in >5 DEE patients. Location of the P-loop and switch I, II, and III regions is indicated. Infantile epileptic spasms syndrome (IESS), early infantile DEE (EIDEE), epilepsy of infancy with migrating focal seizures (EIMFS).

Based on the available phenotypic data of patients with *GNAO1A* variants that are classified as (likely) pathogenic or VUS, 53.0% of the GNAO1 patients develop epileptic seizures at some point during their life (Figure 4(a)). Two-thirds of which suffer from EIEE, which is nowadays referred to as developmental and epileptic encephalopathy (DEE). DEE is hallmarked by an early onset (<1 year of age) of epilepsy, abnormal neurological findings such as unusual posture, muscle tone, or movement in combination with a developmental delay. The clinical profile of DEE is very heterogeneous and contains many different epilepsy subtypes, which were recently grouped into early infantile DEE (EIDEE), epilepsy of infancy with migrating focal seizures (EIMFS), infantile epileptic spasms syndrome (IESS), and Dravet's syndrome [26]. Most of the GNAO1-DEE patients present either EIDEE, which includes the Ohtahara syndrome that was originally linked to the GNAO1 disorder [1] and is hallmarked by a burst suppression pattern on an electroencephalogram (EEG) and tonic seizures, or IESS that is also known as West syndrome with hypsarrythmia on EGG (Figure 4(a)). EIFMS has been reported in only 1 patient [27]. In addition, almost 20% of the DEE could not be classified into one DEE subgroup on the basis of the available phenotype description and EEG data. GNAO1 DEE hotspots are found at G40 in the P-region, at G203 in the switch II region, and in the regions containing AA227-231 and AA270-279 (Figure 4(b)).

Besides DEE, over 30.0% of the GNAO1 patients develop epilepsy at a later stage during childhood with an average onset of 5 years and 3 months (Supporting Information: Table S3). However, this percentage is likely an underestimation because phenotype descriptions are not or only sporadically updated after the publication of the case report or after the initial submission into genome variant databases.

### 5.2. Movement Disorder

**Figure 5:**
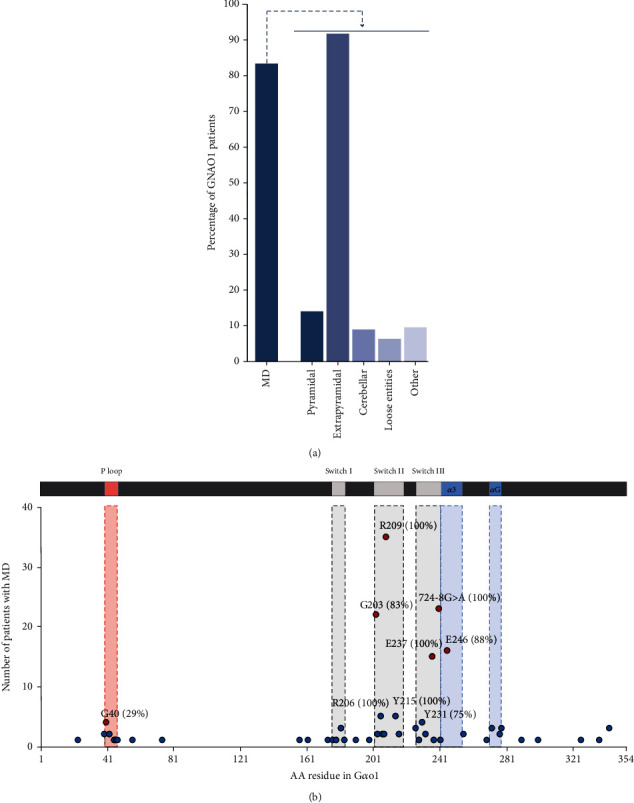
Evaluation of MD in GNAO1 patients. (a) Frequency at which an MD phenotype is observed in GNAO1 patients (*n* = 223) and distribution of the type of MD symptoms (*n* = 157 MD patients). A single patient may exhibit multiple MD symptoms. Frequencies of MD subtypes are calculated from 157 GNAO1 MD patients, for which a more detailed description was available. (b) Number of patients with MD caused by missense mutations or variants affecting splicing which result in AA insertions or truncated out-of-frame products. Percentages of GNAO1 patients with MD are indicated for AA variants that have been found in >4 MD patients. Location of the P-loop and switch I, II, and III regions is indicated. Mutation hotspots are depicted as red circles. Of note, depicted percentages and absolute numbers are only an indication as movement symptoms may change over time or may arise only later in childhood after the publication of the initial case report.

MD have been observed in 81.7% of the GNAO1 patients (*n* = 202) (Figure 5(a)). The average onset of a hyperkinetic phenotype is four years and ten months (Supporting Information: Table S3). MD may appear any time between the first few months after birth and the age of 47 years, which is currently the most delayed onset [16]. This may be dependent on the *GNAO1* variant. Consequently, *GNAO1* is not only in the diagnostic panel for epilepsy but also in the standard gene panel for diagnostic screening of MD in most countries. Clinicians have described a wide variety of movement symptoms in GNAO1 patients including akathisia, ataxia, choreoathetosis, ballism, bradykinesia, chorea, dystonia, dyskinesia, myoclonus, parkinsonism stereotypies, repetitive movements, spasms, and spasticity. To simplify enumeration of the often largely diverse MD descriptions, on which treating neurologists may not always reach consensus [28], we categorised the movement symptoms into the following groups: cerebellar (ataxia), pyramidal (spasms and spasticity), extrapyramidal (ballismus, chorea, choreoathetosis, dyskinesia, dystonia, and parkinsonism), loose entities (akathisia and myoclonus), and other (stereotypies and repetitive movements)(Figure 5(a)). However, patients may display multiple MD symptoms fitting into one or multiple subgroups. Furthermore, MD symptoms in GNAO1 patients often progress over time into severe exacerbations. Since phenotype descriptions are not or only sporadically updated after the initial case report or submission into genome variant databases, only a full natural history of the disorder allows for a complete categorisation.

Mutation hotspots of variants causing MD are in the switch II and in or near the switch III regions of G*α*o1 (Figure 5(b)). p.G203R and p.R209-substitutions are the most frequently found variants in switch II. p.E237K, splice variants that result in a GluPro insertion at AA242, and p.E246K are the most frequent variants in the switch III or the adjacent *α*3 helix (Figure 5(b)). Missense mutations and indel variants in the switch III/ *α*3 helix cause almost exclusively an MD phenotype. In line with this, the *c*.723 + 1*G* > *A*, *c*.723 + 1*G* > *T*, and *c*.723 + 2 *T* > *A* variants, which destroy the splice donor site of exon 4 resulting into a frameshifted and early terminated G*α*o1, confer exclusively an MD phenotype.

### 5.3. Mixed Epilepsy-Movement Disorder

**Figure 6:**
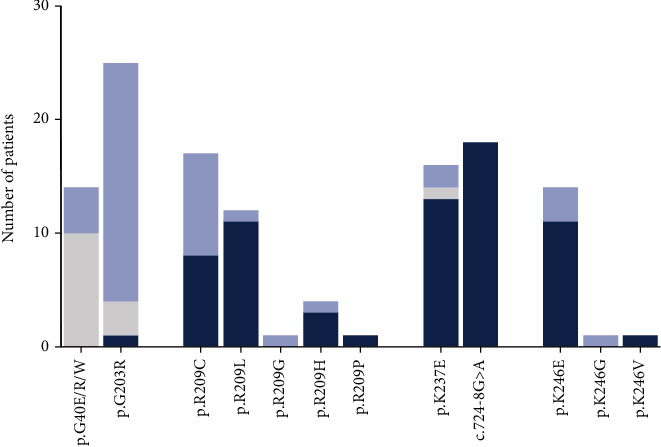
Evaluation of phenotypes in patients with a mutation hotspot variant. Absolute number of patients with a certain phenotype. Epilepsy = gray bar; MD = dark blue bar and mix of epilepsy; and MD = light blue bar.

Evaluation of all collected phenotype data demonstrates that a great majority of the GNAO1 variants give a mixed epileptic-MD phenotype (Supporting Information: Table S3). Pure epileptic, MD, or mixed phenotypes appear to occur more often in patients with certain variants; however, as the phenotypic dataset does not include a full natural history of most patients, a later onset of MD or epilepsy may have been missed, because the MD or epilepsy was not present at the time of publication or the patient succumbed before an additional phenotype had developed. Nevertheless, patients with an identical *GNAO1A* variant may display distinctive symptoms. Analysis of the phenotypes in patients with an AA mutated in one of the hotspots underscores phenotypic heterogeneity in GNAO1 patients (Figure 6). p.G40R/W/E variants always cause DEE (*n* = 14), while one-third of the p.G40 patients, p.G40R or p.G40E, develop a form of movement disorder (*n* = 12). p.G203R yields a very severe but mixed epilepsy-MD phenotype. Most of the patients develop DEE (*n* = 22 out of 25) and some epilepsy during childhood (*n* = 2 out of 19), whereas 1 patient had not yet developed epilepsy at the age of 46 months. DEE subtypes observed in p.G203R patients include IESS, IEDEE, and IEFMS. In addition, the large majority of p.G203R patients (*n* = 22 out of 25) suffer from a severe form of movement disorder. Patients with a p.R209 germline variant invariably develop uncontrolled hyperkinetic symptoms. A proportion of the patients with p.R209C or p.R209P (*n* = 10 out of 18) develop childhood-onset epilepsy as well. The development of epilepsy may be a direct consequence of the functional severity of the mutation, as p.R209C has the largest and p.R209H the smallest effect (*p*.*R*209*C* > >*p*.*R*209*G*/*L*/*P* > *p*.*R*209*H*) on the response to pheromones in yeast [20]. MD is also the main manifestation in p.E237K, p.E246K, and *c*.724 − 8*G* > *A* patients, albeit that p.E237K (*n* = 2 out of 15) and p.E246K (*n* = 6 out of 18) patients may encounter epileptic seizures.

The onset of DEE and hyperkinetic MD varies between patients with the same AA variant as well. p.G40 substitutions cause DEE with an average onset of 47 days (*n* = 9), but the onset ranges from immediately after birth to 5 months. The MD phenotypes in p.G40 patients vary largely. Unfortunately, the age of MD onset has only been provided for 1 patient. Patients with the p.G203R variant exhibit the first signs of DEE ranging from day 0 to day 92 (*n* = 12, average onset = 21 days) and develop severe forms of MD, often starting with mild dyskinesia, within the first 2 years after birth (*n* = 8, average onset = 15 months). The onset of movement symptoms in p.R209 patients does not differ between patients with p.R209C and p.R209G/H/L/P variants. The average onset is 33 months, but the onset spans from 2 months to the age of 11 years. The first appearance of movement disorder is very similar to the other MD hotspot variants, albeit that *c*.724 − 8*G* > *A* patients appear to develop hyperkinesia a little later (average 5 years and 7 months spreading from 2 to 11 years).

### 5.4. Other Common GNAO1 Disorder Symptoms

Axial hypotonia was previously reported to be the most common symptom and to be present in >90.0% of the GNAO1 patients [14]. Axial hypotonia is often observed in infancy before other clinical symptoms appear [16, 29]. In the collected GNAO1 dataset, axial hypotonia is only described in 71.0% of the patients (Suppl. Table 3), which is likely an underrepresentation because many case studies only describe the more severe symptoms and do not explicitly state the absence of hypotonia.

**Figure 7:**
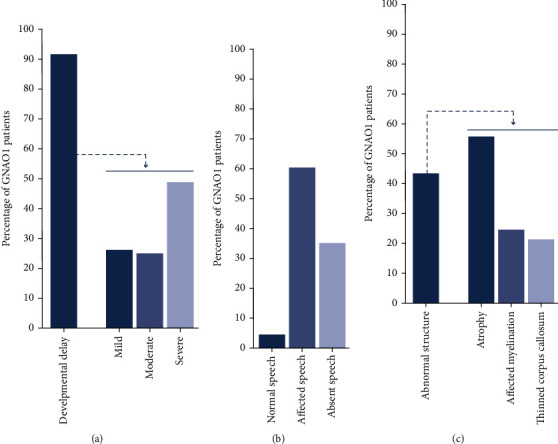
Prevalence and heterogeneity of other clinical manifestations in GNAO1 patients. (a) Frequency of developmental delay in GNAO1 patients (*n* = 204) (left), with the distribution of mild, moderate, and severe gradations (right) (*n* = 84 patients). (b) Frequency of speech problems in GNAO1 patients (*n* = 111). c) Structural changes in the brains of GNAO1 patients (*n* = 145). This number is likely an underrepresentation as MRI data from investigations at a later age are often not publicly available.

Developmental delay is another major symptom that is presented at an early stage in GNAO1 patients (86.5%) (Suppl. Table 3). Most of the patients suffer from global developmental delay, which at a later stage is separated into motor and/or cognitive disability. Available descriptions range from developmental delay, global developmental delay, motor developmental delay, and intellectual disability. A thorough classification of the patients regarding their developmental status is difficult without a detailed natural history. Because the degree of variability in delay is enormous and ranges from absent to a very severe delay (Figure 7(a)), accessibility to extensive milestone achievements during the first 5-10 years is required. In addition, comparative grading of cognitive development in GNAO1 patients is not straightforward, and quantitative cognitive tests are only occasionally reported. Without these, it is difficult to objectively determine cognition as this may be compromised by delayed development of speech or apraxia of speech (AOS) that is very commonly (>90.0%) seen in GNAO1 patients (Figure 7(b)). However, impaired speech is not always linked to intellectual disability as some GNAO1 patients with AOS have a normal fluid intelligence score [30] and can communicate through devices.

Other clinical manifestations in GNAO1 patients are dysphagia, gastrointestinal problems, microcephaly, respiratory problems, scoliosis, self-injurious behaviour, and sialorrhea. Most of these symptoms occur secondary to the physical disabilities in these patients as frequently observed in patients suffering from other severe NDDs [31–35]. Furthermore, GNAO1 patients have sometimes impaired vision, including nystagmus or strabismus [36].

Approximately 8.0% of the GNAO1 patients succumb prematurely (Supporting Information: Table S3). This number is likely an underrepresentation as the natural history of many diagnosed patients is not available. GNAO1 patients may die of respiratory tract infections and obstructions [1, 37–39], asphyxia due to choking while drinking [39], intestinal infections [37] or obstructions [38], exacerbations of hyperkinesia [23, 24], sudden unexplained death in epilepsy (SUDEP) ([39], this review), or cerebral edema due to cardiac and respiratory dysfunction (Bobylova, personal communication). Besides these symptoms, approximately 42.3% of the GNAO1 patients show morphological brain abnormalities, which sometimes worsen over time [24]. Commonly observed aberrations are atrophy of certain brain areas, thinning of the corpus callosum, and affected myelination (Figure 7(c)).

## 6. Treatment Response, Progression, and Gender Effect in the GNAO1 Disorder

### 6.1. Heterogeneity in Treatment Response and Course of Disease

**Figure 8:**
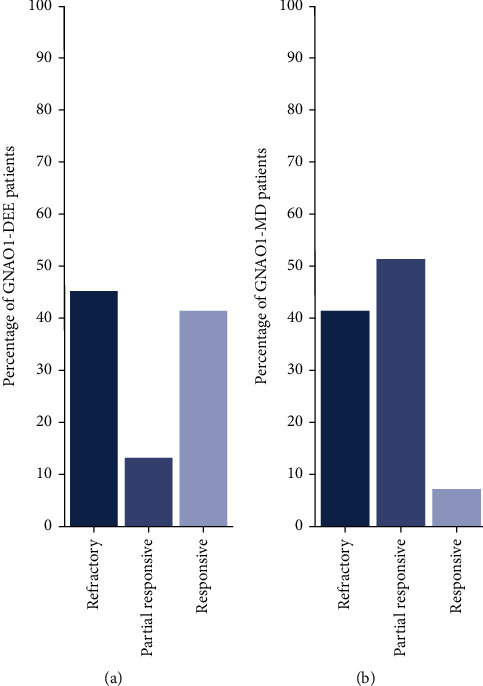
Heterogeneous responses to available medication in GNAO1 patients. (a) Heterogeneity in response to antiepileptic drug (AED) in GNAO1-DEE patients (*n* = 53). (b) Heterogeneous response to medication in GNAO1-MD patients (*n* = 70). Of note, MD is often progressive in GNAO1 patients, and a full response does not necessarily mean a lifelong symptom-free status.

The response of GNAO1 patients to available medication is highly variable and typically poor when considering treatment for DEE and/or severe MD (Figure 8). In contrast, patients who develop epilepsy at a later age respond relatively well to available medication (Supporting Information: Table S3). 60.0% of GNAO1-DEE patients do not or only partially respond to AEDs (Figure 8(a) and Supporting Information: Table S3). This percentage is very similar to other genetic types of DEE, which are often refractory to antiepileptic drugs (AEDs). Intractable seizures occur 1-20 times a day, many of which are long lasting (minutes). Best responses have been observed with either topiramate and/or vigabatrin or zonisamide in combination with lamotrigine. Furthermore, a recent study described the efficient usage of perampanel in four GNAO1 patients with epileptic seizures [40]; however, the *GNAO1* variants and subtypes of epilepsy were not specified. Control of seizures in GNAO1-DEE patients does not appear to be dependent on the genetic variant. For example, p.G203R is one of the genomic mutation hotspots leading to DEE but the responses of p.G203R patients to AEDs are very variable with a response rate of nearly 50.0%. If seizures are not well-controlled, DEE can evolve into severe forms of epilepsy with dangerous exacerbations into status epilepticus. The Ohtahara syndrome can progress further into the West syndrome and eventually into the Lennox-Gastaut syndrome or can transition to severe focal epilepsy. Progression of GNAO1-DEE seems very heterogeneous and difficult to predict with some patients developing Lennox-Gastaut and others going into “spontaneous” remission from seizures, usually after a period of treatment. Unfortunately, with the lack of a complete natural history of the GNAO1-DEE patients, the path of DEE progression is largely unclear. Nevertheless, the monozygotic GNAO1 p.K278del twins (Table 1), which present radically different responses to AEDs, illustrate that treatment response and consequently progression are not only controlled by genetics (Table 1).

Similar to GNAO1-DEE, movement disorder symptoms in GNAO1 patients are mostly poorly controlled with varying responses to different regimens (Figure 7(b)). Treatment responses are independent of the *GNAO1* variant and sex, and only moderate success has been achieved in some patients with treatment using benzodiazepines, trihexyphenidyl, antimuscarinics, adrenergic agonists, gabapentin, neuroleptics, and combinations thereof (Supporting Information: Table S3). Recently, oxcarbazepine treatment showed promising effects in a p.E237K patient [41]. In GNAO1 patients, MD symptoms, such as dystonia and chorea, often progress over time into recurrent episodes of life-threatening pharmacoresistant status dystonicus [42]. The progressive nature of the course of MD likely results in an overestimation of the responsive rate to available medication. Only with a full natural history, the response can be correctly assessed. For patients with life-threatening status dystonicus, emergency deep brain stimulation (DBS) of the globus pallidus internus or subthalamic nucleus is a therapeutic option. Although DBS does not cure the movement disorder, it often (>90.0%) ameliorates the extrapyramidal manifestations and prevents new episodes of status dystonicus [23, 24, 42, 43].

### 6.2. Gender Effect

A gender effect was postulated for the GNAO1 disorder [14, 44]. To test the existence of gender skewing for the GNAO1 disorder, we used the available gender information of 213 patients with *GNAO1A* variants. This analysis reconfirmed the postulated gender effect with a preponderance towards female (56.8% female vs. 43.2% male patients (Binomial distribution *z* = 2.035 one-tailed *p* = .209 (used population distribution of 0.496)). When the patients are grouped according to the typical GNAO1 phenotypes of DEE, epilepsy, MD, or a mixed MD-epilepsy phenotype, the sex ratios are not significantly altered within the GNAO1 patient cohort (lowest *p* value for the higher incidence of DEE in GNAO1 female patients (*Χ*^2^ (1, *N* = 213) = 2.2848, *p* = .13). Although the power for a sex-based analysis of specific variants in mutation hotspots is limited, a female-specific skewing for p.G203R could be observed (*n* = 21; binomial distribution *z* = 2.218911; one-tailed *p* = .013246). In contrast, p.R209 variants are more often observed in male patients, but this is not statistically significant (*n* = 37, binomial distribution *z* = −0.937762, one-tailed *p* = .174183). This phenomenon of gender skewing could be explained by the fact that certain variants may yield either a milder or a more severe phenotype depending on the gender and genetic makeup, e.g., the gender creates nearly subclinical symptoms or provokes a phenotype that is not compatible with life. p.G203R patients exhibit very dramatic phenotypes. The observed skewing towards more female GNAO1 p.G203R patients suggests that male human carriers may exhibit a more severe phenotype. This is corroborated by studies reporting similar gender-based phenotypic differences in *GNAO1* mutant mouse and drosophila models [45–47]. For example, male fruit flies carrying the p.G203R variant have a significantly shorter lifespan than p.G203R females [46].

## 7. Food for Thought

GNAO1 is a very rare disorder with a very heterogeneous display of symptoms. We created a comprehensive GNAO1 database (GNAO_DB) through the collation of publicly available data and information regarding previously undescribed GNAO1 patients. On the basis of the number of identified Dutch GNAO1 disorder patients during the last decennium, we estimate a GNAO1 disorder prevalence of 1 : 100,000-1 : 200,000. This number may be even higher as some *GNAO1* variants may have been overlooked because they were not considered pathogenic due to their rarity or associated with a milder GNAO1 phenotype. Some variants may even be not compatible with life.

All *GNAO1* variants described in this paper will be available at databases.lovd.nl/shared/genes/GNAO1. Basic information on the phenotype data can be found in Table S3. The created GNAO1 database (GNAO_DB) is the most extensive GNAO1 overview to date and contains information on unique and novel *GNAO1* variants. This dataset contains, to the best of our knowledge, only unique patients on the basis of gender, phenotype descriptions, and sequencing cohorts. Continuous updating and addition of novel information are important for improved classification of pathogenicity, diagnosis, and prognosis. We intend to update the GNAO_DB on a regular basis. Incorporation of the natural history of the disorder, which was recently initiated by the Bow Foundation [48] would be extremely invaluable in view of the progressive nature of the GNAO1 disorder.

Since privacy laws like the general data protection regulation (GDPR) restrict the information that is directly publicly available via genome variant databases (e.g. ClinVar), direct input from caregivers and clinicians is essential for keeping the generated and anonymous GNAO_DB updated. Larger databases allow for a better assessment of variants and warrant reevaluation of previously identified variants. Reevaluation should be performed on a regular basis. Using the data collated, Leiden NDD patients were reassessed, which led to the identification of 2 novel GNAO1 patients.

To demonstrate the pathogenicity of rare variants classified as VUS or likely pathogenic variants, the effect on the localization, which is often altered in pathogenic variants [1, 17, 49], or its binding to known interactors [1, 25, 49] should be investigated. With current high-content fluorescence imaging and proximity-based assays using bioluminescence resonance energy transfer (BRET), it would be rather straightforward to determine localization, dominant negative (DN) modes of action, and agonist-induced GPCR-signaling responses for all germline variants.

The presented *GNAO1*-variant dataset has shed some additional light onto the heterogeneity of the GNAO1 disorder in terms of phenotype, treatment response, progression, and disease variants. Phenotypic heterogeneity is extensive between variants but within groups of identical variants as well. Furthermore, this overview emphasizes the mixed phenotypic nature of the disorder with MD or epilepsy appearing at a later onset for some variants. The origin of this later onset remains unclear but once again indicates the urgent need for a full natural history study.

Nonsynonymous variants that cause a severe epileptic and/or MD phenotype are likely to exert a dominant negative (DN) action. Variants may produce a shifted balance in G protein signaling as different variants may interfere with one or multiple stages of the G*α*o signaling cycle decreasing agonist-induced responses at different levels [50], or they may act as a neomorph by establishing a different, novel function. Consequences of AA substitutions are dependent on the position within the protein and include aberrant guanine binding [51], G*βγ* dissociation [19, 20] or association [50], receptor binding, effector binding [19], RGS binding [19, 49, 50], and enhanced degradation [25]. Although variants determine the phenotype to some extent, the varying phenotypes of patients with identical *GNAO1* variants indicate a role for additional factors as well. The observed gender predisposition points to a contribution of the genetic background, which is supported by animal experiments. Furthermore, observed phenotypic discordance in a monozygotic GNAO1 p.K278del twin underscores that epigenetics, probabilistic neuronal connections, or synaptic plasticity may be equally decisive. For studying the contribution of the genetic makeup to the phenotype, patient-derived induced pluripotent stem cell- (iPSC-) based GNAO1 models are needed [52]. Once these epilepsy and MD models have been validated, they can be implemented to study treatment responses, test new medication, and possibly develop GNAO1-tailored precision medication.

Efficient application of precision medicine requires appropriate grouping of the GNAO1 patients on the basis of genetics and phenotypes. Installing a GNAO1 clinical “2^nd^ opinion” board consisting of experts in the field or providing strict phenotyping guidelines would aid in reducing phenotypic diversity. This, in combination with the availability of a complete GNAO1 natural history, would facilitate the grouping of GNAO1 patients and may contribute to our understanding and to more straightforward treatment modalities for this disorder.

## Figures and Tables

**Table 1:** Phenotypes of previously unreported GNAO1 patients.

	Patient 1	Patient 2	Patient 3	Patient 4	Patient 5	Patient 6	Patient 7	Patient 8
Variant	c.833_835del (p.K278del)	c.833_835del (p.K278del)	*c*.687*C* > *A* (p.S229R)	*c*.607*G* > *A* (p.G203R)	*c*.141*C* > *A* (p.S47R)	*c*.625*C* > *T* (p.R209C)	*c*.622*G* > *C* (p.E208Q)	*c*.550*G* > *A* (p.G184S)
Current age	8 years	8 years	Deceased (8 months)	2 years	Deceased (4 years)	13 years	9 years	2 years
Sex	F	F	F	F	M	M	M	M
Age of onset	2 d	2 d	8 d	10 d	<2 months	First weeks	1 d	1 d
Symptoms of onset	Seizure	Seizure	Seizure	Seizure	Seizure	Axial hypotonia and stiffness of limbs	Hypotonia and swallowing difficulties	Hypotonia, feeding difficulties
Epilepsy	Yes	Yes	Yes	Yes	Yes	Yes	No	No
DEE	Yes^∗^	Yes^∗^	Yes	Yes	Yes	No	No	No
MD	Dystonia (8 years)	Dystonia (8 years)	No	Choreoathetosis dystonia (intermittent paroxysmal)	No	Spasticity and dystonia. Recurrent status dystonicus despite medication.	Dystonia	No
Axial hypotonia	Yes	Yes	Yes	Yes	Yes	Yes	Yes	Yes
Treatment								
Epilepsy	TP, DPK, CNZ, LEV	**PB** (seizure free)		**LEV**, **CBZ**, PB, and CLB	LEV, PB, CBZ, VA, PN, VGB, MDZ, LTG, and TPM	**VPA**		
	DRE	Seizure free	DRE	Seizure free	DRE	Seizure free		
MD	No	No	No	DZP, LVD, CH, GBP, CND, and BCF		GBP, LEV, BCF, and CLB	**GBP** and **CNZ**	
						Since **deep brain stimulation**, no more hospital admissions for status dystonicus		
MRI	2 weeks: no abnormalities	2 weeks: no abnormalities	No abnormalities	No abnormalities	2 months: no abnormalities	7 and 11 years: at 11 years compared to 7 years mild widening of the ventricular width and subarachnoid space	5 months, 17 months, and 8 years: stable, aspecific T2 hyperintensities near occipital horn right and bilateral frontally.	

^∗^Suspected DEE as determined from age of onset, EEG data, and presence of developmental delay. Criteria used from [26]. MD = movement disorder; N/A = not available; TP = topiramate; DPK = depakine; CNZ = clonazepam; LEV = levetiracetam; DRE = drug resistant epilepsy; CBZ = carbamazepine; PB = phenobarbital; CLB = clobazam; DZP = diazepam; LVD = levodopa; CH = chloral hydrate; GBP = gabapentin; CND = clonidine; BCF = baclofen; TPM = topiramate; LTG = lamotrigine; **Bold:** mild or fully successful treatment.

## Data Availability

Germline variants were submitted to LOVD (databases.lovd.nl/shared/genes/GNAO1).

## Supplementary Materials

Supplementary Materials Table S1: Missense, nonsense, frameshift, and splicing affecting variants in *GNAO1A* and *GNAO1B* obtained from whole genome and GNAO1 case reports, Dutch and Spanish GNAO1 cohorts, and variant databases (ClinGen, ClinVar, dbSNP55, Decipher, gnomAD, LOVD, TopMed, and VarSome). ^∗^(Likely) pathogenic on the basis of an *in vitro* study [54]. ^∗∗^Pathogenic on the basis of our unpublished functional data. ^∗∗∗^Pathogenic on the basis of multiple NDD patients with the same variant. ^∗∗∗∗^Likely pathogenic on the basis of multiple NDD patients with similar variants. ^∗∗∗∗∗^Pathogenic on the basis of Kehrl et al. [55]. Table S2: Overview of (likely) pathogenic *GNAO1* variants. ^∗^(Likely) pathogenic on the basis of an *in vitro* study [54]. ^∗∗^Pathogenic on the basis of our unpublished functional data. ^∗∗∗^Pathogenic on the basis of multiple NDD patients with the same variant. ^∗∗∗∗^Likely pathogenic on the basis of multiple NDD patients with similar variants. ^∗∗∗∗∗^Pathogenic on the basis of Kehrl et al. [55]. Table S3: Overview of GNAO1 patients and their phenotypes. F: female; M: male; MD: movement disorder; DD: developmental delay; DEE: developmental epileptic encephalopathy; E: epilepsy; AT: atrophy; MY: altered (de)myelination; MC: microcephaly; TCC: thinned corpus callosum; NA: not available.
