# Supplementary material for: Phenotypic Diversity in GNAO1 Patients: A Comprehensive Overview of Variants and Phenotypes
Source: Hum Mutat. 2023 Aug 7;2023:6628283. doi: 10.1155/2023/6628283 (PMC11919132; doi:10.1155/2023/6628283)
Supplement: Supplementary Materials — Table S1: Missense, nonsense, frameshift, and splicing affecting variants in GNAO1A and GNAO1B obtained from whole genome and GNAO1 case reports, Dutch and Spanish GNAO1 cohorts, and variant databases (ClinGen, ClinVar, dbSNP55, Decipher, gnomAD, LOVD, TopMed, and VarSome). ∗(Likely) pathogenic on the basis of an in vitro study [54]. ∗∗Pathogenic on the basis of our unpublished functional data. ∗∗∗Pathogenic on the basis of multiple NDD patients with the same variant. ∗∗∗∗Likely pathogenic on the basis of multiple NDD patients with similar variants. ∗∗∗∗∗Pathogenic on the basis of Kehrl et al. [55]. Table S2: Overview of (likely) pathogenic GNAO1 variants. ∗(Likely) pathogenic on the basis of an in vitro study [54]. ∗∗Pathogenic on the basis of our unpublished functional data. ∗∗∗Pathogenic on the basis of multiple NDD patients with the same variant. ∗∗∗∗Likely pathogenic on the basis of multiple NDD patients with similar variants. ∗∗∗∗∗Pathogenic on the basis of Kehrl et al. [55]. Table S3: Overview of GNAO1 patients and their phenotypes. F: female; M: male; MD: movement disorder; DD: developmental delay; DEE: developmental epileptic encephalopathy; E: epilepsy; AT: atrophy; MY: altered (de)myelination; MC: microcephaly; TCC: thinned corpus callosum; NA: not available. [file 6628283.f1.docx]

**TABLE S1. Missense, nonsense, frameshift and splicing affecting variants in *GNAO1A* and *GNAO1B* obtained from whole genome and GNAO1 case reports, Dutch and Spanish GNAO1 cohorts, and variant databases (ClinGen, ClinVar, dbSNP55, Decipher, gnomAD, LOVD,TopMed and VarSome).**

| **CHR16 POSITION** | **VARIANT** | **rs_dbSNP** | **GENE** | **ENSEMBL_**  **TRANSCRIPT ID** | **POLYPHEN2_**  **HDIV** | **PREDICTION** | **VEST4** | **PREDICTION** | **REVEL** | **PREDICTION** | **BayesDel_**  **addAF** | **PREDICTION** | **ClinPred** | **PREDICTION** | **ACMG classification** |
| --- | --- | --- | --- | --- | --- | --- | --- | --- | --- | --- | --- | --- | --- | --- | --- |
| 56192238 | c.3G>T (p.Met1Ile) | . | *GNAO1A* | ENST00000262493 | 0.991 | D | 0.763 | D | 0.926 | D | 0.625005 | D | 0.997799694538116 | D | Likely Pathogenic (VarSome) |
| 56192243 | c.8G>A (p.Cys3Tyr) | . | *GNAO1A* | ENST00000262493 | 1.0 | D | 0.64 | D | 0.913 | D | 0.493513 | D | 0.999862432479858 | D | Uncertain Significance (ClinVar)*not de novo,no phenotype father (likely benign) |
| 56192267 | c.32C>T (p.Ala11Val) | rs1380348729 | *GNAO1A* | ENST00000262493 | 0.495 | P | 0.34 | B | 0.514 | D | 0.110877 | D | 0.673199355602264 | D | Benign |
| 56192273 | c.38T>C (p.Leu13Pro) | rs1555499768 | *GNAO1A* | ENST00000262493 | 0.007 | B | 0.337 | B | 0.385 | B | 0.0979789 | D | 0.805835008621216 | D | Likely Pathogenic (ClinVar, Ensembl) |
| 56192293 | c.58G>A (p.Glu20Lys) | rs2036182701 | *GNAO1A* | ENST00000262493 | 0.779 | B | 0.443 | B | 0.842 | D | 0.297882 | D | 0.985042154788971 | D | Likely Pathogenic, Uncertain Significance (ClinVar) |
| 56192303 | c.68T > C (p.Leu23Pro) | . | *GNAO1A* | ENST00000262493 | 1.0 | D | 0.867 | D | 0.973 | D | 0.475924 | D | 0.999778807163239 | D | Likely Pathogenic |
| 56192311 | c.76G>A (p.Asp26Asn) | . | *GNAO1A* | ENST00000262493 | 0.039 | B | 0.609 | D | 0.647 | D | 0.065968 | T | 0.989794671535492 | D | Uncertain Significance (ClinVar) |
| 56192324 | c.89C>T (p.Ala30Val) | . | *GNAO1A* | ENST00000262493 | 1.0 | D | 0.507 | D | 0.698 | D | 0.14801 | D | 0.980357348918915 | D | Uncertain Significance (ClinVar) |
| 56192329 | c.94A>G (p.Lys32Glu) | rs2036182956 | *GNAO1A* | ENST00000262493 | 0.951 | P | 0.33 | B | 0.751 | D | 0.156931 | D | 0.963409841060638 | D | Uncertain Significance (VarSome) |
| 56192335 | c.100G>A (p.Val34Met) | . | *GNAO1A* | ENST00000262493 | 0.999 | D | 0.584 | D | 0.878 | D | 0.307797 | D | 0.995852589607239 | D | Uncertain Significance (VarSome) |
| 56192335 | c.100G>C (p.Val34Leu) | rs1567431805 | *GNAO1A* | ENST00000262493 | 0.541 | P | 0.638 | D | 0.703 | P | 0.20632 | D | 0.970898747444153 | D | Uncertain Significance (VarSome) |
| 56192343 | c.108A>C (p.Leu36Phe) | rs1284492592 | *GNAO1A* | ENST00000262493 | 1.0 | D | 0.635 | D | 0.829 | D | 0.328709 | D | 0.994408667087555 | D | Uncertain Significance (VarSome) |
| 56192346 | c.111_113delCCT (p.Leu39del) | . | *GNAO1A* | ENST00000262493 |  |  |  |  |  |  |  |  |  |  | Likely Pathogenic (Decipher) |
| 56192350 | c.115C>T (p.Leu39Phe) | . | *GNAO1A* | ENST00000262493 | 1.0 | D | 0.61 | D | 0.931 | D | 0.443912 | D | 0.998496890068054 | D | Uncertain Significance (ClinVar) |
| 56192351 | c.116T>C (p.Leu39Pro) | rs1555499769 | *GNAO1A* | ENST00000262493 | 1.0 | D | 0.919 | D | 0.971 | D | 0.534903 | D | 0.99897837638855 | D | Likely Pathogenic, Uncertain Significance (ClinVar, Ensembl) |
| 56192353 | c.118G>A (p.Gly40Arg) | rs886041715 | *GNAO1A* | ENST00000262493 | 1.0 | D | 0.947 | D | 0.983 | D | 0.583003 | D | 0.999903678894043 | D | Pathogenic (ClinVar) |
| 56192353 | c.118G>C (p.Gly40Arg) | rs886041715 | *GNAO1A* | ENST00000262493 | 1.0 | D | 0.947 | D | 0.983 | D | 0.583003 | D | 0.999881267547607 | D | Pathogenic (ClinVar) |
| 56192353 | c.118G>T (p.Gly40Trp) | rs886041715 | *GNAO1A* | ENST00000262493 | 1.0 | D | 0.878 | D | 0.978 | D | 0.583003 | D | 0.99984085559845 | D | Pathogenic (ClinVar) |
| 56192574 | c.119G>A (p.Gly40Glu) | rs886041766 | *GNAO1A* | ENST00000262493 | 1.0 | D | 0.949 | D | 0.996 | D | 0.584152 | D | 0.999758899211884 | D | Pathogenic (ClinVar) |
| 56192574 | c.119G>T (p.Gly40Val) | rs886041766 | *GNAO1A* | ENST00000262493 | 1.0 | D | 0.948 | D | 0.995 | D | 0.584285 | D | 0.999819576740265 | D | Pathogenic (ClinVar) |
| 56192576 | c.121G>A (p.Ala41Thr) | rs2036187623 | *GNAO1A* | ENST00000262493 | 0.937 | D | 0.432 | B | 0.698 | D | 0.143445 | D | 0.99322634935379 | D | Uncertain Significance (ClinVar) |
| 56192579 | c.124G​>C (p.Gly42Arg) | . | *GNAO1A* | ENST00000262493 | 1.0 | D | 0.623 | D | 0.955 | D | 0.423286 | D | 0.9992795586586 | D | Pathogenic (ClinVar) |
| 56192579 | c.124G>A (p.Gly42Arg) | . | *GNAO1A* | ENST00000262493 | 1.0 | D | 0.623 | D | 0.955 | D | 0.423286 | D | 0.999132335186005 | D | Pathogenic (ClinVar) |
| 56192582 | c.127G>C (p.Glu43Gln) | rs2036187686 | *GNAO1A* | ENST00000262493 | 1.0 | D | 0.674 | D | 0.856 | D | 0.266186 | D | 0.999523520469666 | D | Likely Pathogenic (VarSome) |
| 56192588 | c.133G>C (p.Gly45Arg) | rs869312939 | *GNAO1A* | ENST00000262493 | 1.0 | D | 0.954 | D | 0.985 | D | 0.58415 | D | 0.999623537063599 | D | Pathogenic (ClinVar) |
| 56192589 | c.134G>A (p.Gly45Glu) | . | *GNAO1A* | ENST00000262493 | 1.0 | D | 0.969 | D | 0.980 | D | 0.584148 | D | 0.999809324741364 | D | Pathogenic |
| 56192591 | c.136A>G (p.Lys46Glu) | . | *GNAO1A* | ENST00000262493 | 1.0 | D | 0.819 | D | 0.963 | D | 0.578381 | D | 0.99932587146759 | D | Likely Pathogenic (ClinVar) |
| 56192592 | c.137A>G (p.Lys46Arg) | . | *GNAO1A* | ENST00000262493 | 1.0 | D | 0.72 | D | 0.948 | D | 0.548404 | D | 0.999465882778168 | D | Likely Pathogenic (Community Classification) |
| 56192593 | c.138A>T (p.Lys46Asn) | . | *GNAO1A* | ENST00000262493 | 1.0 | D | 0.805 | D | 0.898 | D | 0.3585 | D | 0.999612152576447 | D | Likely Pathogenic (ClinVar) |
| 56192594 | c.139A>G (p.Ser47Gly) | . | *GNAO1A* | ENST00000262493 | 1.0 | D | 0.795 | D | 0.950 | D | 0.484377 | D | 0.999099254608154 | D | Pathogenic |
| 56192595 | c.140G>A (p.Ser47Asn) | rs1596787821 | *GNAO1A* | ENST00000262493 | 0.999 | D | 0.807 | D | 0.855 | D | 0.222452 | D | 0.999606072902679 | D | Pathogenic (ClinVar) |
| 56192596 | c.141C>A (p.Ser47Arg) | . | *GNAO1A* | ENST00000262493 | 1.0 | D | 0.946 | D | 0.914 | D | 0.470333 | D | 0.999834179878235 | D | Likely Pathogenic (Community Classification) |
| 56192598 | c.143C>A (p.Thr48Asn) | rs1555499800 | *GNAO1A* | ENST00000262493 | 1.0 | D | 0.903 | D | 0.888 | D | 0.220326 | D | 0.999808013439178 | D | Likely Pathogenic (ClinVar) |
| 56192598 | c.143C>T (p.Thr48Ile) | rs1555499800 | *GNAO1A* | ENST00000262493 | 1.0 | D | 0.935 | D | 0.939 | D | 0.485927 | D | 0.99985146522522 | D | Likely Pathogenic (ClinVar) |
| 56192600 | c.145A>T (p.Ile49Phe) | rs763223472 | *GNAO1A* | ENST00000262493 | 1.0 | D | 0.77 | D | 0.809 | D | 0.296064 | D | 0.948225617408752 | D | Uncertain Significance (VarSome) |
| 56192600 | c.145A>C (p.Ile49Leu) | . | *GNAO1A* | ENST00000262493 | 0.949 | P | 0.633 | D | 0.761 | D | 0.150333 | D | 0.991604268550873 | D | Uncertain Significance (ClinVar) |
| 56192606 | c.151A>G (p.Lys51Glu) | . | *GNAO1A* | ENST00000262493 | 1.0 | D | 0.778 | D | 0.926 | D | 0.505456 | D | 0.999284446239471 | D | Uncertain Significance (ClinVar) |
| 56192610 | c.155A>C (p.Gln52Pro) | . | *GNAO1A* | ENST00000262493 | 1.0 | D | 0.917 | D | 0.955 | D | 0.516164 | D | 0.999384164810181 | D | Pathogenic |
| 56192610 | c.155A>G (p.Gln52Arg) | . | *GNAO1A* | ENST00000262493 | 0.999 | D | 0.871 | D | 0.938 | D | 0.510894 | D | 0.999569118022919 | D | Pathogenic |
| 56192613 | c.158T>A (p.Met53Lys) | . | *GNAO1A* | ENST00000262493 | 0.993 | B | 0.853 | D | 0.957 | D | 0.476985 | D | 0.999065220355988 | D | Uncertain Significance (ClinVar) |
| 56275933 | c.164T>A (p.Ile55Asn) | . | *GNAO1A* | ENST00000262493 | 0.999 | D | 0.944 | D | 0.948 | D | 0.349024 | D | 0.998652160167694 | D | Uncertain Significance (LOVD) |
| 56275936 | c.167T>C (p.Ile56Thr) | . | *GNAO1A* | ENST00000262493 | 0.917 | P | 0.848 | D | 0.951 | D | 0.47459 | D | 0.995525538921356 | D | Likely Pathogenic (Community Classification) |
| 56275939 | c.170A>C (p.His57Pro) | . | *GNAO1A* | ENST00000262493 | 0.026 | B | 0.91 | D | 0.882 | D | 0.481845 | D | 0.99908185005188 | D | Uncertain Significance |
| 56275956 | c.187G>A (p.Gly63Arg) | rs940833242 | *GNAO1A* | ENST00000262493 | 0.014 | B | 0.693 | D | 0.446 | B | 0.31268 | D | 0.542759239673615 | D | Uncertain Significance (ClinVar) |
| 56275965 | c.196G>A (p.Val66Met) | rs557932562 | *GNAO1A* | ENST00000262493 | 0.911 | P | 0.459 | B | 0.552 | D | 0.0946899 | D | 0.417904585599899 | T | Uncertain Significance (VarSome) |
| 56275976 | c.207C>A (p.Tyr69Ter) | rs77558236 | *GNAO1A* | ENST00000262493 |  |  | 0.89 | D |  |  | 0.625005 | D |  |  | Likely Pathogenic (VarSome) |
| 56275983 | c.214G>A (p.Val72Ile) | rs371330886 | *GNAO1A* | ENST00000262493 | 0.711 | P | 0.5 | D | 0.431 | B | 0.127142 | D | 0.651444733142853 | D | Uncertain Significance (VarSome) |
| 56275987 | c.218T>A (p.Val73Asp) | . | *GNAO1A* | ENST00000262493 | 1.0 | D | 0.944 | D | 0.964 | D | 0.490729 | D | 0.999195277690887 | D | Uncertain Significance |
| 56275989 | c.220T>G (p.Tyr74Asp) | . | *GNAO1A* | ENST00000262493 | 0.883 | P | 0.942 | D | 0.932 | D | 0.463288 | D | 0.99783331155777 | D | Pathogenic (LOVD) |
| 56275993 | c.224G>A (p.Ser75Asn) | rs2037057993 | *GNAO1A* | ENST00000262493 | 0.941 | P | 0.76 | D | 0.525 | D | 0.108103 | D | 0.911368131637573 | D | Uncertain Significance (VarSome) |
| 56275998 | c.229A>C (p.Thr77Pro) | rs1596836465 | *GNAO1A* | ENST00000262493 | 0.979 | D | 0.698 | D | 0.933 | D | 0.47355 | D | 0.995821714401245 | D | Likely Pathogenic (VarSome) |
| 56276001 | c.232A>G (p.Ile78Val) | rs1263683170 | *GNAO1A* | ENST00000262493 | 0.002 | B | 0.419 | B | 0.320 | B | 0.124381 | D | 0.686370730400085 | D | Uncertain Significance (VarSome) |
| 56276001 | c.232A>C (p.Ile78Leu) | . | *GNAO1A* | ENST00000262493 | 0.001 | B | 0.703 | D | 0.497 | B | 0.211455 | D | 0.890168130397797 | D | Likely benign/Uncertain Significance (ClinVar) |
| 56276013 | c.244G>A (p.Ala82Thr) | rs1198213656 | *GNAO1A* | ENST00000262493 | 0.009 | B | 0.598 | D | 0.394 | B | 0.196477 | D | 0.773366272449493 | D | Uncertain Significance (VarSome) |
| 56276022 | c.253G>A(p.Val85Ile) | rs903629384 | *GNAO1A* | ENST00000262493 | 0.0 | B | 0.349 | B | 0.337 | B | 0.0208047 | T | 0.770415246486664 | D | Uncertain Significance (VarSome) |
| 56276025 | c.256C>T (p.Arg86Trp) | rs767115578 | *GNAO1A* | ENST00000262493 | 1.0 | D | 0.692 | D | 0.877 | D | 0.328509 | D | 0.995086848735809 | D | Uncertain Significance (VarSome) |
| 56276026 | c.257G>A (p.Arg86Gln) | rs1301302019 | *GNAO1A* | ENST00000262493 | 0.989 | D | 0.585 | D | 0.738 | D | 0.331547 | D | 0.98668110370636 | D | Likely benign (ClinVar) |
| 56276031 | c.262A>G (p.Met88Val) | rs1555504233 | *GNAO1A* | ENST00000262493 | 0.932 | D | 0.756 | D | 0.921 | D | 0.551664 | D | 0.978673160076141 | D | Uncertain Significance (ClinVar) |
| 56276032 | c.263T>C (p.Met88Thr ) | rs1221678868 | *GNAO1A* | ENST00000262493 | 0.83 | P | 0.912 | D | 0.962 | D | 0.568033 | D | 0.99631279706955 | D | Uncertain Significance (Ensembl) |
| 56276032 | c.263T>G (p.Met88Arg ) | rs1221678868 | *GNAO1A* | ENST00000262493 | 0.385 | B | 0.941 | D | 0.920 | D | 0.537978 | D | 0.990505993366241 | D | Uncertain Significance (ClinVar) |
| 56276036 | c.267C>A (p.Asp89Glu ) | rs201728736 | *GNAO1A* | ENST00000262493 | 0.0 | B | 0.656 | D | 0.471 | B | 0.210305 | D | 0.224239513278008 | T | Uncertain Significance (VarSome) |
| 56276043 | c.274G>A (p.Gly92Ser) | rs1309389793 | *GNAO1A* | ENST00000262493 | 0.003 | B | 0.561 | D | 0.388 | B | 0.296993 | D | 0.925066471099854 | D | Uncertain Significance (ClinVar) |
| 56276044 | c.275G>A (p.Gly92Asp) | rs1271540719 | *GNAO1A* | ENST00000262493 | 0.004 | B | 0.633 | D | 0.459 | B | 0.330245 | D | 0.85084080696106 | D | Uncertain Significance (VarSome) |
| 56276046 | c.277A>G (p.Ile93Val) | rs2037058607 | *GNAO1A* | ENST00000262493 | 0.0 | B | 0.158 | B | 0.337 | B | 0.0622212 | T | 0.41108649969101 | T | Uncertain Significance (VarSome) |
| 56276049 | c.280G>A (p.Glu94Lys) | rs2037058679 | *GNAO1A* | ENST00000262493 | 0.303 | B | 0.624 | D | 0.567 | D | 0.308072 | D | 0.876787543296814 | D | Uncertain Significance (VarSome) |
| 56276053 | c.284A>G (p.Tyr95Cys) | rs1341492901 | *GNAO1A* | ENST00000262493 | 0.963 | B | 0.721 | D | 0.750 | D | 0.322851 | D | 0.990220129489899 | D | Uncertain Significance (ClinVar) |
| 56276055 | c.286G>A (p.Gly96Ser) | rs559674838 | *GNAO1A* | ENST00000262493 | 0.0 | B | 0.376 | B | 0.403 | B | 0.0686023 | T | 0.0940947309136391 | T | Likely Benign (ClinVar) |
| 56276056 | c.287G>A (p.Gly96Asp) | rs189990922 | *GNAO1A* | ENST00000262493 | 0.043 | B | 0.544 | D | 0.544 | D | 0.367585 | D | 0.618669867515564 | D | Uncertain Significance (VarSome)) |
| 56276056 | c.287G>C (p.Gly96Ala) | rs189990922 | *GNAO1A* | ENST00000262493 | 0.0 | B | 0.363 | B | 0.267 | B | 0.155798 | D | 0.180160524086437 | T | Uncertain Significance (VarSome)) |
| 56276059 | c.290A>G (p.Asp97Gly) | rs778669032 | *GNAO1A* | ENST00000262493 | 0.01 | B | 0.521 | D | 0.735 | D | 0.398418 | D | 0.920802533626556 | D | Uncertain Significance (ClinVar) |
| 56276059 | c.290A>T (p.Asp97Val) | rs778669032 | *GNAO1A* | ENST00000262493 | 0.021 | B | 0.63 | D | 0.748 | D | 0.365347 | D | 0.859552979469299 | D | Uncertain Significance (Ensembl) |
| 56328636 | c.309C>A (p.Asp103Glu) | rs372103298 | *GNAO1A* | ENST00000262493 | 0.113 | B | 0.666 | D | 0.533 | D | 0.188952 | D | 0.945050358772278 | D | Likely Benign (VarSome) |
| 56328637 | c.310G>A (p.Ala104Thr) | rs375429245 | *GNAO1A* | ENST00000262493 | 0.304 | B | 0.419 | B | 0.699 | D | 0.0865021 | D | 0.959694802761078 | D | Uncertain Significance (ClinVar) |
| 56328645 | c.318G>C (p.Met106Ile) | rs978834925 | *GNAO1A* | ENST00000262493 | 0.0 | B | 0.511 | D | 0.310 | B | 0.0414259 | T | 0.516014695167542 | D | Uncertain Significance (ClinVar) |
| 56328646 | c.319G>C (p.Val107Leu) | rs777981381 | *GNAO1A* | ENST00000262493 | 0.0 | B | 0.294 | B | 0.367 | B | 0.123949 | D | 0.134785398840904 | T | Uncertain Significance (VarSome) |
| 56328650 | c.323G>A (p.Cys108Tyr) | rs1355385627 | *GNAO1A* | ENST00000262493 | 0.0 | B | 0.817 | D | 0.523 | D | 0.437743 | D | 0.252919435501099 | T | Uncertain Significance (VarSome) |
| 56328653 | c.326A>T (p.Asp109Val) | rs2037659126 | *GNAO1A* | ENST00000262493 | 0.0 | B | 0.489 | B | 0.556 | D | 0.443926 | D | 0.817804872989655 | D | Uncertain Significance (ClinVar) |
| 56328661 | c.334A>G (p.Ser112Gly) | rs2037659241 | *GNAO1A* | ENST00000262493 | 0.0 | B | 0.276 | B | 0.342 | B | 0.0769396 | D | 0.839843511581421 | D | Uncertain Significance (VarSome) |
| 56328665 | c.338G>A (p.Arg113Gln) | rs956210011 | *GNAO1A* | ENST00000262493 | 0.41 | B | 0.402 | B | 0.424 | B | 0.188168 | D | 0.791401565074921 | D | Uncertain Significance (ClinVar, Ensembl) |
| 56328670 | c.343G>A (p.Glu115Lys) | . | *GNAO1A* | ENST00000262493 | 0.01 | B | 0.803 | D | 0.689 | D | 0.344362 | D | 0.963649034500122 | D | Benign (ClinVar) |
| 56328681 | c.354G>C (p.Glu118Asp) | rs1567485534 | *GNAO1A* | ENST00000262493 | 0.004 | B | 0.395 | B | 0.354 | B | 0.0178021 | T | 0.547942101955414 | D | Likely benign (ClinVar) |
| 56328681 | c.354G>T (p.Glu118Asp) | rs1567485534 | *GNAO1A* | ENST00000262493 | 0.004 | B | 0.395 | B | 0.354 | B | 0.0178021 | T | 0.547942101955414 | D | Likely benign (ClinVar) |
| 56328685 | c.358T>G (p.Phe120Val) | rs2037659527 | *GNAO1A* | ENST00000262493 | 0.001 | B | 0.58 | D | 0.505 | D | 0.0999294 | D | 0.862493932247162 | D | Uncertain Significance (ClinVar) |
| 56328696 | c.369G>T (p.Glu123Asp) | rs776163992 | *GNAO1A* | ENST00000262493 | 0.0 | B | 0.532 | D | 0.476 | B | 0.176643 | D | 0.226936042308807 | T | Uncertain Significance (VarSome) |
| 56328704 | c.377C>G (p.Ser126Cys) | rs1338335814 | *GNAO1A* | ENST00000262493 | 0.191 | B | 0.473 | B | 0.502 | D | 0.0893628 | D | 0.928629457950592 | D | Uncertain Significance (VarSome) |
| 56328706 | c.379G>T (p.Ala127Ser) | rs2037659641 | *GNAO1A* | ENST00000262493 | 0.003 | B | 0.531 | D | 0.459 | B | 0.113131 | D | 0.937630832195282 | D | Uncertain Significance (VarSome) |
| 56328707 | c.380C>A (p.Ala127Asp) | rs1469972348 | *GNAO1A* | ENST00000262493 | 0.903 | P | 0.855 | D | 0.820 | D | 0.371519 | D | 0.981389105319977 | D | Uncertain Significance (VarSome) |
| 56328711 | c.384G>T (p.Met128Ile) | rs1344023119 | *GNAO1A* | ENST00000262493 | 0.003 | B | 0.456 | B | 0.324 | B | 0.059637 | T | 0.466526746749878 | T | Uncertain Significance (VarSome) |
| 56328713 | c.386T>C (p.Met129Thr) | rs200539902 | *GNAO1A* | ENST00000262493 | 0.001 | B | 0.415 | B | 0.342 | B | -0.104529 | T | 0.20033660531044 | T | Likely benign (ClinVar) |
| 56328714 | c.387G>A (p.Met129Ile) | rs2037659789 | *GNAO1A* | ENST00000262493 | 0.0 | B | 0.205 | B | 0.298 | B | 0.0154887 | T | 0.87339198589325 | D | Uncertain Significance (VarSome) |
| 56328715 | c.388C>G (p.Arg130Gly) | rs200127285 | *GNAO1A* | ENST00000262493 | 0.997 | D | 0.595 | D | 0.672 | D | 0.0615396 | T | 0.514902055263519 | D | Uncertain Significance (Ensembl) |
| 56328715 | c.388C>T (p.Arg130Trp) | rs539662922 | *GNAO1A* | ENST00000262493 | 1.0 | D | 0.676 | D | 0.862 | D | 0.251801 | D | 0.970991909503937 | D | Uncertain Significance (ClinVar) |
| 56328716 | c.389G>A (p.Arg130Gln) | rs200127285 | *GNAO1A* | ENST00000262493 | 0.997 | D | 0.595 | D | 0.672 | D | 0.0615396 | T | 0.514902055263519 | D | Likely benign* |
| 56328723 | c.396G>T (p.Trp132Cys) | rs1293266907 | *GNAO1A* | ENST00000262493 | 1.0 | D | 0.942 | P | 0.897 | P | 0.537329 | D | 0.999981164932251 | D | Likely Pathogenic (VarSome) |
| 56328724 | c.397G>C (p.Gly133Arg) | rs374115337 | *GNAO1A* | ENST00000262493 | 1.0 | B | 0.162 | B | 0.286 | B | -0.113063 | T | 0.214147761609297 | T | Uncertain Significance (ClinVar, Ensembl) |
| 56328724 | c.397G>A (p.Gly133Ser) | rs374115337 | *GNAO1A* | ENST00000262493 | 0.0 | B | 0.405 | B | 0.330 | B | 0.0535428 | T | 0.641963005065918 | D | Benign (ClinVar) |
| 56328727 | c.400G>A (p.Asp134Asn) | rs921565958 | *GNAO1A* | ENST00000262493 | 1.0 | D | 0.898 | D | 0.808 | D | 0.193117 | D | 0.99804013967514 | D | Likely Pathogenic (VarSome) |
| 56328730 | c.403T>C (p.Ser135Pro) | rs2037660138 | *GNAO1A* | ENST00000262493 | 0.0 | B | 0.643 | D | 0.390 | B | 0.158184 | D | 0.55029034614563 | D | Uncertain Significance (VarSome) |
| 56328739 | c.412C>G (p.Gln138Glu) | rs759081912 | *GNAO1A* | ENST00000262493 | 0.939 | B | 0.748 | D | 0.852 | D | 0.268808 | D | 0.904673337936401 | D | Uncertain Significance (ClinVar) |
| 56328740 | c.413A>G (p.Gln138Arg) | rs1245351159 | *GNAO1A* | ENST00000262493 | 0.065 | B | 0.748 | D | 0.794 | D | 0.379856 | D | 0.950981318950653 | D | Uncertain Significance (ClinVar) |
| 56328742 | c.415G>A (p.Glu139Lys) | rs1464565646 | *GNAO1A* | ENST00000262493 | 0.154 | B | 0.53 | D | 0.471 | B | 0.226948 | D | 0.906914114952087 | D | Uncertain Significance (VarSome) |
| 56328751 | c.424A>C (p.Asn142His) | rs1273554289 | *GNAO1A* | ENST00000262493 | 0.001 | B | 0.311 | B | 0.250 | B | -0.0271106 | T | 0.833252787590027 | D | Uncertain Significance (VarSome) |
| 56328752 | c.425A>G (p.Asn142Ser) | rs758424351 | *GNAO1A* | ENST00000262493 | 0.005 | B | 0.362 | B | 0.194 | B | -0.00238337 | T | 0.840330481529236 | D | Benign (ClinVar, Ensembl) |
| 56328752 | c.425A>C (p.Asn142Thr) | . | *GNAO1A* | ENST00000262493 | 0.0 | B | 0.189 | B | 0.319 | B | -0.307636 | T | 0.068850218993253 | T | Uncertain Significance |
| 56328754 | c.427C>T (p.Arg143Trp) | . | *GNAO1A* | ENST00000262493 | 1.0 | D | 0.853 | D | 0.873 | D | 0.530943 | D | 0.999358832836151 | D | Likely Benign (ClinVar) |
| 56328755 | c.428G>A (p.Arg143Gln) | rs777414554 | *GNAO1A* | ENST00000262493 | 1.0 | D | 0.841 | D | 0.809 | D | 0.388859 | D | 0.997363388538361 | D | Uncertain Significance (ClinVar, Ensembl) |
| 56328755 | c.428G>T (p.Arg143Leu) | . | *GNAO1A* | ENST00000262493 | 1.0 | D | 0.903 | D | 0.862 | D | 0.578912 | D | 0.996455729007721 | D | Uncertain Significance (ClinVar) |
| 56328760 | c.432C>T (p.Arg145Trp) | rs757388426 | *GNAO1A* | ENST00000262493 | 1.0 | D | 0.725 | D | 0.843 | D | 0.148431 | D | 0.998009741306305 | D | Uncertain Significance (ClinVar) |
| 56328770 | c.442A>C (p.Cys148Gln) | rs2037660606 | *GNAO1A* | ENST00000262493 | 0.999 | D | 0.918 | D | 0.955 | D | 0.485291 | D | 0.998203039169312 | D | Likely Pathogenic (VarSome) |
| 56328775 | c.448A>C (p.Asn150His) | rs2037660713 | *GNAO1A* | ENST00000262493 | 0.506 | B | 0.849 | D | 0.776 | D | 0.148829 | D | 0.996348440647125 | D | Conflicting Interpretation (ClinVar)/ Likely benign* |
| 56328776 | c.449A>G (p.Asn150Ser) | . | *GNAO1A* | ENST00000262493 | 0.931 | P | 0.73 | D | 0.630 | D | 0.0194437 | T | 0.983788013458252 | D | Uncertain Significance |
| 56328778 | c.451G>A (p.Asp151Asn) | rs1596867702 | *GNAO1A* | ENST00000262493 | 1.0 | D | 0.902 | D | 0.768 | D | 0.237639 | D | 0.999461114406586 | D | Likely Pathogenic (ClinVar, Decipher) |
| 56328784 | c.457G>A (p.Ala153Thr) | rs1348698138 | *GNAO1A* | ENST00000262493 | 1.0 | D | 0.88 | D | 0.760 | D | 0.323597 | D | 0.978304028511047 | D | Uncertain Significance |
| 56328787 | c.460A>C (p.Lys154Gln) | rs1056430816 | *GNAO1A* | ENST00000262493 | 0.001 | B | 0.192 | B | 0.279 | B | -0.0193609 | T | 0.530112326145172 | D | Uncertain Significance (VarSome) |
| 56328792 | c.464+2T>C | . | *GNAO1A* | ENST00000262493 |  |  |  |  |  |  |  |  |  |  | Uncertain Significance (ClinVar) |
| 56328795 | c.464+5G>A | . | *GNAO1A* | ENST00000262493 |  |  |  |  |  |  |  |  |  |  | Uncertain Significance (ClinVar) |
| 56334726 | c.465-3C>T | rs1596871341 | *GNAO1A* | ENST00000262493 |  |  |  |  |  |  |  |  |  |  | Uncertain Significance (ClinVar) |
| 56334734 | c.470T​>C (p.Leu157Pro) | . | *GNAO1A* | ENST00000262493 | 1.0 | D | 0.985 | D | 0.981 | D | 0.520554 | D | 0.999674439430237 | D | Likely Pathogenic (ClinVar) |
| 56334737 | c.473A>G (p.Asp158Gly) | . | *GNAO1A* | ENST00000262493 | 0.996 | D | 0.811 | D | 0.936 | D | 0.451148 | D | 0.98500919342041 | D | Likely benign* |
| 56334742 | c.478C>G (p.Leu160Val) | rs1454558144 | *GNAO1A* | ENST00000262493 | 0.962 | D | 0.678 | D | 0.756 | D | 0.32109 | D | 0.870830893516541 | D | Uncertain Significance (VarSome) |
| 56334743 | c.479T>C (p.Leu160Pro) | . | *GNAO1A* | ENST00000262493 | 1.0 | D | 0.983 | D | 0.950 | D | 0.497584 | D | 0.999093532562256 | D | Uncertain Significance (ClinVar) |
| 56334747 | c.483T>A (p.Asp161Glu) | rs1596871381 | *GNAO1A* | ENST00000262493 | 0.002 | B | 0.207 | B | 0.289 | B | 0.0109761 | T | 0.466804414987564 | T | Uncertain Significance (VarSome) |
| 56334749 | c.485G>A (p.Arg162Gln) | rs1240134140 | *GNAO1A* | ENST00000262493 | 0.947 | P | 0.948 | D | 0.878 | D | 0.49214 | D | 0.998919010162354 | D | Likely Pathogenic (VarSome) |
| 56334752 | c.488T>C (p.Ile163Thr) | rs760544764 | *GNAO1A* | ENST00000262493 | 0.485 | B | 0.955 | D | 0.925 | D | 0.447623 | D | 0.987835228443146 | D | Uncertain Significance (ClinVar) |
| 56334754 | c.490G>T (p.Gly164Trp) | rs1182604320 | *GNAO1A* | ENST00000262493 | 1.0 | D | 0.773 | D | 0.714 | D | 0.349973 | D | 0.943167269229889 | D | Uncertain Significance (VarSome) |
| 56334755 | c.491G>T (p.Gly164Val) | rs1029083149 | *GNAO1A* | ENST00000262493 | 1.0 | D | 0.663 | D | 0.703 | D | 0.286146 | D | 0.984663784503937 | D | Uncertain Significance (VarSome) |
| 56334757 | c.493G>C (p.Ala165Pro) | rs1159636167 | *GNAO1A* | ENST00000262493 | 0.658 | P | 0.675 | D | 0.537 | D | 0.0694922 | D | 0.65187659731989 | D | Uncertain Significance (VarSome) |
| 56334758 | c.494C>T (p.Ala165Val) | rs2037724184 | *GNAO1A* | ENST00000262493 | 0.159 | B | 0.551 | D | 0.364 | B | 0.0790031 | D | 0.776074886322021 | D | Uncertain Significance (VarSome) |
| 56334760 | c.496G>A (p.Ala166Thr) | rs375960435 | *GNAO1A* | ENST00000262493 | 0.002 | B | 0.095 | B | 0.187 | B | -0.0903357 | T | 0.0450742546975113 | T | Benign (ClinVar) |
| 56334763 | c.499G>A (p.Asp167Asn) | rs2037724346 | *GNAO1A* | ENST00000262493 | 0.006 | B | 0.561 | D | 0.322 | B | 0.057568 | T | 0.611036366111296 | D | Uncertain Significance (VarSome) |
| 56334763 | c.499G>C (p.Asp167His) | . | *GNAO1A* | ENST00000262493 | 0.897 | B | 0.686 | D | 0.764 | D | 0.373624 | D | 0.988095641136169 | D | Uncertain Significance (ClinVar) |
| 56334771 | c.507G>C (p.Gln169His) | rs762554588 | *GNAO1A* | ENST00000262493 | 0.001 | B | 0.531 | D | 0.467 | B | 0.169238 | D | 0.671993434429169 | D | Uncertain Significance (VarSome) |
| 56334773 | c.509C>G (p.Pro170Arg) | . | *GNAO1A* | ENST00000262493 | 1.0 | D | 0.985 | D | 0.961 | D | 0.580692 | D | 0.999860167503357 | D | Likely Pathogenic (ClinVar) |
| 56334776 | c.512C>A (p.Thr171Asn) | rs763888697 | *GNAO1A* | ENST00000262493 | 0.167 | B | 0.582 | D | 0.583 | D | 0.11485 | D | 0.556119203567505 | D | Uncertain Significance (VarSome) |
| 56334784 | c.520G>A (p.Asp174Asn) | rs1567488305 | *GNAO1A* | ENST00000262493 | 1.0 | D | 0.974 | D | 0.933 | D | 0.259532 | D | 0.999670028686523 | D | Pathogenic*** |
| 56334785 | c.521A>G (p.Asp174Gly) | rs587777055 | *GNAO1A* | ENST00000262493 | 1.0 | D | 0.985 | D | 0.988 | D | 0.559195 | D | 0.999401688575745 | D | Pathogenic |
| 56334791 | c.527_528delinsAA (p.Leu176Gln) | rs2037724743 | *GNAO1A* | ENST00000262493 | 1.0 | D |  |  |  |  |  |  |  |  | Likely Pathogenic (ClinVar) |
| 56334793 | c.529C>T (p.Arg177Ter) | rs2037724762 | *GNAO1A* | ENST00000262493 |  |  | 0.934 | D |  |  | 0.625005 | D |  |  | Conflicting interpretation (Pathogenic & Uncertain Significance, (ClinVar) ) |
| 56334794 | c.530G>C (p.Arg177Pro) | . | *GNAO1A* | ENST00000262493 | 1.0 | D | 0.968 | D | 0.943 | D | 0.493169 | D | 0.999681234359741 | D | Likely Pathogenic |
| 56334799 | c.535A>G (p.Arg179Gly) | . | *GNAO1A* | ENST00000262493 | 0.999 | D | 0.95 | D | 0.915 | D | 0.476341 | D | 0.998129665851593 | D | Pathogenic |
| 56334800 | c.536G>C (p.Arg179Thr) | . | *GNAO1A* | ENST00000262493 | 0.999 | D | 0.933 | D | 0.905 | D | 0.49191 | D | 0.999551117420197 | D | Likely Pathogenic (VarSome) |
| 56334809 | c.545C>T (p.Thr182Ile) | rs1596871452 | *GNAO1A* | ENST00000262493 | 1.0 | D | 0.977 | D | 0.989 | D | 0.558988 | D | 0.999319076538086 | D | Pathogenic |
| 56334814 | c.550G>A (p.Gly184Ser) | rs1555507383 | *GNAO1A* | ENST00000262493 | 0.988 | D | 0.928 | D | 0.954 | D | 0.545976 | D | 0.997089207172394 | D | Pathogenic***** |
| 56334814 | c.550G>C (p.Gly184Arg) | rs1555507383 | *GNAO1A* | ENST00000262493 | 1.0 | D | 0.98 | D | 0.972 | D | 0.577724 | D | 0.999771773815155 | D | Likely Pathogenic |
| 56334817 | c.553A>G (p.Ile185Val) | . | *GNAO1A* | ENST00000262493 | 0.968 | D | 0.438 | B | 0.668 | D | 0.172594 | D | 0.930847704410553 | D | Uncertain Significance (ClinVar) |
| 56334820 | c.556G>A (p.Val186Ile) | rs1331690209 | *GNAO1A* | ENST00000262493 | 0.825 | P | 0.313 | B | 0.444 | B | 0.108824 | D | 0.69301962852478 | D | Uncertain Significance |
| 56334836 | c.572_592 del (p.Thr191_Phe197 del) | rs587777056 | *GNAO1A* | ENST00000262493 |  |  |  |  |  |  |  |  |  |  | Pathogenic (ClinVar) |
| 56334845 | c.581A>C (p.Asn194Thr) | rs1596871479 | *GNAO1A* | ENST00000262493 | 0.003 | B | 0.543 | P | 0.457 | B | 0.0967774 | D | 0.91612035036087 | D | Uncertain Significance (VarSome) |
| 56334854 | c.590T>G (p.Phe197Cys) | rs2037725290 | *GNAO1A* | ENST00000262493 | 0.76 | P | 0.939 | D | 0.938 | D | 0.481252 | D | 0.997412621974945 | D | Uncertain Significance (VarSome) |
| 56336732 | c.595C>G (p.Leu199Val) | rs2037743524 | *GNAO1A* | ENST00000262493 | 0.765 | P | 0.51 | D | 0.632 | D | 0.129549 | D | 0.93217670917511 | D | Uncertain Significance (VarSome) |
| 56336733 | c.596T>C (p.Leu199Pro) | . | *GNAO1A* | ENST00000262493 | 1.0 | D | 0.967 | D | 0.970 | D | 0.493836 | D | 0.999198257923126 | D | Pathogenic (ClinVar) |
| 56336738 | c.601G>C (p.Asp201His) | rs567136805 | *GNAO1A* | ENST00000262493 | 1.0 | D | 0.98 | D | 0.967 | D | 0.565793 | D | 0.999572932720184 | D | Pathogenic (VarSome) |
| 56336739 | c.602A>T (p.Asp201Val) | . | *GNAO1A* | ENST00000262493 | 0.999 | D | 0.975 | D | 0.977 | D | 0.563685 | D | 0.999635457992554 | D | Pathogenic (ClinVar) |
| 56336741 | c.604G>C (p.Val202Leu) | rs1297388989 | *GNAO1A* | ENST00000262493 | 0.998 | D | 0.87 | D | 0.878 | D | 0.474763 | D | 0.995085299015045 | D | Likely Pathogenic (VarSome) |
| 56336741 | c.604G>A (p.Val202Ile) | rs1297388989 | *GNAO1A* | ENST00000262493 | 0.988 | B | 0.515 | D | 0.731 | D | 0.21699 | D | 0.996854364871979 | D | Likely Pathogenic (1)/ Uncertain Significance (2) (ClinVar) |
| 56336744 | c.607G>A (p.Gly203Arg) | rs587777057 | *GNAO1A* | ENST00000262493 | 1.0 | D | 0.903 | D | 0.941 | D | 0.544023 | D | 0.99994957447052 | D | Pathogenic (ClinVar) |
| 56336744 | c.607G>C (p.Gly203Arg) | rs587777057 | *GNAO1A* | ENST00000262494 | 1.0 | D | 0.903 | D | 0.941 | D | 0.544023 | D | 0.99994957447052 | D | Pathogenic (ClinVar) |
| 56336745 | c.608G>A (p.Gly203Glu) | . | *GNAO1A* | ENST00000262493 | 1.0 | D | 0.928 | D | 0.954 | D | 0.56581 | D | 0.999874114990234 | D | Pathogenic (ClinVar) |
| 56336747 | c.610G>C (p.Gly204Arg) | . | *GNAO1A* | ENST00000262493 | 1.0 | D | 0.856 | D | 0.931 | D | 0.560053 | D | 0.999893188476562 | D | Pathogenic (ClinVar) |
| 56336748 | c.611G>A (p.Gly204Asp) | . | *GNAO1A* | ENST00000262493 | 1.0 | D | 0.88 | D | 0.947 | D | 0.584647 | D | 0.999665379524231 | D | Likely Pathogenic |
| 56336751 | c.614A>C (p.Gln205Pro) | . | *GNAO1A* | ENST00000262493 | 1.0 | D | 0.909 | D | 0.970 | D | 0.470605 | D | 0.999668717384338 | D | Likely Pathogenic (ClinVar) |
| 56336753 | c.616C>T (p.Arg206Ter) | . | *GNAO1A* | ENST00000262493 |  |  | 0.959 | D |  |  | 0.625005 | D |  |  | Likely Pathogenic (VarSome) |
| 56336754 | c.617G>A (p.Arg206Gln) | rs1297225571 | *GNAO1A* | ENST00000262493 | 1.0 | D | 0.839 | D | 0.859 | D | 0.427062 | D | 0.999897241592407 | D | Pathogenic |
| 56336754 | c.617G>T (p.Arg206Leu) | . | *GNAO1A* | ENST00000262493 | 1.0 | D | 0.921 | D | 0.932 | D | 0.516341 | D | 0.999881029129028 | D | Pathogenic |
| 56336757 | c.620C>A (p.Ser207Tyr) | rs1057518440 | *GNAO1A* | ENST00000262493 | 1.0 | D | 0.908 | D | 0.913 | D | 0.429966 | D | 0.999233841896057 | D | Pathogenic (ClinVar) |
| 56336757 | c.620C>T (p.Ser207Phe) | . | *GNAO1A* | ENST00000262493 | 1.0 | D | 0.897 | D | 0.938 | D | 0.487747 | D | 0.999664187431335 | D | Pathogenic (ClinVar) |
| 56336759 | c.622G>C (p.Glu208Gln) | . | *GNAO1A* | ENST00000262493 | 1.0 | D | 0.811 | D | 0.780 | D | 0.326867 | D | 0.993200123310089 | D | Pathogenic* |
| 56336762 | c.625C>G (p.Arg209Gly) | rs886039494 | *GNAO1A* | ENST00000262493 | 1.0 | D | 0.943 | D | 0.924 | D | 0.495304 | D | 0.999528288841248 | D | Pathogenic (ClinVar) |
| 56336762 | c.625C>T (p.Arg209Cys) | rs886039494 | *GNAO1A* | ENST00000262493 | 1.0 | D | 0.947 | D | 0.888 | D | 0.503659 | D | 0.999947667121887 | D | Pathogenic (ClinVar) |
| 56336763 | c.626G>A (p.Arg209His) | rs797044878 | *GNAO1A* | ENST00000262493 | 1.0 | D | 0.926 | D | 0.914 | D | 0.528566 | D | 0.999944090843201 | D | Pathogenic (ClinVar) |
| 56336763 | c.626G>C (p.Arg209Pro) | . | *GNAO1A* | ENST00000262493 | 1.0 | D | 0.943 | D | 0.925 | D | 0.523226 | D | 0.999591529369354 | D | Pathogenic (ClinVar) |
| 56336763 | c.626G>T (p.Arg209Leu) | rs797044878 | *GNAO1A* | ENST00000262493 | 1.0 | D | 0.948 | D | 0.925 | D | 0.526013 | D | 0.999902486801147 | D | Pathogenic (ClinVar) |
| 56336781 | c.644G>A (p.Cys215Tyr) | . | *GNAO1A* | ENST00000262493 | 1.0 | D | 0.893 | D | 0.901 | D | 0.465205 | D | 0.999775230884552 | D | Pathogenic (ClinVar) |
| 56336786 | c.649G>A (p.Glu217Lys) | rs1555507477 | *GNAO1A* | ENST00000262493 | 0.905 | B | 0.894 | D | 0.841 | D | 0.453765 | D | 0.995627403259277 | D | Likely Pathogenic (ClinVar) |
| 56336788 | c.651G>C (p.Glu217Asp) | . | *GNAO1A* | ENST00000262493 | 0.885 | B | 0.687 | D | 0.740 | D | 0.180492 | D | 0.966401875019073 | D | Uncertain Significance (ClinVar) |
| 56336792 | c.655G>A (p.Val219Ile) | rs2037744218 | *GNAO1A* | ENST00000262493 | 0.999 | B | 0.487 | B | 0.658 | D | 0.194424 | D | 0.973159074783325 | D | Uncertain Significance (ClinVar) |
| 56336793 | c.656T>C (p.Val219Ala) | rs12721461 | *GNAO1A* | ENST00000262493 | 0.994 | D | 0.823 | D | 0.934 | D | 0.365745 | D | 0.997032642364502 | D | Likely Pathogenic (VarSome) |
| 56336799 | c.662C>A (p. Ala221Asp) | rs1555507479 | *GNAO1A* | ENST00000262493 | 1.0 | D | 0.934 | D | 0.951 | D | 0.486247 | D | 0.998945415019989 | D | Likely Pathogenic (ClinVar) |
| 56336811 | c.674G>A (p.Cys225Tyr) | . | *GNAO1A* | ENST00000262493 | 0.999 | D | 0.875 | D | 0.858 | D | 0.461052 | D | 0.999337375164032 | D | Uncertain Significance |
| 56336813 | c.676_677del (p.Val226ArgfsTer6) | rs1064797211 | *GNAO1A* | ENST00000262493 |  |  |  |  |  |  |  |  |  |  | Likely Pathogenic (ClinVar) |
| 56336817 | c.680C>T (p.Ala227Val) | rs797045599 | *GNAO1A* | ENST00000262493 | 1.0 | D | 0.96 | D | 0.885 | D | 0.457163 | D | 0.999571859836578 | D | Pathogenic (ClinVar) |
| 56336820 | c.683T>C (p.Leu228Pro) | rs1085307932 | *GNAO1A* | ENST00000262493 | 0.998 | D | 0.989 | D | 0.964 | D | 0.514756 | D | 0.999055325984955 | D | Likely Pathogenic (ClinVar) |
| 56336824 | c.687C>A (p.Ser229Arg) | rs546569747 | *GNAO1A* | ENST00000262493 | 1.0 | D | 0.972 | D | 0.883 | D | 0.456691 | D | 0.999123871326447 | D | Pathogenic** |
| 56336824 | c.687C>G (p.Ser229Arg) | rs546569747 | *GNAO1A* | ENST00000262493 | 1.0 | D | 0.972 | D | 0.883 | D | 0.456691 | D | 0.999123871326447 | D | Pathogenic** |
| 56336825 | c.688G>A (p.Gly230Ser) | rs2037744620 | *GNAO1A* | ENST00000262493 | 0.977 | D | 0.477 | B | 0.538 | D | 0.202278 | D | 0.888316512107849 | D | Uncertain Significance (VarSome) |
| 56336826 | c.689G>A (p.Gly230Asp) | rs759775512 | *GNAO1A* | ENST00000262493 | 0.941 | P | 0.381 | B | 0.527 | D | 0.299635 | D | 0.643134295940399 | D | Uncertain Significance (VarSome) |
| 56336829 | c.692A>G (p.Tyr231Cys) | rs1057518678 | *GNAO1A* | ENST00000262493 | 1.0 | D | 0.981 | D | 0.987 | D | 0.519862 | D | 0.999813258647919 | D | Pathogenic (ClinVar) |
| 56336835 | c.698A>C (p.Gln233Pro) | . | *GNAO1A* | ENST00000262493 | 0.995 | D | 0.87 | D | 0.889 | D | 0.463262 | D | 0.994879364967346 | D | Likely Pathogenic |
| 56336846 | c.709G>A (p.Glu237Lys) | rs1064794533 | *GNAO1A* | ENST00000262493 | 0.999 | D | 0.89 | D | 0.922 | D | 0.531399 | D | 0.999806106090546 | D | Pathogenic (ClinVar) |
| 56336855 | c.718A>G (p.Thr240Ala) | rs1163621468 | *GNAO1A* | ENST00000262493 | 0.017 | B | 0.414 | B | 0.373 | B | 0.0740018 | D | 0.789160847663879 | D | Uncertain Significance (VarSome) |
| 56336856 | c.719C>G (p.Thr240Ser) | rs2037744867 | *GNAO1A* | ENST00000262493 | 0.068 | B | 0.407 | B | 0.308 | B | 0.037732 | T | 0.745520114898682 | D | Uncertain Significance (VarSome) |
| 56336859 | c.722C>T (p.Thr241Met) | rs1456274473 | *GNAO1A* | ENST00000262493 | 1.0 | D | 0.244 | B | 0.475 | B | 0.0826503 | D | 0.887379050254822 | D | Uncertain Significance (VarSome) |
| 56336861 | c.723+1G>A | rs1596872804 | *GNAO1A* | ENST00000262493 |  |  |  |  |  |  |  |  |  |  | Pathogenic |
| 56336861 | c.723+1G>T | . | *GNAO1A* | ENST00000262493 |  |  |  |  |  |  |  |  |  |  | Likely Pathogenic |
| 56336862 | c.723+2T>A | . | *GNAO1A* | ENST00000262493 |  |  |  |  |  |  |  |  |  |  | Pathogenic |
| 56340835 | c.725A>T (p.Asn242Ile) | rs1470175907 | *GNAO1B* | ENST00000262494 | 1.0 | D | 0.908 | D | 0.874 | D | 0.360789 | D | 0.994233429431915 | D |  |
| 56340836 | c.726A>T (p.Asn242Ile) | rs1596875462 | *GNAO1B* | ENST00000262495 | 0.996 | D | 0.91 | D | 0.776 | D | 0.137446 | D | 0.988352596759796 | D |  |
| 56340837 | c.727C>G (p.Arg243Lys) | rs746569229 | *GNAO1B* | ENST00000262494 | 1.0 | D | 0.934 | D | 0.961 | D | 0.438329 | D | 0.968853175640106 | D |  |
| 56340837 | c.727C>T (p.Arg243Cys) | rs746569229 | *GNAO1B* | ENST00000262494 | 1.0 | D | 0.929 | D | 0.959 | D | 0.504739 | D | 0.999346673488617 | D |  |
| 56340838 | c.728G>A (p.Arg243His) | rs757172284 | *GNAO1B* | ENST00000262494 | 1.0 | D | 0.946 | D | 0.887 | D | 0.387339 | D | 0.974605977535248 | D |  |
| 56340840 | c.730A>C (p.Met244Leu) | rs1299928666 | *GNAO1B* | ENST00000262494 | 0.135 | B | 0.541 | D | 0.654 | D | 0.116045 | D | 0.751837491989136 | D |  |
| 56340840 | c.730A>T (p.Met244Leu) | rs1299928666 | *GNAO1B* | ENST00000262494 | 0.135 | B | 0.541 | D | 0.654 | D | -0.00169598 | T | 0.77945077419281 | D |  |
| 56340843 | c.733C>A (p.His245Asn) | rs990570885 | *GNAO1B* | ENST00000262494 | 0.67 | P | 0.814 | D | 0.716 | D | 0.159967 | D | 0.963886440212261 | D |  |
| 56340845 | c.735C>G (p.His245Gln) | rs200466903 | *GNAO1B* | ENST00000262494 | 0.039 | B | 0.826 | D | 0.560 | D | -0.021401 | T | 0.0297071246278927 | T |  |
| 56340846 | c.736G>A (p.Glu246Lys) | rs775322429 | *GNAO1B* | ENST00000262494 | 1.0 | D | 0.944 | D | 0.860 | D | 0.413681 | D | 0.984302878379822 | D |  |
| 56340849 | c.739T>G (p.Ser247Ala) | rs1212031214 | *GNAO1B* | ENST00000262494 | 0.63 | P | 0.833 | D | 0.799 | D | 0.299017 | D | 0.970712840557098 | D |  |
| 56340858 | c.748C>T (p.Leu250Phe) | rs749507624 | *GNAO1B* | ENST00000262494 | 1.0 | D | 0.799 | D | 0.962 | D | 0.497412 | D | 0.99429803446136 | D |  |
| 56340863 | c.753T>G (p.Phe251Leu) | rs2037795683 | *GNAO1B* | ENST00000262494 | 1.0 | D | 0.931 | D | 0.886 | D | 0.423508 | D | 0.992873430252075 | D |  |
| 56340864 | c.754G>A (p.Asp252Asn) | rs1337115692 | *GNAO1B* | ENST00000262494 | 0.859 | P | 0.872 | D | 0.532 | D | 0.0467008 | T | 0.951363916987514 | D |  |
| 56340868 | c.758G>T (p.Ser253Ile) | rs2037795767 | *GNAO1B* | ENST00000262494 | 0.999 | D | 0.922 | D | 0.891 | D | 0.45026 | D | 0.991268634796143 | D |  |
| 56340876 | c.766A>C (p.Asn256His) | rs967625844 | *GNAO1B* | ENST00000262494 | 1.0 | D | 0.918 | D | 0.885 | D | 0.206558 | D | 0.997682929039001 | D |  |
| 56340877 | c.767A>G (p.Asn256Ser) | rs1176989847 | *GNAO1B* | ENST00000262494 | 0.999 | D | 0.874 | D | 0.818 | D | 0.0808862 | D | 0.997077542853344 | D |  |
| 56340878 | c.768C>A (p.Asn257del) | . | *GNAO1A* | ENST00000262494 |  |  |  |  |  |  |  |  |  |  | Likely Benign (ClinVar) |
| 56340880 | c.770A>G (p.Asn257Ser) | rs143576848 | *GNAO1B* | ENST00000262494 | 0.616 | P | 0.533 | D | 0.322 | B | -0.140048 | T | 0.154049700825799 | T | Benign (ClinVar) |
| 56340898 | c.788C>A (p.Thr263Leu) | rs761819608 | *GNAO1B* | ENST00000262494 | 1.0 | D | 0.914 | D | 0.955 | D | 0.41791 | D | 0.995183527469635 | D |  |
| 56340898 | c.788C>T (p.Thr263Met) | rs761819608 | *GNAO1B* | ENST00000262494 | 1.0 | D | 0.878 | D | 0.940 | D | 0.400978 | D | 0.979250299280419 | D |  |
| 56340900 | c.790T>C (p.Ser264Pro) | rs1432592321 | *GNAO1B* | ENST00000262494 | 1.0 | D | 0.919 | D | 0.927 | D | 0.443614 | D | 0.996064007282257 | D |  |
| 56340903 | c.793A>G (p.Ile265Val) | rs202041900 | *GNAO1B* | ENST00000262494 | 0.0 | B | 0.259 | D | 0.289 | B | -0.0156115 | T | 0.112758427858353 | T |  |
| 56340906 | c.796A>G (p.Ile266Val) | rs1251314819 | *GNAO1B* | ENST00000262494 | 0.745 | P | 0.657 | D | 0.620 | D | 0.0496314 | T | 0.848723709583282 | D |  |
| 56340914-16 | c.805-806delCT p.Leu269Ter) | rs1202918689 | *GNAO1B* | ENST00000262494 |  |  |  |  |  |  |  |  |  |  |  |
| 56340921-23 | c.811-13delAAG (p.Lys271del) | rs1271044200 | *GNAO1B* | ENST00000262494 |  |  |  |  |  |  |  |  |  |  |  |
| 56340922 | c.812A>G (p.Lys271Arg) | rs1480620894 | *GNAO1B* | ENST00000262494 | 0.868 | P | 0.933 | D | 0.959 | D | 0.538649 | D | 0.97580486536026 | D |  |
| 56340925 | c.815A>C (p.Lys272Thr) | rs1455791075 | *GNAO1B* | ENST00000262494 | 0.999 | D | 0.827 | D | 0.884 | D | 0.310226 | D | 0.993369702887071 | D |  |
| 56340928 | c.818A>G (p.Asp273Gly) | rs2037796329 | *GNAO1B* | ENST00000262494 | 1.0 | D | 0.98 | D | 0.976 | D | 0.569656 | D | 0.995059609413147 | D |  |
| 56340928 | c.818A>T (p.Asp273Val) | rs2037796329 | *GNAO1B* | ENST00000262494 | 1.0 | D | 0.98 | D | 0.963 | D | 0.570459 | D | 0.997647225856781 | D |  |
| 56340929 | c.819C>G (p.Asp273Glu) | rs2037796359 | *GNAO1B* | ENST00000262494 | 0.994 | D | 0.932 | D | 0.952 | D | 0.530322 | D | 0.985615611076355 | D |  |
| 56340930 | c.820A>G (p.Ile274Val) | rs1190453161 | *GNAO1B* | ENST00000262494 | 0.003 | B | 0.411 | B | 0.390 | B | 0.0671285 | T | 0.579087257385254 | D |  |
| 56340931 | c.821T>C (p.Ile274Thr) | rs1365625924 | *GNAO1B* | ENST00000262494 | 0.07 | B | 0.728 | D | 0.800 | D | 0.148078 | D | 0.965037763118744 | D |  |
| 56340939 | c.829G>A (p.Glu277Lys) | rs754298120 | *GNAO1B* | ENST00000262494 | 0.008 | B | 0.359 | B | 0.554 | D | -0.031328 | T | 0.888582110404968 | D |  |
| 56340942 | c.832A>C (p.Lys278Gln) | rs1440336040 | *GNAO1B* | ENST00000262494 | 1.0 | D | 0.892 | D | 0.930 | D | 0.373466 | D | 0.995344250779496 | D |  |
| 56340943 | c.833A>G (p.Lys278Arg) | rs1596875553 | *GNAO1B* | ENST00000262494 | 1.0 | D | 0.866 | D | 0.939 | D | 0.359647 | D | 0.984969684499448 | D |  |
| 56340944 | c.834G>C (p.Lys278Asn) | rs758105660 | *GNAO1B* | ENST00000262494 | 1.0 | D | 0.903 | D | 0.859 | D | 0.120963 | D | 0.985375285148621 | D |  |
| 56340946 | c.836T>C (p.Ile279Thr) | rs1172828885 | *GNAO1B* | ENST00000262494 | 1.0 | D | 0.906 | D | 0.944 | D | 0.289293 | D | 0.993374288082123 | D |  |
| 56340947 | c.837C>G (p.Ile279Met) | rs1438245267 | *GNAO1B* | ENST00000262494 | 0.895 | P | 0.832 | D | 0.815 | D | 0.158133 | D | 0.957271099090576 | D |  |
| 56340958 | c.848C>T(p.Pro283Leu) | rs763864033 | *GNAO1B* | ENST00000262494 | 0.998 | D | 0.817 | D | 0.817 | D | 0.0600913 | T | 0.667436566002712 | D |  |
| 56340960 | c.850C>G (p.Leu284Val) | rs756726041 | *GNAO1B* | ENST00000262494 | 0.999 | D | 0.804 | D | 0.740 | D | 0.272684 | D | 0.965474903583527 | D |  |
| 56340960 | c.850C>T (p.Leu284phe) | rs756726041 | *GNAO1B* | ENST00000262494 | 1.0 | D | 0.877 | D | 0.782 | D | 0.384443 | D | 0.974032378795932 | D |  |
| 56340963 | c.853A>T (p.Thr285Ser) | rs781128298 | *GNAO1B* | ENST00000262494 | 0.0 | B | 0.233 | B | 0.264 | B | -0.0763194 | T | 0.268163675745789 | T |  |
| 56340964 | c.854C>T (p.Thr285Ile) | rs750314836 | *GNAO1B* | ENST00000262494 | 0.605 | P | 0.464 | B | 0.570 | D | 0.100598 | D | 0.929686725139618 | D |  |
| 56340966 | c.856A>G (p.Ile286Val) | rs1437835350 | *GNAO1B* | ENST00000262494 | 0.014 | B | 0.471 | B | 0.291 | B | 0.0870125 | D | 0.543049931526184 | D | Uncertain Significance |
| 56340970 | c.860G>C (p.Cys287Ser) | rs1274589060 | *GNAO1B* | ENST00000262494 | 0.999 | D | 0.902 | D | 0.850 | D | 0.448137 | D | 0.990301773641193 | D |  |
| 56340973 | c.863T>A (p.Phe288Tyr) | rs756144448 | *GNAO1B* | ENST00000262494 | 1.0 | D | 0.475 | B | 0.675 | D | -0.00734276 | T | 0.773921489715576 | D |  |
| 56340975 | c.865C>T (p.Pro289Ser) | rs908971585 | *GNAO1B* | ENST00000262494 | 0.435 | B | 0.827 | D | 0.780 | D | 0.260834 | D | 0.955481697738523 | D |  |
| 56340980 | c.870A>C (p.Glu290Asp) | rs779793623 | *GNAO1B* | ENST00000262494 | 0.036 | B | 0.333 | B | 0.214 | B | -0.146317 | T | 0.0885653644800186 | T |  |
| 56340981 | c.871T>C (p.Tyr291His) | rs1417942273 | *GNAO1B* | ENST00000262494 | 1.0 | D | 0.79 | D | 0.908 | D | 0.500219 | D | 0.99639904499054 | D |  |
| 56340982 | c.872A>G (p.Tyr291Cys) | rs749130075 | *GNAO1B* | ENST00000262494 | 1.0 | D | 0.923 | D | 0.962 | D | 0.436209 | D | 0.99628472328186 | D |  |
| 56340984 | c.874A>G (p.Thr292Ala) | rs1340835270 | *GNAO1B* | ENST00000262494 | 0.001 | B | 0.246 | B | 0.274 | B | -0.0903133 | T | 0.271214326416227 | T |  |
| 56340985 | c.875C>T (p.Thr292Ile) | rs2037797098 | *GNAO1B* | ENST00000262494 | 0.229 | B | 0.343 | B | 0.617 | D | 0.121885 | D | 0.934822861093052 | D |  |
| 56340987 | c.877+5A>G | . | *GNAO1B* | ENST00000262494 |  |  |  |  |  |  |  |  |  |  | Uncertain Significance |
| 56343763 | c.878G>A (p.Gly293Asp) | rs577181880 | *GNAO1B* | ENST00000262494 | 1.0 | D | 0.754 | D | 0.821 | D | 0.31995 | D | 0.986086237832204 | D |  |
| 56343763 | c.878G>T (p.Gly293Val) | rs577181880 | *GNAO1B* | ENST00000262494 | 1.0 | D | 0.804 | D | 0.893 | D | 0.357496 | D | 0.983075922118603 | D |  |
| 56343765 | c.880C>G (p.Pro294Ala) | rs770271324 | *GNAO1B* | ENST00000262494 | 0.0 | B | 0.124 | B | 0.278 | B | -0.0240057 | T | 0.131521748514488 | T |  |
| 56343765 | c.880C>T (p.Pro294Ser) | rs770271324 | *GNAO1B* | ENST00000262494 | 0.0 | B | 0.094 | B | 0.298 | B | -0.134348 | T | 0.125673173558201 | T |  |
| 56343771 | c.886G>A (p.Ala296Thr) | rs201386820 | *GNAO1B* | ENST00000262494 | 0.0 | B | 0.019 | B | 0.216 | B | -0.320584 | T | 0.00850739832862149 | T |  |
| 56343772 | c.887C>A (p.Ala296Asp) | rs1388376096 | *GNAO1B* | ENST00000262494 | 0.0 | B | 0.13 | B | 0.229 | B | -0.0889911 | T | 0.41854926943779 | T |  |
| 56343774 | c.889T>C (p.Phe297Leu) | rs952352700 | *GNAO1B* | ENST00000262494 | 0.034 | B | 0.451 | B | 0.528 | D | 0.0836312 | D | 0.918371027339697 | D |  |
| 56343775 | c.890T>C (p.Phe297Ser) | rs775989534 | *GNAO1B* | ENST00000262494 | 0.005 | B | 0.566 | D | 0.450 | B | -0.164059 | T | 0.294822992154129 | T |  |
| 56343777 | c.892A>G (p.Thr298Ala) | rs1340548576 | *GNAO1B* | ENST00000262494 | 0.002 | B | 0.066 | B | 0.196 | B | -0.144624 | T | 0.0503430358770442 | T |  |
| 56343778 | c.893C>T (p.Thr298Ile) | rs761341566 | *GNAO1B* | ENST00000262494 | 0.009 | B | 0.369 | B | 0.187 | B | -0.0551468 | T | 0.272588133811951 | T |  |
| 56343786 | c.901G>A (p.Val301Met) | rs539641021 | *GNAO1B* | ENST00000262494 | 0.303 | B | 0.358 | B | 0.421 | B | -0.103473 | T | 0.260463539348055 | T |  |
| 56343786 | c.901G>C (p.Val301Leu) | rs539641021 | *GNAO1B* | ENST00000262494 | 0.0 | B | 0.097 | B | 0.357 | B | 0.0173781 | T | 0.82055830152741 | D | Uncertain Significance (ClinVar) |
| 56343790 | c.905C>T (p.Ala302Val) | rs2037831573 | *GNAO1B* | ENST00000262494 | 0.066 | B | 0.371 | B | 0.288 | B | 0.016959 | T | 0.433938458597909 | T |  |
| 56343792 | c.907T>C (p.Tyr303His) | rs866235351 | *GNAO1B* | ENST00000262494 | 0.067 | B | 0.249 | B | 0.468 | B | 0.042324 | T | 0.620464146137238 | D |  |
| 56343798 | c.913C>G (p.Gln305Glu) | rs2037831757 | *GNAO1B* | ENST00000262494 | 0.474 | P | 0.41 | B | 0.459 | B | 0.0907677 | D | 0.484974950551987 | T |  |
| 56343801 | c.916G>T (p.Ala306Ser) | rs766236708 | *GNAO1B* | ENST00000262494 | 0.0 | B | 0.061 | B | 0.170 | B | -0.0846811 | T | 0.0565137553384562 | T |  |
| 56343802 | c.917C>A (p.Ala306Asp) | rs1433538642 | *GNAO1B* | ENST00000262494 | 0.001 | B | 0.348 | B | 0.288 | B | 0.0170983 | T | 0.152927900638326 | T |  |
| 56343802 | c.917C>T (p.Ala306Val) | rs1433538642 | *GNAO1B* | ENST00000262494 | 0.0 | B | 0.077 | B | 0.211 | B | -0.0381178 | T | 0.211057931184769 | T |  |
| 56343804 | c.919C>G (p.Gln307Glu) | rs1186990304 | *GNAO1B* | ENST00000262494 | 0.905 | P | 0.58 | D | 0.637 | D | 0.200686 | D | 0.813684932770519 | D |  |
| 56343805 | c.920A>G (p.Gln307Arg) | rs2037831972 | *GNAO1B* | ENST00000262494 | 0.27 | B | 0.535 | D | 0.623 | D | 0.288011 | D | 0.889395236968994 | D |  |
| 56343808 | c.923ins(T)10AATGATA (p.Tyr308fs) | rs1194554518 | *GNAO1B* | ENST00000262494 |  |  |  |  |  |  |  |  |  |  |  |
| 56343808 | c.923A>G(p.Tyr308Cys) | rs1235826058 | *GNAO1B* | ENST00000262494 | 0.932 | P | 0.686 | D | 0.814 | D | -0.035908 | T | 0.963667273521423 | D |  |
| 56343809 | c.924C>A (p.Tyr308Ter) | rs375972567 | *GNAO1B* | ENST00000262494 |  |  |  |  |  |  |  |  |  |  |  |
| 56343809 | c.924C>G (p.Tyr308Ter) | rs375972567 | *GNAO1B* | ENST00000262494 |  |  |  |  |  |  |  |  |  |  |  |
| 56343810 | c.925G>A (p.Glu309Lys) | rs139959591 | *GNAO1B* | ENST00000262494 | 1.0 | D | 0.322 | B | 0.749 | D | 0.0260619 | T | 0.476430526776622 | T |  |
| 56343811 | c.926-928delAGA (p.Glu309_Ser310delinsGly) | rs1477059431 | *GNAO1B* | ENST00000262494 |  |  |  |  |  |  |  |  |  |  |  |
| 56343812 | c.927insT(6) (p.Glu309insPhe(2)) | rs1188366062 | *GNAO1B* | ENST00000262494 |  |  |  |  |  |  |  |  |  |  |  |
| 56343813 | c.928A>G (p.Ser310Gly) | rs1850701123 | *GNAO1B* | ENST00000262494 | 0.065 | B | 0.13 | B | 0.405 | B | -0.0459734 | T | 0.732242226600647 | D |  |
| 56343814-15 | c.928-929delGC (p.Ser310fs) | rs1420071418 | *GNAO1B* | ENST00000262494 |  |  |  |  |  |  |  |  |  |  |  |
| 56343816-17 | c.930-931delAA (p.Lys311fs) | rs1409363778 | *GNAO1B* | ENST00000262494 |  |  |  |  |  |  |  |  |  |  |  |
| 56343816-27 | c.930-941delAAGAACAAGTCA (p.Lys311_Ser314del) | rs2037832412 | *GNAO1B* | ENST00000262494 |  |  |  |  |  |  |  |  |  |  |  |
| 56343817 | c.932A>G (p.Lys311Arg) | rs1168271775 | *GNAO1B* | ENST00000262494 | 0.001 | B | 0.074 | B | 0.313 | B | -0.0534829 | T | 0.465949487495974 | T |  |
| 56343817 | c.932A>T (p.Lys311Met) | rs1168271775 | *GNAO1B* | ENST00000262494 | 0.019 | B | 0.497 | B | 0.402 | B | 0.0910225 | D | 0.81635582447052 | D |  |
| 56343818 | c.933G>C (p.Lys311Asn) | rs537154549 | *GNAO1B* | ENST00000262494 | 0.863 | P | 0.402 | B | 0.590 | D | 0.00866624 | T | 0.887098429748985 | D |  |
| 56343819 | c.933insT (p.Asn312fs) | rs1383201691 | *GNAO1B* | ENST00000262494 |  |  |  |  |  |  |  |  |  |  |  |
| 56343820 | c.935A>G (p.Asn312Ser) | rs758503575 | *GNAO1B* | ENST00000262494 | 0.999 | D | 0.487 | B | 0.782 | D | -0.190198 | T | 0.701543748378754 | D |  |
| 56343820 | c.935A>T (p.Asn312Ile) | rs758503575 | *GNAO1B* | ENST00000262494 | 1.0 | D | 0.68 | D | 0.843 | D | 0.179132 | D | 0.993650257587433 | D |  |
| 56343820-29 | c.934-943delACAAGTCAGC (p.Asn312fs) | rs2037832718 | *GNAO1B* | ENST00000262494 |  |  |  |  |  |  |  |  |  |  |  |
| 56343822-23 | c.937-938delAA (p.Lys313fs) | rs1396467812 | *GNAO1B* | ENST00000262494 |  |  |  |  |  |  |  |  |  |  |  |
| 56343824 | c.939G>C (p.Lys313Asn) | rs2037832812 | *GNAO1B* | ENST00000262494 | 0.001 | B | 0.348 | B | 0.452 | B | 0.0494096 | T | 0.725518670101363 | D |  |
| 56343825 | c.940T>C ((p.Ser314Pro) | rs915713881 | *GNAO1B* | ENST00000262494 | 0.978 | D | 0.137 | B | 0.644 | D | 0.00235666 | T | 0.759205672085573 | D |  |
| 56343825-27 | c.940-943delTCA (p.Ser314del) | rs1461620146 | *GNAO1B* | ENST00000262494 |  |  |  |  |  |  |  |  |  |  |  |
| 56343829 | c.944insGA (p.Ala315fs) | rs1324750822 | *GNAO1B* | ENST00000262494 |  |  |  |  |  |  |  |  |  |  |  |
| 56343829 | c.944C>T (p.Ala315Val) | rs746997510 | *GNAO1B* | ENST00000262494 | 0.0 | B | 0.053 | B | 0.253 | B | -0.208083 | T | 0.255186229944229 | T |  |
| 56343832 | c.947A>C (p.His316Pro) | rs757426946 | *GNAO1B* | ENST00000262494 | 0.008 | B | 0.312 | B | 0.273 | B | 0.0046215 | T | 0.515899748760876 | D |  |
| 56343832 | c.947A>G (p.His316Arg) | rs757426946 | *GNAO1B* | ENST00000262494 | 0.002 | B | 0.201 | B | 0.283 | B | -0.166746 | T | 0.373046310255189 | T |  |
| 56343834-35 | c.949-950delAA (p.His316fs) | rs1355778249 | *GNAO1B* | ENST00000262494 |  |  |  |  |  |  |  |  |  |  |  |
| 56343835 | c.950A>C (p.Lys317Thr) | rs781482854 | *GNAO1B* | ENST00000262494 | 1.0 | D | 0.796 | D | 0.811 | D | 0.170138 | D | 0.994583308696747 | D |  |
| 56343835 | c.950A>G (p.Lys317Arg) | rs781482854 | *GNAO1B* | ENST00000262494 | 1.0 | D | 0.616 | D | 0.658 | D | 0.119115 | D | 0.864930868148804 | D |  |
| 56343852 | c.967G>A (p.Val323Ile) | rs770465304 | *GNAO1B* | ENST00000262494 | 0.001 | B | 0.035 | B | 0.134 | B | -0.335883 | T | 0.108068978678186 | T |  |
| 56343853-55 | c.989-970delTCA (p.Val323_Thr324delinsAla) | rs1567492756 | *GNAO1B* | ENST00000262494 |  |  |  |  |  |  |  |  |  |  |  |
| 56343861 | c.976G>A (p.Ala326Thr) | rs749685192 | *GNAO1B* | ENST00000262494 | 1.0 | D | 0.883 | D | 0.912 | D | 0.489871 | D | 0.986424619131761 | D |  |
| 56343861 | c.976G>T (p.Ala326Ser) | rs749685192 | *GNAO1B* | ENST00000262494 | 1.0 | D | 0.772 | D | 0.860 | D | 0.291177 | D | 0.934925996535696 | D |  |
| 56343864 | c.979A>G (p.Thr327Ala) | rs1276524211 | *GNAO1B* | ENST00000262494 | 0.999 | D | 0.781 | D | 0.937 | D | 0.400871 | D | 0.993110732935357 | D |  |
| 56343867 | c.982G>A (p.Asp328Asn) | rs191191827 | *GNAO1B* | ENST00000262494 | 1.0 | D | 0.863 | D | 0.887 | D | 0.153734 | D | 0.989143870365733 | D |  |
| 56343874 | c.989A>G (p.Asn330Ser) | rs1339460298 | *GNAO1B* | ENST00000262494 | 0.0 | B | 0.404 | B | 0.200 | B | 0.0285092 | T | 0.651443939582387 | D |  |
| 56343874 | c.989A>T (p.Asn330Ile) | rs1339460298 | *GNAO1B* | ENST00000262494 | 0.143 | B | 0.575 | D | 0.656 | D | 0.143009 | D | 0.936580048332696 | D |  |
| 56343877 | c.992A>G (p.Asn331Ser) | rs1222148370 | *GNAO1B* | ENST00000262494 | 1.0 | D | 0.74 | D | 0.800 | D | 0.0455556 | T | 0.99400546881642 | D |  |
| 56343879 | c.994A>G (p.Ile332Val) | rs2037834415 | *GNAO1B* | ENST00000262494 | 0.817 | P | 0.139 | B | 0.382 | D | -0.0138082 | T | 0.623290689913399 | D |  |
| 56343883 | c.998A>T (p.Gln333Leu) | rs776186191 | *GNAO1B* | ENST00000262494 | 0.958 | D | 0.617 | D | 0.862 | D | 0.283765 | D | 0.984543979167938 | D |  |
| 56343889 | c.1004T>C (p.Val335Ala) | rs759435322 | *GNAO1B* | ENST00000262494 | 1.0 | D | 0.816 | D | 0.963 | D | 0.461322 | D | 0.986497700214386 | D |  |
| 56343892 | c.1007T>C (p.Phe336Ser) | rs2037834621 | *GNAO1B* | ENST00000262494 | 1.0 | D | 0.904 | D | 0.962 | D | 0.48689 | D | 0.99760901927948 | D |  |
| 56343893 | c.1008T>G (p.Phe336Leu) | rs903948500 | *GNAO1B* | ENST00000262494 | 1.0 | D | 0.778 | D | 0.811 | D | 0.349469 | D | 0.972331609192834 | D |  |
| 56343894 | c.1009G>A (p.Asp337Asn) | rs765246895 | *GNAO1B* | ENST00000262494 | 0.061 | B | 0.702 | D | 0.448 | B | 0.0238455 | T | 0.808066964149475 | D |  |
| 56343896 | c.1011T>G (p.Asp337Glu) | rs1347798085 | *GNAO1B* | ENST00000262494 | 0.529 | P | 0.704 | D | 0.527 | D | 0.0949649 | D | 0.348080734303049 | T |  |
| 56343898 | c.1013C>T (p.Ala338Val) | rs1429909588 | *GNAO1B* | ENST00000262494 | 1.0 | D | 0.649 | D | 0.750 | D | 0.285885 | D | 0.940721724048469 | D |  |
| 56343901 | c.1016T>C (p.Val339Ala) | rs1307303367 | *GNAO1B* | ENST00000262494 | 1.0 | D | 0.783 | D | 0.927 | D | 0.308246 | D | 0.977803349494934 | D |  |
| 56343904 | c.1019C>T (p.Thr340Met) | rs200947037 | *GNAO1B* | ENST00000262494 | 1.0 | D | 0.675 | D | 0.874 | D | 0.12055 | D | 0.620256283033393 | D |  |
| 56343909 | c.1024G>A (p.Val342Ile) | rs1330301776 | *GNAO1B* | ENST00000262494 | 0.001 | B | 0.308 | B | 0.274 | B | 0.102572 | D | 0.737619495637641 | D |  |
| 56343909 | c.1024G>T (p.Val342Phe) | rs1330301776 | *GNAO1B* | ENST00000262494 | 0.935 | P | 0.79 | D | 0.844 | D | 0.130811 | D | 0.966668248176575 | D |  |
| 56343915 | c.1030A>G (p.Ile344Val) | rs757621484 | *GNAO1B* | ENST00000262494 | 0.893 | P | 0.421 | B | 0.667 | D | 0.13046 | D | 0.796938183112058 | D |  |
| 56343918 | c.1033G>A (p.Ala345Thr) | rs201789251 | *GNAO1B* | ENST00000262494 | 0.995 | D | 0.489 | B | 0.408 | B | -0.0781232 | T | 0.300860941410065 | T |  |
| 56343925 | c.1040A>C (p.Asn330Thr) | rs1596877596 | *GNAO1B* | ENST00000262494 | 0.145 | B | 0.782 | D | 0.704 | D | 0.107979 | D | 0.891397833824158 | D |  |
| 56343930 | c.1045C>T (p.Arg349Trp) | rs756529307 | *GNAO1B* | ENST00000262494 | 1.0 | D | 0.436 | B | 0.710 | D | -0.0272173 | T | 0.980510771274567 | D | Likely Benign* |
| 56343931 | c.1046G>A(p.Arg349Gln) | rs780642028 | *GNAO1B* | ENST00000262494 | 0.985 | D | 0.346 | B | 0.576 | D | -0.0544025 | T | 0.46229616106276 | T |  |
| 56343937 | c.1052G>A (p.Cys351Tyr) | rs769077831 | *GNAO1B* | ENST00000262494 | 1.0 | D | 0.75 | D | 0.784 | D | 0.390389 | D | 0.98968517780304 | D |  |
| 56343938 | c.1053T>G (p.Cys351Trp) | rs1596877620 | *GNAO1B* | ENST00000262494 | 1.0 | D | 0.761 | D | 0.736 | D | 0.31701 | D | 0.992941379547119 | D |  |
| 56343946 | c.1061A>G (p.Tyr354Cys) | rs1479387344 | *GNAO1B* | ENST00000262494 | 1.0 | D | 0.517 | D | 0.768 | D | 0.19684 | D | 0.99583625793457 | D |  |
| 56351376 | c.724-8G>A (p.Thr241_Asn-242insProGln) | rs1085307876 | *GNAO1A* | ENST00000262493 |  |  |  |  |  |  |  |  |  |  | Pathogenic |
| 56351385 | c.725A>C (p.Asn242Thr) | rs1085307894 | *GNAO1A* | ENST00000262493 | 1.0 | D | 0.912 | D | 0.902 | D | 0.259001 | D | 0.994220018386841 | D | Pathogenic (ClinVar) |
| 56351390 | c.730_731delATinsCG (p.Met244Arg) | . | *GNAO1A* | ENST00000262493 |  |  |  |  |  |  |  |  |  |  |  |
| 56351391 | c.731T>A (p.Met244Lys) | rs2037919953 | *GNAO1A* | ENST00000262493 | 1.0 | D | 0.959 | D | 0.951 | D | 0.492163 | D | 0.998570322990417 | D | Likely Pathogenic (ClinVar) |
| 56351394 | c.734A>G (p.His245Arg) | rs760862770 | *GNAO1A* | ENST00000262493 | 0.628 | P | 0.778 | D | 0.714 | D | 0.356508 | D | 0.578723112320771 | D | Uncertain Significance (VarSome) |
| 56351396 | c.736G>A (p.Glu246Lys) | rs797044951 | *GNAO1A* | ENST00000262493 | 1.0 | D | 0.934 | D | 0.844 | D | 0.497423 | D | 0.999531865119934 | D | Pathogenic |
| 56351396 | c.736G>C (p.Glu246Gln) | rs797044951 | *GNAO1A* | ENST00000262493 | 1.0 | D | 0.934 | D | 0.775 | D | 0.448716 | D | 0.998391032218933 | D | Pathogenic |
| 56351397 | c.737A>G (p.Glu246Gly) | rs1114167431 | *GNAO1A* | ENST00000262493 | 1.0 | D | 0.936 | D | 0.931 | D | 0.501147 | D | 0.998062074184418 | D | Pathogenic |
| 56351397 | c.737A>T (p.Glu246Val) | . | *GNAO1A* | ENST00000262493 | 1.0 | D | 0.966 | D | 0.950 | D | 0.497482 | D | 0.992054462432861 | D | Pathogenic |
| 56351406 | c.746T>C (p.Met249Thr) | rs1357923280 | *GNAO1A* | ENST00000262493 | 0.017 | B | 0.701 | D | 0.387 | B | 0.333029 | D | 0.482303434909819 | T | Uncertain Significance (ClinVar) |
| 56351408 | c.748C>T (p.Leu250Phe) | . | *GNAO1A* | ENST00000262493 | 1.0 | D | 0.862 | D | 0.910 | D | 0.367048 | D | 0.99947053194046 | D | Pathogenic (ClinVar) |
| 56351413 | c.753C>G (p.Phe251Leu) | rs755484788 | *GNAO1A* | ENST00000262493 | 1.0 | D | 0.956 | D | 0.921 | D | 0.470975 | D | 0.999056756496429 | D |  |
| 56351414 | c.754G>A (p.Asp252Asn) | rs2037920339 | *GNAO1A* | ENST00000262493 | 0.977 | D | 0.311 | B | 0.599 | D | -0.0041043 | T | 0.976616680622101 | D | Likely Benign (ClinVar) |
| 56351417 | c.759dup (p.Ile254HisfsTer3) | rs2037920369 | *GNAO1A* | ENST00000262493 |  |  |  |  |  |  |  |  |  |  | Uncertain Significance (ClinVar) |
| 56351425 | c.765dupT (p.Asn256Ter) | . | *GNAO1A* | ENST00000262494 |  |  |  |  |  |  |  |  |  |  | Likely Pathogenic |
| 56351431 | c.771_773delAAC (p.Asn256del) | [rs1567496374](https://www.ncbi.nlm.nih.gov/snp/rs1567496374) | *GNAO1A* | ENST00000262493 |  |  |  |  |  |  |  |  |  |  | Likely Benign (ClinVar) |
| 56351450 | c.790T>C (p.Ser264Pro) | rs112085622 | *GNAO1A* | ENST00000262493 | 1.0 | D | 0.875 | D | 0.911 | D | 0.440862 | D | 0.992855906486511 | D |  |
| 56351453 | c.793A>G (p.Ile265Val) | rs2037920534 | *GNAO1A* | ENST00000262493 | 0.0 | B | 0.485 | B | 0.293 | B | 0.0586345 | T | 0.907596170902252 | D | Likely Benign (ClinVar) |
| 56351468 | c.808A>C (p.Asn270His) | . | *GNAO1A* | ENST00000262493 | 1.0 | D | 0.939 | D | 0.974 | D | 0.337898 | D | 0.996622443199158 | D | Pathogenic |
| 56351468 | c.808A>G (p.Asn270Asp) | . | *GNAO1A* | ENST00000262493 | 1.0 | D | 0.969 | D | 0.939 | D | 0.258656 | D | 0.988047957420349 | D | Likely Pathogenic* |
| 56351469 | c.809A>C (p.Asn270Thr) | rs1596881854 | *GNAO1A* | ENST00000262493 | 1.0 | D | 0.946 | D | 0.928 | D | 0.341571 | D | 0.992316603660583 | D | Uncertain Significance (ClinVar), ***Likely Pathogenic |
| 56351470 | c.810C>A (p.Asn270Lys) | . | *GNAO1A* | ENST00000262493 | 1.0 | D | 0.908 | D | 0.849 | D | 0.264066 | D | 0.999241471290588 | D | Likely Pathogenic |
| 56351471 | c.811A>G (p.Lys271Glu) | rs1555508311 | *GNAO1A* | ENST00000262493 | 0.999 | D | 0.945 | D | 0.979 | D | 0.563077 | D | 0.994622349739075 | D | Uncertain Significance (ClinVar), Likely Pathogenic (ClinVar) |
| 56351473 | c.813G>C (p.Lys271Asn) | rs758779535 | *GNAO1A* | ENST00000262493 | 1.0 | D | 0.92 | D | 0.791 | D | 0.414358 | D | 0.998906254768372 | D | Likely Pathogenic (ClinVar) |
| 56351477 | c.817G>T (p.Asp273Tyr) | . | *GNAO1A* | ENST00000262493 | 1.0 | D | 0.974 | D | 0.913 | D | 0.584744 | D | 0.999496221542358 | D | Likely Pathogenic (Decipher) |
| 56351478 | c.818A>T (p.Asp273Val) | rs2037920694 | *GNAO1A* | ENST00000262493 | 1.0 | D | 0.982 | D | 0.975 | D | 0.583281 | D | 0.997957706451416 | D | Uncertain Significance ***Likely Pathogenic |
| 56351484 | c.824T>C (p.Phe275Ser) | . | *GNAO1A* | ENST00000262493 | 1.0 | D | 0.946 | D | 0.980 | D | 0.520521 | D | 0.995314121246338 | D | Pathogenic |
| 56351492 | c.833_835del (p.Lys278del) | rs2037920791 | *GNAO1A* | ENST00000262493 |  |  |  |  |  |  |  |  |  |  | Pathogenic* |
| 56351496 | c.836T>A (p.Ile279Asn) | rs587777054 | *GNAO1A* | ENST00000262493 | 1.0 | D | 0.95 | D | 0.931 | D | 0.341576 | D | 0.999458372592926 | D | Pathogenic |
| 56351504 | c.844T>C (p.Ser282Pro) | . | *GNAO1A* | ENST00000262493 | 1.0 | D | 0.66 | D | 0.870 | D | 0.416502 | D | 0.994602382183075 | D | Likely Pathogenic (ClinVar)* |
| 56351507 | c.847C>T (p.Pro283Ser) | rs779593919 | *GNAO1A* | ENST00000262493 | 0.824 | B | 0.422 | B | 0.411 | B | 0.114808 | D | 0.683395266532898 | D | Uncertain Significance (VarSome) |
| 56351511 | c.851T>C (p.Leu284Ser) | rs1555508316 | *GNAO1A* | ENST00000262493 | 1.0 | D | 0.963 | D | 0.949 | D | 0.490295 | D | 0.992056906223297 | D | Pathogenic (ClinVar, Ensembl) |
| 56351516 | c.856A>G (p.Ile286Val) | rs1386785579 | *GNAO1A* | ENST00000262493 | 0.017 | B | 0.514 | D | 0.277 | B | 0.113 | D | 0.410446325115391 | T | Uncertain Significance (Ensembl) |
| 56351516 | c.856A>T (p.Ile286Phe) | rs1386785579 | *GNAO1A* | ENST00000262493 | 0.979 | D | 0.682 | D | 0.695 | D | 0.217066 | D | 0.966084480285645 | D | Uncertain Significance (ClinVar) |
| 56351523 | c.863T>C (p.Phe288Ser) | . | *GNAO1A* | ENST00000262493 | 1.0 | D | 0.952 | D | 0.953 | D | 0.497795 | D | 0.998897671699524 | D | Uncertain Significance (ClinVar) |
| 56351525 | c.865C>G (p.Gly289Pro) | rs768674142 | *GNAO1A* | ENST00000262493 | 0.028 | B | 0.803 | D | 0.685 | D | 0.197028 | D | 0.622785754746523 | D | Uncertain Significance (VarSome) |
| 56351531 | c.871T>A (p.Tyr291Asn) | rs1064795384 | *GNAO1A* | ENST00000262493 | 1.0 | D | 0.959 | D | 0.941 | D | 0.479602 | D | 0.999166131019592 | D | Pathogenic (ClinVar) |
| 56351532 | c.872A>G (p.Tyr291Cys) | . | *GNAO1A* | ENST00000262493 | 1.0 | D | 0.89 | D | 0.939 | D | 0.423035 | D | 0.999013185501099 | D | Likely Pathogenic (VarSome) |
| 56351535 | c.875C>T (p.Tyr292Thr) | rs2037921244 | *GNAO1A* | ENST00000262493 | 0.774 | P | 0.224 | B | 0.613 | D | 0.119108 | D | 0.973941087722778 | D | Uncertain Significance (VarSome) |
| 56351539 | c.877+2T>A | rs1002222317 | *GNAO1A* | ENST00000262493 |  |  |  |  |  |  |  |  |  |  | Likely benign and predicted to result in Thr292insGly(2)**** |
| 56354868 | c.880C>T (p.Pro294Ser) | rs1219437188 | *GNAO1A* | ENST00000262493 | 0.0 | B | 0.09 | B | 0.246 | B | -0.0977545 | T | 0.113053031265736 | T | Uncertain Significance (VarSome) |
| 56354872 | c.884A>G (p.Asn295Ser) | rs371362351 | *GNAO1A* | ENST00000262493 | 0.0 | B | 0.614 | D | 0.405 | B | -0.0511563 | T | 0.528432607650757 | D | Uncertain Significance (ClinVar) |
| 56354874 | c.886A>C (p.Thr296Pro) | rs754547263 | *GNAO1A* | ENST00000262493 | 0.174 | B | 0.748 | D | 0.686 | D | 0.219382 | D | 0.926612794399261 | D | Uncertain Significance (VarSome) |
| 56354875 | c.887C>T (p.Thr296Ile) | rs1596883621 | *GNAO1A* | ENST00000262493 | 0.083 | B | 0.62 | D | 0.668 | D | 0.278085 | D | 0.939460039138794 | D | Uncertain Significance (VarSome) |
| 56354882 | c.894A>T (p.Glu298Asp) | rs2037953814 | *GNAO1A* | ENST00000262493 | 0.0 | B | 0.144 | B | 0.144 | B | -0.11757 | T | 0.103585769005483 | T | Uncertain Significance (ClinVar) but prediction points toward Likely Benign*** |
| 56354885 | c.897insAAG (p.Lys300ins) | rs2037953880 | *GNAO1A* | ENST00000262493 |  |  |  |  |  |  |  |  |  |  |  |
| 56354886 | c.898G>A (p.Ala300Thr) | rs748093621 | *GNAO1A* | ENST00000262493 | 0.997 | D | 0.768 | D | 0.741 | D | 0.305109 | D | 0.95949250459671 | D | Likely Pathogenic (VarSome) |
| 56354889 | c.901_903del (p.Ala301del) | . | *GNAO1A* | ENST00000262493 |  |  |  |  |  |  |  |  |  |  | Likely Pathogenic |
| 56354892 | c.904G>A (p.Ala302Thr) | . | *GNAO1A* | ENST00000262493 | 0.01 | B | 0.47 | B | 0.347 | B | 0.18819 | D | 0.946539640426636 | D | Likely benign (ClinVar) |
| 56354905 | c.917C>A (p.Ala306Glu) | rs2037953966 | *GNAO1A* | ENST00000262493 | 0.045 | B | 0.463 | B | 0.351 | B | 0.0561165 | T | 0.372051785019247 | T | Uncertain Significance (VarSome) |
| 56354912 | c.924T>G (p.Phe308Leu) | rs57295392 | *GNAO1A* | ENST00000262493 | 0.568 | P | 0.803 | D | 0.820 | D | 0.36386 | D | 0.980157017707825 | D | Likely Pathogenic (VarSome) |
| 56354917 | c.929G>A (p.Ser310Asn) | rs771141469 | *GNAO1A* | ENST00000262493 | 0.0 | B | 0.162 | B | 0.246 | B | -0.0731644 | T | 0.873944520950317 | D | Uncertain Significance (VarSome) |
| 56354925 | c.937C>T (p.Arg313Cys) | . | *GNAO1A* | ENST00000262493 | 0.956 | P | 0.691 | D | 0.706 | D | 0.384401 | D | 0.996482014656067 | D | Likely Pathogenic (VarSome) |
| 56354926 | c.938G>A (p.Arg313His) | . | *GNAO1A* | ENST00000262493 | 0.442 | B | 0.622 | D | 0.670 | D | 0.278699 | D | 0.969395399093628 | D | Uncertain Significance (ClinVar) |
| 56354935 | c.947A>G (p.Asn316Ser) | rs1191986177 | *GNAO1A* | ENST00000262493 | 0.0 | B | 0.465 | B | 0.233 | B | -0.0725726 | T | 0.455139458179474 | T | Uncertain Significance (VarSome) |
| 56354955 | c.967A>G (p.Met323Val) | rs1383543515 | *GNAO1A* | ENST00000262493 | 0.0 | B | 0.627 | D | 0.421 | B | 0.115339 | D | 0.525909125804901 | D | Uncertain Significance (VarSome) |
| 56354961 | c.973T>G (p.Cys325Gly) | rs2037954227 | *GNAO1A* | ENST00000262493 | 1.0 | D | 0.887 | D | 0.930 | D | 0.449467 | D | 0.999951004981995 | D | Likely Pathogenic (ClinVar) |
| 56354967 | c.979A>C (p.Thr327Pro) | rs1470946386 | *GNAO1A* | ENST00000262493 | 1.0 | D | 0.693 | D | 0.948 | D | 0.496887 | D | 0.99830174446106 | D | Likely Pathogenic |
| 56354968 | c.980C>A (p.Thr327Lys) | . | *GNAO1A* | ENST00000262493 | 1.0 | D | 0.929 | D | 0.930 | D | 0.493439 | D | 0.999653339385986 | D | Likely Pathogenic |
| 56354968 | c.980C>G (p.Thr327Arg) | . | *GNAO1A* | ENST00000262493 | 1.0 | D | 0.937 | D | 0.944 | D | 0.489806 | D | 0.998536705970764 | D | Likely Pathogenic |
| 56354971 | c.983A>G (p.Asp328Gly) | rs878853051 | *GNAO1A* | ENST00000262493 | 1.0 | D | 0.941 | D | 0.949 | D | 0.514684 | D | 0.999679327011108 | D | Likely Pathogenic (ClinVar, Ensembl) |
| 56354973 | c.986del (p.Thr329Argfs*45) | . | *GNAO1A* | ENST00000262493 |  |  |  |  |  |  |  |  |  |  | Uncertain Significance |
| 56354988 | c.1000G>A (p.Val334Met) | rs2037954431 | *GNAO1A* | ENST00000262493 | 0.1 | B | 0.574 | D | 0.603 | D | 0.199454 | D | 0.821047186851501 | D | Uncertain Significance (VarSome) |
| 56355001 | c.1013_1015del (p.Ala338del) | . | *GNAO1A* | ENST00000262493 |  |  |  |  |  |  |  |  |  |  | Likely Pathogenic |
| 56355003 | c.1015G>A (p.Val339Ile) | rs2037954591 | *GNAO1A* | ENST00000262493 | 1.0 | D | 0.522 | D | 0.603 | D | 0.162103 | D | 0.950883626937866 | D | Uncertain Significance (ClinVar) |
| 56355018 | c.1030_1032delATT (p.Ile344del) | rs2037954707 | *GNAO1A* | ENST00000262493 |  |  |  |  |  |  |  |  |  |  | Pathogenic***/  Likely Pathogenic (ClinVar) |
| 56355019 | c.1031T>C (p.Ile344Thr) | . | *GNAO1A* | ENST00000262493 | 0.999 | D | 0.904 | D | 0.930 | D | 0.431879 | D | 0.997752010822296 | D | Uncertain Significance (VarSome) |
| 56355025 | c.1031A>T (p.Asn346Ile) | rs372737966 | *GNAO1A* | ENST00000262493 | 0.173 | B | 0.803 | D | 0.685 | D | 0.256486 | D | 0.977602064609528 | D | Uncertain Significance (VarSome) |
| 56355034 | c.1046_1055del10ins10(p.R349_G352delinsQGCA) | . | *GNAO1A* | ENST00000262493 |  |  |  |  |  |  |  |  |  |  | Uncertain Significance |
| 56355037 | c.1049G>A (p.Gly350Asp) | rs2037954783 | *GNAO1A* | ENST00000262493 | 0.986 | D | 0.658 | D | 0.480 | B | 0.235946 | D | 0.930165469646454 | D | Uncertain Significance (VarSome) |
| 56355042 | c.1054G>A (p.Gly352Ser) | . | *GNAO1A* | ENST00000262493 | 1.0 | D | 0.551 | D | 0.777 | D | 0.170127 | D | 0.985356213598028 | D | Uncertain Significance (VarSome) |

* (Likely) pathogenic on basis of an *in vitro* study (Slepak et al., 1993)

** Pathogenic on basis of our unpublished functional data

*** Pathogenic on basis of multiple NDD patients with the same variant

**** Likely pathogenic on basis of multiple NDD patients with similar variants

***** Pathogenic on basis of Kehrl et al. (Kehrl et al., 2014)

**Table S2. Overview of (likely) pathogenic *GNAO1* variants.**

| **CHR16**  **POSITION** | **VARIANT** | **rs_dbSNP** | **GENE** | **ENSEMBL_TRANSCRIPT ID** | **PolyPhen2_**  **HDIV** | **PREDICTION** | **VEST4** | **PREDICTION** | **REVEL** | **PREDICTION** | **BayesDel_**  **addAF** | **PREDICTION** | **ClinPred** | **PREDICTION** | **ACMG CLASSIFICATION** | **REFERENCE** |
| --- | --- | --- | --- | --- | --- | --- | --- | --- | --- | --- | --- | --- | --- | --- | --- | --- |
| 56192273 | c.38T>C (p.Leu13Pro) | rs1555499768 | *GNAO1A* | ENST00000262493 | 0.007 | B | 0.337 | B | 0.385 | B | 0.0979789 | D | 0.805835008621216 | D | Likely Pathogenic (ClinVar, Ensembl) |  |
| 56192293 | c.58G>A (p.Glu20Lys) | rs2036182701 | *GNAO1A* | ENST00000262493 | 0.779 | B | 0.443 | B | 0.842 | D | 0.297882 | D | 0.985042154788971 | D | Likely Pathogenic, Uncertain Significance (ClinVar) |  |
| 56192303 | c.68T > C (p.Leu23Pro) | . | *GNAO1A* | ENST00000262493 | 1.0 | D | 0.867 | D | 0.973 | D | 0.475924 | D | 0.999778807163239 | D | Likely Pathogenic | (Wirth et al., 2022) |
| 56192346 | c.111_113delCCT (p.Leu39del) | . | *GNAO1A* | ENST00000262493 |  |  |  |  |  |  |  |  |  |  | Likely Pathogenic (Decipher) | (Deciphering Developmental Disorders, 2017) |
| 56192351 | c.116T>C (p.Leu39Pro) | rs1555499769 | *GNAO1A* | ENST00000262493 | 1.0 | D | 0.919 | D | 0.971 | D | 0.534903 | D | 0.99897837638855 | D | Likely Pathogenic, Uncertain Significance (ClinVar, Ensembl) |  |
| 56192353 | c.118G>A (p.Gly40Arg) | rs886041715 | *GNAO1A* | ENST00000262493 | 1.0 | D | 0.947 | D | 0.983 | D | 0.583003 | D | 0.999903678894043 | D | Pathogenic | (Lecoquierre F., 2018) |
| 56192353 | c.118G>C (p.Gly40Arg) | rs886041715 | *GNAO1A* | ENST00000262493 | 1.0 | D | 0.947 | D | 0.983 | D | 0.583003 | D | 0.999881267547607 | D | Pathogenic | (Deciphering Developmental Disorders, 2017), this study |
| 56192353 | c.118G>T (p.Gly40Trp) | rs886041715 | *GNAO1A* | ENST00000262493 | 1.0 | D | 0.878 | D | 0.978 | D | 0.583003 | D | 0.99984085559845 | D | Pathogenic | (Kelly et al., 2019) |
| 56192574 | c.119G>A (p.Gly40Glu) | rs886041766 | *GNAO1A* | ENST00000262493 | 1.0 | D | 0.949 | D | 0.996 | D | 0.584152 | D | 0.999758899211884 | D | Pathogenic (ClinVar) | (Kelly et al., 2019) |
| 56192574 | c.119G>T (p.Gly40Val) | rs886041766 | *GNAO1A* | ENST00000262493 | 1.0 | D | 0.948 | D | 0.995 | D | 0.584285 | D | 0.999819576740265 | D | Likely Pathogenic (ClinVar) |  |
| 56192579 | c.124G​>C (p.Gly42Arg) | . | *GNAO1A* | ENST00000262493 | 1.0 | D | 0.623 | D | 0.955 | D | 0.423286 | D | 0.9992795586586 | D | Pathogenic | (Zhu et al., 2015) |
| 56192579 | c.124G>A (p.Gly42Arg) | . | *GNAO1A* | ENST00000262493 | 1.0 | D | 0.623 | D | 0.955 | D | 0.423286 | D | 0.999132335186005 | D | Pathogenic | (Y. Liu et al., 2022) |
| 56192588 | c.133G>C (p.Gly45Arg) | rs869312939 | *GNAO1A* | ENST00000262493 | 1.0 | D | 0.954 | D | 0.985 | D | 0.58415 | D | 0.999623537063599 | D | Pathogenic (ClinVar) | (Powis et al., 2020)=(K. L. Helbig et al., 2016) |
| 56192589 | c.134G>A (p.Gly45Glu) | . | *GNAO1A* | ENST00000262493 | 1.0 | D | 0.969 | D | 0.980 | D | 0.584148 | D | 0.999809324741364 | D | Pathogenic | (Gawlinski et al., 2016) |
| 56192591 | c.136A>G (p.Lys46Glu) | . | *GNAO1A* | ENST00000262493 | 1.0 | D | 0.819 | D | 0.963 | D | 0.578381 | D | 0.99932587146759 | D | Pathogenic *** (multiple) | (Yang et al., 2021). |
| 56192592 | c.137A>G (p.Lys46Arg) | . | *GNAO1A* | ENST00000262493 | 1.0 | D | 0.72 | D | 0.948 | D | 0.548404 | D | 0.999465882778168 | D | Likely Pathogenic | (Wirth et al., 2022) |
| 56192593 | c.138A>T (p.Lys46Asn) | . | *GNAO1A* | ENST00000262493 | 1.0 | D | 0.805 | D | 0.898 | D | 0.3585 | D | 0.999612152576447 | D | Likely Pathogenic (ClinVar) | (Thiel et al., 2023) |
| 56192594 | c.139A>G (p.Ser47Gly) | . | *GNAO1A* | ENST00000262493 | 1.0 | D | 0.795 | D | 0.950 | D | 0.484377 | D | 0.999099254608154 | D | Pathogenic*** | (Danti et al., 2017), this study |
| 56192595 | c.140G>A (p.Ser47Asn) | rs1596787821 | *GNAO1A* | ENST00000262493 | 0.999 | D | 0.807 | D | 0.855 | D | 0.222452 | D | 0.999606072902679 | D | Pathogenic (ClinVar) |  |
| 56192596 | c.141C>A (p.Ser47Arg) | . | *GNAO1A* | ENST00000262493 | 1.0 | D | 0.946 | D | 0.914 | D | 0.470333 | D | 0.999834179878235 | D | Likely pathogenic | this study |
| 56192598 | c.143C>A (p.Thr48Asn) | rs1555499800 | *GNAO1A* | ENST00000262493 | 1.0 | D | 0.903 | D | 0.888 | D | 0.220326 | D | 0.999808013439178 | D | Likely Pathogenic (ClinVar) |  |
| 56192598 | c.143C>T (p.Thr48Ile) | rs1555499800 | *GNAO1A* | ENST00000262493 | 1.0 | D | 0.935 | D | 0.939 | D | 0.485927 | D | 0.99985146522522 | D | Likely Pathogenic (ClinVar) |  |
| 56192610 | c.155A>C (p.Gln52Pro) | . | *GNAO1A* | ENST00000262493 | 1.0 | D | 0.917 | D | 0.955 | D | 0.516164 | D | 0.999384164810181 | D | Pathogenic | (Rim et al., 2018) |
| 56192610 | c.155A>G (p.Gln52Arg) | . | *GNAO1A* | ENST00000262493 | 0.999 | D | 0.871 | D | 0.938 | D | 0.510894 | D | 0.999569118022919 | D | Pathogenic | (Bobylova et al., 2023) |
| 56275936 | c.167T>C (p.Ile56Thr) | . | *GNAO1A* | ENST00000262493 | 0.917 | D | 0.848 | D | 0.951 | D | 0.47459 | D | 0.995525538921356 | D | Likely Pathogenic | (Danti et al., 2017). |
| 56275976 | c.207C>A (p.Tyr69Ter) | rs77558236 | *GNAO1A* | ENST00000262493 |  |  | 0.89 | D |  |  | 0.625005 | D |  |  | Likely Pathogenic (VarSome) |  |
| 56275989 | c.220T>G (p.Tyr74Asp) | . | *GNAO1A* | ENST00000262493 | 0.883 | D | 0.942 | D | 0.932 | D | 0.463288 | D | 0.99783331155777 | D | Pathogenic (LOVD) |  |
| 56328778 | c.451G>A (p.Asp151Asn) | rs1596867702 | *GNAO1A* | ENST00000262493 | 1.0 | D | 0.902 | D | 0.768 | D | 0.237639 | D | 0.999461114406586 | D | Likely Pathogenic (ClinVar, Decipher) |  |
| 56334734 | c.470T​>C (p.Leu157Pro) | . | *GNAO1A* | ENST00000262493 | 1.0 | D | 0.985 | D | 0.981 | D | 0.520554 | D | 0.999674439430237 | D | Likely Pathogenic (Decipher) | (Yang et al., 2021) |
| 56334749 | c.485G>A (p.Arg162Gln) | rs1240134140 | *GNAO1A* | ENST00000262493 | 0.947 | P | 0.948 | D | 0.878 | D | 0.49214 | D | 0.998919010162354 | D | Likely Pathogenic (VarSome) | (Bobylova et al., 2023) |
| 56334773 | c.509C>G (p.Pro170Arg) | . | *GNAO1A* | ENST00000262493 | 1.0 | D | 0.985 | D | 0.961 | D | 0.580692 | D | 0.999860167503357 | D | Likely Pathogenic (ClinVar) |  |
| 56334784 | c.520G>A (p.Asp174Asn) | rs1567488305 | *GNAO1A* | ENST00000262493 | 1.0 | D | 0.974 | D | 0.933 | D | 0.259532 | D | 0.999670028686523 | D | Pathogenic*** | (Shah, 2022) |
| 56334785 | c.521A>G (p.Asp174Gly) | rs587777055 | *GNAO1A* | ENST00000262493 | 1.0 | D | 0.985 | D | 0.988 | D | 0.559195 | D | 0.999401688575745 | D | Pathogenic | (Nakamura et al., 2013) |
| 56334791 | c.527_528delinsAA (p.Leu176Gln) | rs2037724743 | *GNAO1A* | ENST00000262493 | 1.0 | D |  |  |  |  |  |  |  |  | Likely Pathogenic (ClinVar) |  |
| 56334793 | c.529C>T (p.Arg177Ter) | rs2037724762 | *GNAO1A* | ENST00000262493 |  |  | 0.934 | D |  |  | 0.625005 | D |  |  | Pathogenic (ClinVar) |  |
| 56334794 | c.530G>C (p.Arg177Pro) | . | *GNAO1A* | ENST00000262493 | 1.0 | D | 0.968 | D | 0.943 | D | 0.493169 | D | 0.999681234359741 | D | Likely Pathogenic | (Muir et al., 2019) |
| 56334799 | c.535A>G (p.Arg179Gly) | . | *GNAO1A* | ENST00000262493 | 0.999 | D | 0.95 | D | 0.915 | D | 0.476341 | D | 0.998129665851593 | D | Pathogenic | (Graziola et al., 2019) |
| 56334809 | c.545C>T (p.Thr182Ile) | rs1596871452 | *GNAO1A* | ENST00000262493 | 1.0 | D | 0.977 | D | 0.989 | D | 0.558988 | D | 0.999319076538086 | D | Pathogenic*** | (Turro et al., 2020), this study |
| 56334814 | c.550G>A (p.Gly184Ser) | rs1555507383 | *GNAO1A* | ENST00000262493 | 0.988 | D | 0.928 | D | 0.954 | D | 0.545976 | 0.988 | 0.997089207172394 | D | Pathogenic***** | this study |
| 56334814 | c.550G>C (p.Gly184Arg) | rs1555507383 | *GNAO1A* | ENST00000262493 | 1.0 | D | 0.98 | D | 0.972 | D | 0.577724 | D | 0.999771773815155 | D | Likely Pathogenic (ClinVar) | (Powis et al., 2020) |
| 56334836 | c.572_592 del (p.Thr191_Phe197 del) | rs587777056 | *GNAO1A* | ENST00000262493 |  |  |  |  |  |  |  |  |  |  | Pathogenic (ClinVar) | (Nakamura et al., 2013) |
| 56336733 | c.596T>C (p.Leu199Pro) | . | *GNAO1A* | ENST00000262493 | 1.0 | D | 0.967 | D | 0.970 | D | 0.493836 | D | 0.999198257923126 | D | Pathogenic (ClinVar) | (Marce-Grau et al., 2016; Zou et al., 2021) |
| 56336739 | c.602A>T (p.Asp201Val) | . | *GNAO1A* | ENST00000262493 | 0.999 | D | 0.975 | D | 0.977 | D | 0.563685 | D | 0.999635457992554 | D | Pathogenic (ClinVar) |  |
| 56336741 | c.604G>A (p.Val202Ile) | rs1297388989 | *GNAO1A* | ENST00000262493 | 0.988 | B | 0.515 | D | 0.731 | D | 0.21699 | D | 0.996854364871979 | D | Likely pathogenic (ClinVar) |  |
| 56336744 | c.607G>A (p.Gly203Arg) | rs587777057 | *GNAO1A* | ENST00000262493 | 1.0 | D | 0.903 | D | 0.941 | D | 0.544023 | D | 0.99994957447052 | D | Pathogenic (ClinVar) | (Arisaka et al., 2021; Nakamura et al., 2013) (Yang et al., 2021), this study + many more see references Suppl. Table III |
| 56336745 | c.608G>A (p.Gly203Glu) | . | *GNAO1A* | ENST00000262493 | 1.0 | D | 0.928 | D | 0.954 | D | 0.56581 | D | 0.999874114990234 | D | Pathogenic (ClinVar) |  |
| 56336747 | c.610G>C (p.Gly204Arg) | . | *GNAO1A* | ENST00000262493 | 1.0 | D | 0.856 | D | 0.931 | D | 0.560053 | D | 0.999893188476562 | D | Pathogenic (ClinVar) | (Koy et al., 2018) |
| 56336748 | c.611G>a (p.Gly204Asp) | . | *GNAO1A* | ENST00000262493 | 1.0 | D | 0.88 | D | 0.947 | D | 0.584647 | D | 0.999665379524231 | D | Pathogenic (ClinVar) | (Yamamoto et al., 2022) |
| 56336751 | c.614A>C (p.Gln205Pro) | . | *GNAO1A* | ENST00000262493 | 1.0 | D | 0.909 | D | 0.970 | D | 0.470605 | D | 0.999668717384338 | D | Likely Pathogenic (ClinVar) |  |
| 56336753 | c.616C>T (p.Arg206Ter) | . | *GNAO1A* | ENST00000262493 |  |  | 0.959 | D |  |  | 0.625005 | D |  |  | Likely Pathogenic (VarSome) | (Froukh et al., 2020) |
| 56336754 | c.617G>A (p.Arg206Gln) | rs1297225571 | *GNAO1A* | ENST00000262493 | 1.0 | D | 0.839 | D | 0.859 | D | 0.427062 | D | 0.999897241592407 | D | Pathogenic*** | (Wirth et al., 2022), multiple patients this study |
| 56336754 | c.617G>T (p.Arg206Leu) | . | *GNAO1A* | ENST00000262493 | 1.0 | D | 0.921 | D | 0.932 | D | 0.516341 | D | 0.999881029129028 | D | Pathogenic (ClinVar) | (Kim et al., 2020) |
| 56336757 | c.620C>A (p.Ser207Tyr) | rs1057518440 | *GNAO1A* | ENST00000262493 | 1.0 | D | 0.908 | D | 0.913 | D | 0.429966 | D | 0.999233841896057 | D | Pathogenic (ClinVar) | (Kelly et al., 2019) |
| 56336757 | c.620C>T (p.Ser207Phe) | . | *GNAO1A* | ENST00000262493 | 1.0 | D | 0.897 | D | 0.938 | D | 0.487747 | D | 0.999664187431335 | D | Pathogenic (ClinVar) | (Yamashita et al., 2020) |
| 56336759 | c.622G>C (p.Glu208Gln) | . | *GNAO1A* | ENST00000262493 | 1.0 | D | 0.811 | D | 0.780 | D | 0.326867 | D | 0.993200123310089 | D | Pathogenic*** | (Wirth et al., 2022), this study |
| 56336762 | c.625C>G (p.Arg209Gly) | rs886039494 | *GNAO1A* | ENST00000262493 | 1.0 | D | 0.943 | D | 0.924 | D | 0.495304 | D | 0.999528288841248 | D | Pathogenic (ClinVar) | (Ananth et al., 2016) |
| 56336762 | c.625C>T (p.Arg209Cys) | rs886039494 | *GNAO1A* | ENST00000262493 | 1.0 | D | 0.947 | D | 0.888 | D | 0.503659 | D | 0.999947667121887 | D | Pathogenic (ClinVar) | (Danhofer et al., 2021) (Brunet et al., 2021) (Danti et al., 2017), (Dzinovic et al., 2021) (Kwong et al., 2021) (Schirinzi et al., 2019) (Waak et al., 2018) (Zech et al., 2020), this study |
| 56336763 | c.626G>A (p.Arg209His) | rs797044878 | *GNAO1A* | ENST00000262493 | 1.0 | D | 0.926 | D | 0.914 | D | 0.528566 | D | 0.999944090843201 | D | Pathogenic (ClinVar) | (Menke et al., 2016), (Zech et al., 2020), (Brunet et al., 2021) (Ananth et al., 2016) (Kulkarni, Tang, Bhardwaj, Bernes, & Grebe, 2016), this study |
| 56336763 | c.626G>C (p.Arg209Pro) | . | *GNAO1A* | ENST00000262493 | 1.0 | D | 0.943 | D | 0.925 | D | 0.523226 | D | 0.999591529369354 | D | Pathogenic (ClinVar) | (van der Ven et al., 2021) |
| 56336763 | c.626G>T (p.Arg209Leu) | rs797044878 | *GNAO1A* | ENST00000262493 | 1.0 | D | 0.948 | D | 0.925 | D | 0.526013 | D | 0.999902486801147 | D | Pathogenic (ClinVar) | (Matthews et al., 2019) |
| 56336781 | c.644G>A (p.Cys215Tyr) | . | *GNAO1A* | ENST00000262493 | 1.0 | D | 0.893 | D | 0.901 | D | 0.465205 | D | 0.999775230884552 | D | Pathogenic (ClinVar) | (Carecchio et al., 2019) |
| 56336786 | c.649G>A (p.Glu217Lys) | rs1555507477 | *GNAO1A* | ENST00000262493 | 0.905 | B | 0.894 | D | 0.841 | D | 0.453765 | D | 0.995627403259277 | D | Likely Pathogenic (ClinVar) |  |
| 56336799 | c.662C>A (p.Ala221Asp) | rs1555507479 | *GNAO1A* | ENST00000262493 | 1.0 | D | 0.934 | D | 0.951 | D | 0.486247 | D | 0.998945415019989 | D | Pathogenic | (May et al., 2021) |
| 56336813 | c.676_677del (p.Val226ArgfsTer6) | rs1064797211 | *GNAO1A* | ENST00000262493 |  |  |  |  |  |  |  |  |  |  | Likely Pathogenic (ClinVar) |  |
| 56336817 | c.680C>T (p.Ala227Val) | rs797045599 | *GNAO1A* | ENST00000262493 | 1.0 | D | 0.96 | D | 0.885 | D | 0.457163 | D | 0.999571859836578 | D | Pathogenic (ClinVar) | (Saitsu et al., 2016), this study |
| 56336820 | c.683T>C (p.Leu228Pro) | rs1085307932 | *GNAO1A* | ENST00000262493 | 0.998 | D | 0.989 | D | 0.964 | D | 0.514756 | D | 0.999055325984955 | D | Likely Pathogenic | (Nashabat et al., 2019) |
| 56336824 | c.687C>A (p.Ser229Arg) | rs546569747 | *GNAO1A* | ENST00000262493 | 1.0 | D | 0.972 | D | 0.883 | D | 0.456691 | D | 0.999123871326447 | D | Pathogenic** | this study |
| 56336824 | c.687C>G (p.Ser229Arg) | rs546569747 | *GNAO1A* | ENST00000262493 | 1.0 | D | 0.972 | D | 0.883 | D | 0.456691 | D | 0.999123871326447 | D | Pathogenic** | (Yang et al., 2021) |
| 56336829 | c.692A>G (p.Tyr231Cys) | rs1057518678 | *GNAO1A* | ENST00000262493 | 1.0 | D | 0.981 | D | 0.987 | D | 0.519862 | D | 0.999813258647919 | D | Pathogenic (ClinVar) | (Kelly et al., 2019; Rosello et al., 2021), this study |
| 56336835 | c.698A>C (p.Gln233Pro) | . | *GNAO1A* | ENST00000262493 | 0.995 | D | 0.87 | D | 0.889 | D | 0.463262 | D | 0.994879364967346 | D | Likely Pathogenic | (Yilmaz et al., 2016) |
| 56336846 | c.709G>A (p.Glu237Lys) | rs1064794533 | *GNAO1A* | ENST00000262493 | 0.999 | D | 0.89 | D | 0.922 | D | 0.531399 | D | 0.999806106090546 | D | Pathogenic (ClinVar) | Many studies (see Suppl. Table III) |
| 56336850 | c.713A>G (p.Asp238Gly) | . | *GNAO1A* | ENST00000262494 | 1.0 | D | 0.854 | D | 0.865 | D | 0.418373 | D | 0.997857511043549 | D | Likely Pathogenic | (Bobylova et al., 2023) |
| 56336861 | c.723+1G>A | rs1596872804 | *GNAO1A* | ENST00000262493 | insertion AAG A out of frame |  |  |  |  |  |  |  | Pathogenic (ClinVar) |  |  | (Bobylova et al., 2023) |
| 56336861 | c.723+1G>T | rs1596872804 | *GNAO1A* | ENST00000262493 | T241 AC spliced out out of frame | | |  |  |  |  |  |  |  | Likely Pathogenic | (Koy et al., 2018)­ |
| 56336862 | c.723+2T>A | . | *GNAO1A* | ENST00000262493 | insertion GAG A out of frame | | |  |  |  |  |  |  |  | Likely Pathogenic | (Danti et al., 2017). |
| 56351376 | c.724-8G>A (p.Thr241_Asn-242insProGln) | rs1085307876 | *GNAO1A* | ENST00000262493 |  | | |  |  |  |  |  |  |  | Pathogenic | Many studies (see Suppl. Table III) |
| 56351385 | c.725A>C (p.Asn242Thr) | rs1085307894 | *GNAO1A* | ENST00000262493 | 1.0 | D | 0.912 | D | 0.902 | D | 0.259001 | D | 0.994220018386841 | D | Pathogenic (ClinVar) | (Wirth et al., 2022) |
| 56351391 | c.731T>A (p.Met244Lys) | rs2037919953 | *GNAO1A* | ENST00000262493 | 1.0 | D | 0.959 | D | 0.951 | D | 0.492163 | D | 0.998570322990417 | D | Likely Pathogenic (ClinVar) |  |
| 56351396 | c.736G>A (p.Glu246Lys) | rs797044951 | *GNAO1A* | ENST00000262493 | 1.0 | D | 0.934 | D | 0.844 | D | 0.497423 | D | 0.999531865119934 | D | Pathogenic (ClinVar) | Many studies (see Suppl. Table III) |
| 56351396 | c.736G>C (p.Glu246Gln) | rs797044951 | *GNAO1A* | ENST00000262493 | 1.0 | D | 0.934 | D | 0.775 | D | 0.448716 | D | 0.998391032218933 | D | Likely Pathogenic (ClinVar) | (P. Liu et al., 2019) |
| 56351397 | c.737A>G (p.Glu246Gly) | rs1114167431 | *GNAO1A* | ENST00000262493 | 1.0 | D | 0.936 | D | 0.931 | D | 0.501147 | D | 0.998062074184418 | D | Likely Pathogenic | (Danti et al., 2017) |
| 56351397 | c.737A>T (p.Glu246Val) | . | *GNAO1A* | ENST00000262493 | 1.0 | D | 0.966 | D | 0.950 | D | 0.497482 | D | 0.992054462432861 | D | Likely Pathogenic | (Wirth et al., 2022) |
| 56351408 | c.748C>T (p.Leu250Phe) | . | *GNAO1A* | ENST00000262493 | 1.0 | D | 0.862 | D | 0.910 | D | 0.367048 | D | 0.99947053194046 | D | Pathogenic (ClinVar) |  |
| 56351425 | c.765dupT (p.Asn256Ter) | . | *GNAO1A* | ENST00000262494 |  |  |  |  |  |  |  |  |  |  | Likely Pathogenic | (Wirth et al., 2022) |
| 56351468 | c.808A>G (p.Asn270Asp) | . | *GNAO1A* | ENST00000262493 | 1.0 | D | 0.969 | D | 0.939 | D | 0.258656 | D | 0.988047957420349 | D | Pathogenic* | (Takata et al., 2019) |
| 56351468 | c.808A>C (p.Asn270His) | . | *GNAO1A* | ENST00000262493 | 1.0 | D | 0.939 | D | 0.974 | D | 0.337898 | D | 0.996622443199158 | D | Pathogenic | (E.-R. E. S. C. E. a. e.-R. E. S. u. a. b. Euro, Epilepsy Phenome/Genome, Epi, & Euro, 2017) |
| 56351469 | c.809A>C (p.Asn270Thr) | rs1596881854 | *GNAO1A* | ENST00000262493 | 1.0 | D | 0.946 | D | 0.928 | D | 0.341571 | D | 0.992316603660583 | D | Uncertain Significance (ClinVar), ****Likely Pathogenic |  |
| 56351470 | c.810C>A (p.Asn270Lys) | . | *GNAO1A* | ENST00000262493 | 1.0 | D | 0.908 | D | 0.849 | D | 0.264066 | D | 0.999241471290588 | D | Likely Pathogenic | (Yang et al., 2021) |
| 56351471 | c.811A>G (p.Lys271Glu) | rs1555508311 | *GNAO1A* | ENST00000262493 | 0.999 | D | 0.945 | D | 0.979 | D | 0.563077 | D | 0.994622349739075 | D | Likely Pathogenic (ClinVar) |  |
| 56351473 | c.813G>C (p.Lys271Asn) | rs758779535 | *GNAO1A* | ENST00000262493 | 1.0 | D | 0.92 | D | 0.791 | D | 0.414358 | D | 0.998906254768372 | D | Likely Pathogenic (ClinVar) |  |
| 56351477 | c.817G>T (p.Asp273Tyr) | . | *GNAO1A* | ENST00000262493 | 1.0 | D | 0.974 | D | 0.913 | D | 0.584744 | D | 0.999496221542358 | D | Likely Pathogenic (Decipher) | (Yang et al., 2021) |
| 56351478 | c.818A>T (p.Asp273Val) | rs2037920694 | *GNAO1A* | ENST00000262493 | 1.0 | D | 0.982 | D | 0.975 | D | 0.583281 | D | 0.997957706451416 | D | Likely Pathogenic | (Schirinzi et al., 2019) |
| 56351484 | c.824T>C (p.Phe275Ser) | . | *GNAO1A* | ENST00000262493 | 1.0 | D | 0.946 | D | 0.980 | D | 0.520521 | D | 0.995314121246338 | D | Pathogenic | (Zou et al., 2021) |
| 56351492 | c.833_835del (p.Lys278del) | rs2037920791 | *GNAO1A* | ENST00000262493 |  |  |  |  |  |  |  |  |  |  | Pathogenic*** | (Baldridge et al., 2017), this study |
| 56351496 | c.836T>A (p.Ile279Asn) | rs587777054 | *GNAO1A* | ENST00000262493 | 1.0 | D | 0.95 | D | 0.931 | D | 0.341576 | D | 0.999458372592926 | D | Pathogenic (ClinVar) | (Nakamura et al., 2013) |
| 56351504 | c.844T>C (p.Ser282Pro) | . | *GNAO1A* | ENST00000262493 | 1.0 | D | 0.66 | D | 0.870 | D | 0.416502 | D | 0.994602382183075 | D | Likely Pathogenic (ClinVar)* |  |
| 56351511 | c.851T>C (p.Leu284Ser) | rs1555508316 | *GNAO1A* | ENST00000262493 | 1.0 | D | 0.963 | D | 0.949 | D | 0.490295 | D | 0.992056906223297 | D | Pathogenic (ClinVar, Ensembl) | (Gerald et al., 2018) |
| 56351531 | c.871T>A (p.Tyr291Asn) | rs1064795384 | *GNAO1A* | ENST00000262493 | 1.0 | D | 0.959 | D | 0.941 | D | 0.479602 | D | 0.999166131019592 | D | Pathogenic (ClinVar) |  |
| 56351532 | c.872A>G (p.Tyr291Cys) | . | *GNAO1A* | ENST00000262493 | 1.0 | D | 0.89 | D | 0.939 | D | 0.423035 | D | 0.999013185501099 | D | Likely Pathogenic (VarSome) | (Shah, 2022) |
| 56354889 | c.901_903del (p.Ala301del) | . | *GNAO1A* | ENST00000262493 |  |  |  |  |  |  |  |  |  |  | Likely Pathogenic | on basis of (Kim et al., 2020) |
| 56354912 | c.924T>G (p.Phe308Leu) | rs57295392 | *GNAO1A* | ENST00000262493 | 0.568 | P | 0.803 | D | 0.820 | D | 0.36386 | D | 0.980157017707825 | D | Likely Pathogenic (VarSome) |  |
| 56354925 | c.937C>T (p.Arg313Cys) | . | *GNAO1A* | ENST00000262493 | 0.956 | P | 0.691 | D | 0.706 | D | 0.384401 | D | 0.996482014656067 | D | Likely Pathogenic (VarSome) |  |
| 56354961 | c.973T>G (p.Cys325Gly) | rs2037954227 | *GNAO1A* | ENST00000262493 | 1.0 | D | 0.887 | D | 0.930 | D | 0.449467 | D | 0.999951004981995 | D | Likely Pathogenic (ClinVar) |  |
| 56354968 | c.980C>A (p.Thr327Lys) | . | *GNAO1A* | ENST00000262493 | 1.0 | D | 0.929 | D | 0.930 | D | 0.493439 | D | 0.999653339385986 | D | Likely Pathogenic | (Caswell, Gunning, Owens, Ellard, & Wright, 2022) |
| 56354971 | c.983A>G (p.Asp328Gly) | rs878853051 | *GNAO1A* | ENST00000262493 | 1.0 | D | 0.941 | D | 0.949 | D | 0.514684 | D | 0.999679327011108 | D | Likely Pathogenic (ClinVar, Ensembl) |  |
| 56355001 | c.1013_1015del (p.Ala338del) | . | *GNAO1A* | ENST00000262493 |  |  |  |  |  |  |  |  |  |  | Likely Pathogenic | (Kim et al., 2020) |
| 56355018 | c.1030_1032delATT (p.Ile344del) | rs2037954707 | *GNAO1A* | ENST00000262493 |  |  |  |  |  |  |  |  |  |  | Pathogenic*** | (Kelly et al., 2019), this study |

* (Likely) pathogenic on basis of an *in vitro* study (Slepak et al., 1993)

** Pathogenic on basis of our unpublished functional data

*** Pathogenic on basis of multiple NDD patients with the same variant

**** Likely pathogenic on basis of multiple NDD patients with similar variants

***** Pathogenic on basis of Kehrl et al. (Kehrl et al., 2014)

**Table S3. Overview of GNAO1 patients and their phenotypes.**

| **GNAO1 VARIANT** | **SEX** | **PHENOTYPE** | **ONSET E** | **ONSET MD** | **STRUCTURAL CHANGES BRAIN** | **HYPOTONIA** | **DEVELOPMENTAL DELAY** | **SPEECH** | **RESPONSE MD TREATMENT** | **RESPONSE E TREATMENT** | **REFERENCE** |
| --- | --- | --- | --- | --- | --- | --- | --- | --- | --- | --- | --- |
| c.68T > C (p.Leu23Pro) | F | MD, E, DD | 4y | 12y | Normal | Absent | Present (Mild) | Not affected | Partially refractory | NA | [(Wirth et al., 2022)](https://movementdisorders.onlinelibrary.wiley.com/doi/full/10.1002/mds.29074) |
| c.111_113delCCT (p.Leu39del) | F | MD, DD | NA | NA | NA | NA | Present | NA | NA | NA | (Deciphering Developmental Disorders, 2017) |
| c.115+1G​>A | NA | MD, DD | NA | NA | NA | NA | Present | Absent | NA | NA | Decipher |
| c.118G>A (p.Gly40Arg) | M | DEE, DD | 6d | NA | Abnormal | Present | Present | NA | NA | NA | (Bruun et al., 2018) |
| c.118G>A (p.Gly40Arg) | F | MD, DEE, DD | 4d | NA | Normal | Present | Present | NA | NA | NA | (Deciphering Developmental Disorders, 2015) |
| c.118G>A (p.Gly40Arg) | NA | DEE | NA | NA | NA | NA | NA | NA | NA | NA | (Lecoquierre F., 2018) |
| c.118G>C (p.Gly40Arg) | F | MD, DEE, DD | 2m | 3y | Abnormal (MC, TCC) | NA | Present (Severe) | Absent | NA | Refractory | (Danti et al., 2017) |
| c.118G>C (p.Gly40Arg) | M | MD, DEE, DD | 2.5m | NA | Abnormal | Present | Present | Absent | NA | NA | [(Kelly et al., 2019)](https://onlinelibrary.wiley.com/doi/abs/10.1111/epi.14653) |
| c.118G>C (p.Gly40Arg) | F | DEE, DD | NA | NA | Normal | NA | Present | NA | NA | NA | This study |
| c.118G>C (p.Gly40Arg) | F | DEE, DD | NA | NA | NA | NA | Present (Moderate) | NA | NA | NA | (Deciphering Developmental Disorders, 2017) |
| c.118G>C (p.Gly40Arg) | NA | DEE, DD | NA | NA | NA | NA | Present | NA | NA | NA | (Takata et al., 2019) |
| c.118G>C (p.Gly40Arg) | M | DEE, DD | 4m | NA | Abnormal | Present | Present (Moderate) | NA | NA | Symptom-free | [(Yang et al., 2021)](https://www.frontiersin.org/articles/10.3389/fneur.2021.662162/full) |
| c.118G>C (p.Gly40Arg) | F | DEE, DD | 0h | NA | NA | Absent | Present | NA | NA | NA | This study |
| c.118G>T (p.Gly40Trp) | M | DEE, DD | NA | NA | NA | NA | Present | NA | NA | NA | (Rim et al., 2018) |
| c.118G>T (p.Gly40Trp) | F | DEE, DD | 5wk | NA | Abnormal (AT, MY) | Present | Present | Absent | NA | NA | [(Kelly et al., 2019)](https://onlinelibrary.wiley.com/doi/abs/10.1111/epi.14653) |
| c.119G>A (p.Gly40Glu) | M | DEE, DD | 15h | NA | Abnormal (AT) | Present | Present | Absent | NA | NA | [(Kelly et al., 2019)](https://onlinelibrary.wiley.com/doi/abs/10.1111/epi.14653) |
| c.119G>A (p.Gly40Glu) | F | MD, DEE, DD | 2h | NA | Abnormal | Present | Present | Absent | NA | NA | [(Kelly et al., 2019)](https://onlinelibrary.wiley.com/doi/abs/10.1111/epi.14653) |
| c.124G>A (p. Gly42Arg) | F | MD, DD | NA | 4y | Normal | Absent | Present (Moderate) | Affected | Refractory | NA | (Y. Liu et al., 2022) |
| c.124G​>C (p.Gly42Arg) | M | MD, E, DD | NA | NA | NA | Present | Present (Severe) | NA | NA | NA | (Zhu et al., 2015) |
| c.133G>C (p.Gly45Arg) | M | MD, DEE, DD | NA | NA | NA | Present | Present (Severe) | NA | NA | Partially refractory | (Powis et al., 2020) |
| c.134G>A (p.Gly45Glu) | F | DEE, DD | 4d | NA | Abnormal (MC, AT) | Present | Present (Severe) | NA | NA | NA | (Gawlinski et al., 2016) |
| c.136A>G (p.Lys46Glu) | F | DEE, DD, deceased | 6h | NA | Normal | Present | Present (Moderate) | NA | NA | Refractory | [(Yang et al., 2021)](https://www.frontiersin.org/articles/10.3389/fneur.2021.662162/full) |
| c.137A>G (p.Lys46Arg) | F | MD | NA | 28m | Normal | Present | Absent | Affected | NA | NA | [(Wirth et al., 2022)](https://movementdisorders.onlinelibrary.wiley.com/doi/full/10.1002/mds.29074) |
| c.138A>T (p.Lys46Asn) | NA | MD | NA | 4m | Normal | Present | Present | Absent | Partially refractory | NA | (Thiel et al., 2023) |
| c.139A>G (p.Ser47Gly) | M | MD, E, DD, deceased | 3y | 4m | Abnormal (TCC) | Present | Present (Severe) | Absent | Partially refractory | Symptom-free | (Danti et al., 2017) |
| c.139A>G (p.Ser47Gly) | F | MD, DD | NA | NA | Normal | Present | Present (Moderate) | Absent | NA | NA | This study |
| c.141C>A (p.Ser47Arg) | M | MD, DEE, DD, deceased | NA | NA | NA | Present | Present (Severe) | Absent | Partially refractory | Refractory | This study |
| c.155A>C (p.Gln52Pro) | NA | DEE, DD | NA | NA | NA | NA | Present | NA | NA | NA | (Rim et al., 2018) |
| c.155A>G (p.Gln52Arg) | M | MD, DEE, DD, deceased | 1.5 | NA | Abnormal (MY) | NA | Present (Severe) | NA | NA | Symptom-free | (Solis et al., 2021) |
| c.164T>A (p.Ile55Asn) | F | DD | NA | NA | NA | Present | Present | Absent | NA | NA | This study |
| c.167T>C (p.Ile56Thr) | F | MD, E, DD | 4y | 5y | Abnormal | Absent | Present (Mild) | Not affected | NA | Symptom-free | (Danti et al., 2017) |
| c.170A>C (p.His57Pro) | NA | MD, E, DD | Neonatal | Neonatal | Abnormal (AT) | Present | Present | Absent | Partially refractory | Partially refractory | (Thiel et al., 2023) |
| c.218T>A (p.Val73Asp) | NA | MD, DEE, DD, deceased | Neonatal | NA | Abnormal (AT, MY) | Present | Present (Severe) | Absent | Partially refractory | Partially refractory | (Thiel et al., 2023) |
| c.220T>G (p.Tyr74Asp) | F | MD, DD | NA | NA | NA | NA | Present (Severe) | NA | NA | NA | (Deciphering Developmental Disorders, 2015) |
| c.425A>C (p.Asn142Thr) | F | E | NA | NA | NA | NA | NA | NA | NA | NA | (I. Helbig et al., 2020) |
| c.470T>C (p.Leu157Arg) | F | MD, DEE, DD | 1d | NA | Normal | Present | Present (Moderate) | NA | NA | Symptom-free | [(Yang et al., 2021)](https://www.frontiersin.org/articles/10.3389/fneur.2021.662162/full) |
| c.470T​>C (p.Leu157Pro) | F | E, DD | NA | NA | NA | Present | Present (Severe) | NA | NA | NA | Decipher |
| c.485G> A (p.Arg162Gln) | NA | MD, DEE, DD | <3m | NA | NA | Present | Present | Affected | NA | Refractory | (Bobylova et al., 2023) |
| c.521A>G (p.Asp174Gly) | F | DEE, DD | 29d | NA | Abnormal (MY, TCC) | Absent | Present (Severe) | Affected | NA | Refractory | [(Nakamura et al., 2013)](https://pubmed.ncbi.nlm.nih.gov/23993195/) |
| c.520G>A (p.Asp174Asn) | F | MD, E, DD, deceased | 3m | 8m | NA | NA | Present | NA | NA | Refractory | (Shah, 2022) |
| c.530G>C (p.Arg177Pro) | NA | MD, DEE, DD | NA | NA | Abnormal (MC) | NA | Present (Severe) | NA | NA | NA | (Muir et al., 2019) |
| c.535A>G (p.Arg179Gly) | M | MD, E, DD | NA | 12y | Normal | Present | Present (Mild) | Affected | Partially refractory | Partially refractory | (Graziola et al., 2019) |
| c.545C>T (p.Thr182Ile) | NA | MD, E | NA | NA | NA | NA | NA | NA | NA | NA | (Turro et al., 2020) |
| c.545C>T (p.Thr182Ile) | F | MD, E, deceased | NA | 3m | NA | Present | NA | NA | NA | NA | This study |
| c.545C>T (p.Thr182Ile) | F | MD, E | NA | 3m | NA | Present | NA | NA | NA | NA | This study |
| c.550G>A (p.Gly184Ser) | M |  | NA |  |  | Present |  |  |  |  | This study |
| c.550G>C (p.Gly184Arg) | NA | MD | NA | NA | NA | NA | NA | NA | NA | NA | (Powis et al., 2020) |
| c.572_592del (p.Thr191_Phe197 del) | F | MD, DEE, DD, deceased | 2wk | NA | Normal | Absent | Present (Severe) | Affected | NA | Refractory | [(Nakamura et al., 2013)](https://pubmed.ncbi.nlm.nih.gov/23993195/) |
| c.594-2​del | F | E, DD | NA | NA | NA | NA | Present (Severe) | Absent | NA | NA | Decipher |
| c.596T>C (p.Leu199Pro) | F | MD, DEE, DD, deceased | 3d | 3m | Abnormal (MC, AT, TCC, MY) | Present | Present (Severe) | NA | NA | Partially refractory | (Marce-Grau et al., 2016) |
| c.596T>C (p.Leu199Pro) | M | DEE,DD | 4d | NA | NA | NA | Present | NA | NA | NA | (Zou et al., 2021) |
| c.607G>A (p.Gly203Arg) | F | MD, DEE, DD | 2d | 16m | Normal | NA | Present | NA | Partially refractory | Refractory | (Arisaka et al., 2021) |
| c.607G>A (p.Gly203Arg) | M | MD, DEE, DD | 1m | NA | Abnormal (AT) | Absent | Present (Moderate) | NA | NA | Partially refractory | (Arya, Spaeth, Gilbert, Leach, & Holland, 2017) |
| c.607G>A (p.Gly203Arg) | F | MD, DEE, DD | 1mo | NA | NA | NA | Present | NA | NA | NA | (Fernandez-Marmiesse et al., 2019) |
| c.607G>A (p.Gly203Arg) | F | E | NA | NA | NA | NA | NA | NA | NA | NA | (Fernandez-Marmiesse et al., 2019) |
| c.607G>A (p.Gly203Arg) | F | MD, DEE, DD | 1.5mo | 18m | NA | Present | Present | NA | NA | NA | This study |
| c.607G>A (p.Gly203Arg) | F | MD, DEE, DD | <1d | 12h | NA | Present | Present | NA | NA | NA | This study |
| c.607G>A (p.Gly203Arg) | F | MD, E | 3y | <1y | NA | Present | NA | NA | NA | NA | This study |
| c.607G>A (p.Gly203Arg) | F | DEE, DD | 5h | NA | NA | Present | Present | NA | NA | NA | This study |
| c.607G>A (p.Gly203Arg) | M | E, DD | NA | NA | NA | NA | Present | NA | NA | NA | (Deciphering Developmental Disorders, 2017) |
| c.607G>A (p.Gly203Arg) | M | E, DD | NA | NA | NA | Present | Present (Severe) | NA | NA | NA | (Deciphering Developmental Disorders, 2017)) |
| c.607G>A (p.Gly203Arg) | F | MD, E, DD | 5y | NA | Abnormal (AT, MY, TCC) | Absent | Present (Severe) | Affected | NA | Refractory | [(Nakamura et al., 2013)](https://pubmed.ncbi.nlm.nih.gov/23993195/) |
| c.607G>A (p.Gly203Arg) | M | MD, DEE, DD, deceased | 3d | 3m | NA | NA | Present | NA | NA | NA | This study |
| c.607G>A (p.Gly203Arg) | F | MD, DEE, DD | 6wk | <1y | NA | NA | Present | NA | NA | Symptom-free | This study |
| c.607G>A (p.Gly203Arg) | F | MD, DEE, DD | 2wk | NA | NA | NA | Present | NA | NA | Symptom-free | (Pawłowicz, 2021) |
| c.607G>A (p.Gly203Arg) | F | MD | NA | NA | NA | NA | NA | NA | NA | NA | (Retterer et al., 2016) |
| c.607G>A (p.Gly203Arg) | F | MD, DEE, DD | 1wk | NA | Abnormal (AT, MY) | NA | Present (Severe) | Absent | NA | Refractory | (Saitsu et al., 2016) |
| c.607G>A (p.Gly203Arg) | F | MD, DEE, DD | 1d | 11m | Abnormal (TCC) | Present | Present (Severe) | Absent | Partially refractory | Symptom-free | [(Schirinzi et al., 2019)](https://www.sciencedirect.com/science/article/pii/S1353802018304978?via%3Dihub) |
| c.607G>A (p.Gly203Arg) | M | MD, DEE, DD | 12d | 2y | Abnormal (AT, MY) | Present | Present | Absent | Partially refractory | Symptom-free | [(Schirinzi et al., 2019)](https://www.sciencedirect.com/science/article/pii/S1353802018304978?via%3Dihub) |
| c.607G>A (p.Gly203Arg) | F | MD, DEE, DD | 3mo | NA | Abnormal (AT, TCC) | Absent | Present | NA | Refractory | Refractory | [(Schorling, 2017)](https://pubmed.ncbi.nlm.nih.gov/28628939/) |
| c.607G>A (p.Gly203Arg) | F | MD, DEE, DD | 9d | NA | Abnormal (AT) | Present | Present | NA | Partially refractory | Symptom-free | [(Schorling, 2017)](https://pubmed.ncbi.nlm.nih.gov/28628939/) |
| c.607G>A (p.Gly203Arg) | NA | DEE, DD | NA | NA | NA | NA | Present | NA | NA | NA | (Takata et al., 2019) |
| c.607G>A (p.Gly203Arg) | F | DEE, DD, deceased | NA | NA | Normal | Present | Present | NA | NA | Symptom-free | (Xiong et al., 2018) |
| c.607G>A (p.Gly203Arg) | M | MD, DEE, DD, deceased | 12d |  | Normal | Present | Present (Moderate) | NA | Refractory | Symptom-free | [(Yang et al., 2021)](https://www.frontiersin.org/articles/10.3389/fneur.2021.662162/full) |
| c.607G>A (p.Gly203Arg) | NA | MD, DEE, DD, deceased | <3m | 2-8m | NA | Present | Present | NA | NA | Refractory | (Bobylova et al., 2023) |
| c.607G>A (p.Gly203Arg) | NA | MD, DEE, DD, deceased | <3m | 2-8m | NA | Present | Present | NA | NA | Refractory | (Bobylova et al., 2023) |
| c.607G>A (p.Gly203Arg) | NA | MD, DEE, DD | <3m | 2-8m | NA | Present | Present | NA | NA | Refractory | (Bobylova et al., 2023) |
| c.607G>A (p.Gly203Arg) | NA | MD, DEE, DD | <3m | 2-8m | NA | Present | Present | NA | NA | Refractory | (Bobylova et al., 2023) |
| c.607G>A (p.Gly203Arg) | NA | MD, DEE, DD | <3m | 2-8m | NA | Present | Present | NA | NA | Refractory | (Bobylova et al., 2023) |
| c.607G>A (p.Gly203Arg) | NA | MD, DEE, DD | <3m | 2-8m | NA | Present | Present | NA | NA | Symptom-free | (Bobylova et al., 2023) |
| c.607G>A (p.Gly203Arg) | F | MD, DD, deceased | NA | 4m | NA | NA | Present | NA | NA | NA | (Shah, 2022) |
| c.607G>A (p.Gly203Arg) | F | MD, E, DD | 1y | 4y | Abnormal (MC, AT, TCC) | Present | Present | NA | Partially refractory | Symptom-free | (Novelli et al., 2023) |
| c.607G>A (p.Gly203Arg) | F | MD, E, DD | 21d | 9y | Abnormal (AT) | Present | Present | NA | Refractory | Partially refractory | (Novelli et al., 2023) |
| c.607G>A (p.Gly203Arg) | NA | MD, DEE, DD | 1m | NA | Normal | Present | Present | Absent | NA | Partially refractory | (Thiel et al., 2023) |
| c.607G>A (p.Gly203Arg) | NA | MD, DEE, DD | 1wk | NA | Abnormal (AT) | Present | Present | Absent | Partially refractory | Partially refractory | (Thiel et al., 2023) |
| c.607G>A (p.Gly203Arg) | NA | MD, DEE, DD | 2m | NA | Abnormal (AT) | Present | Present | Absent | Partially refractory | Partially refractory | (Thiel et al., 2023) |
| c.607G>A (p.Gly203Arg) | NA | MD, DEE, DD | 1m | NA | Abnormal (AT) | Present | Present | Absent | Partially refractory | Partially refractory | (Thiel et al., 2023) |
| c.607G>A (p.Gly203Arg) | NA | MD, DEE, DD | 1m | NA | Normal | Present | Present | Absent | Partially refractory | Partially refractory | (Thiel et al., 2023) |
| c.607G>A (p.Gly203Arg) | NA | MD, DEE, DD | 1m | NA | Normal | Present | Present | Absent | Partially refractory | Partially refractory | (Thiel et al., 2023) |
| c.610G>C (p.Gly204Arg) | NA | MD, E, DD | 6m | 4y | Abnormal (AT) | Absent | Present | Absent | Refractory | Partially refractory | (Thiel et al., 2023) |
| c.610G>C (p.Gly204Arg) | M | MD, DEE, DD | 2y | 4y | Abnormal | Present | Present | Absent | Partially refractory | NA | (Koy et al., 2018) |
| c.611G > T (p.Gly204Asp) | F | MD, deceased | NA | 9y | NA | NA | NA | NA | Refractory | NA | (Yamamoto et al., 2022) |
| c.616C>T (p.Arg206Term) | M | MD, E, DD | NA | NA | NA | Present | Present | Affected | NA | NA | (Froukh et al., 2020) |
| c.617G>A (p.Arg206Gln) | M | MD | NA | 15y | Normal | Absent | Absent | Affected | Refractory | NA | [(Wirth et al., 2022)](https://movementdisorders.onlinelibrary.wiley.com/doi/full/10.1002/mds.29074) |
| c.617G>A (p.Arg206Gln) | F | MD | NA | 47y | Normal | Absent | Absent | Not affected | NA | NA | [(Wirth et al., 2022)](https://movementdisorders.onlinelibrary.wiley.com/doi/full/10.1002/mds.29074) |
| c.617G>A (p.Arg206Gln) | F | MD | NA | 30y | Normal | Absent | Absent | Affected | NA | NA | [(Wirth et al., 2022)](https://movementdisorders.onlinelibrary.wiley.com/doi/full/10.1002/mds.29074) |
| c.617G>A (p.Arg206Leu) | M | MD, DD | NA | 2y | Normal | NA | Present | Affected | NA | NA | [(Kim et al., 2020)](https://ojrd.biomedcentral.com/articles/10.1186/s13023-020-01594-3) |
| c.620C >T (p.Ser207Phe | F | MD, DD | NA | 3y | Normal | Present | Present | Affected | Partially refractory | NA | (Yamashita et al., 2020) |
| c.620C>A (p.Ser207Tyr) | M | MD, DD | NA | NA | Normal | Present | Present | Affected | NA | NA | [(Kelly et al., 2019)](https://onlinelibrary.wiley.com/doi/abs/10.1111/epi.14653) |
| c.622G>C (p.Glu208Gln) | F | MD | MA | 5y | Normal | Present | Absent | Affected | NA | NA | [(Wirth et al., 2022)](https://movementdisorders.onlinelibrary.wiley.com/doi/full/10.1002/mds.29074) |
| c.622G>C (p.Glu208Gln) | M | MD | NA | NA | NA | NA | NA | NA | NA | NA | This study |
| c.625C>G (p.Arg209Gly) | F | MD, DD, deceased | NA | 46m | Normal | Present | Present | Absent | Refractory | NA | (Ananth et al., 2016) |
| c.625C>T (p.Arg209Cys) | F | MD, DD | NA | NA | Normal | Present | Present (Moderate) | NA | Symptom-free | NA | (Akasaka et al., 2021) |
| c.625C>T (p.Arg209Cys) | F | MD | NA | NA | NA | NA | NA | NA | NA | NA | (Brunet et al., 2021) |
| c.625C>T (p.Arg209Cys) | M | MD | NA | NA | NA | NA | NA | NA | NA | NA | (Brunet et al., 2021) |
| c.625C>T (p.Arg209Cys) | M | MD, E, DD | 2y | 4y | NA | Present | Present | Affected | Partially refractory | Symptom-free | (Danhofer et al., 2021) |
| c.625C>T (p.Arg209Cys) | M | MD, E, DD | 2y | 7mo | Normal | Absent | Present (Severe) | Absent | NA | Symptom-free | (Danti et al., 2017) |
| c.625C>T (p.Arg209Cys) | M | MD, E, DD | 6y | 9y | Abnormal (MC, TCC) | Present | Present (Severe) | Absent | NA | Symptom-free | (Danti et al., 2017) |
| c.625C>T (p.Arg209Cys) | F | MD, E, DD | NA | 4mo | Normal | Present | Present (Moderate) | Affected | NA | NA | (Dzinovic et al., 2021) |
| c.625C>T (p.Arg209Cys) | NA | MD, DD | NA | Neonatal | Abnormal (AT) | Present | Present | Absent | Partially refractory | NA | (Thiel et al., 2023) |
| c.625C>T (p.Arg209Cys) | NA | MD, E, DD | Neonatal | NA | Abnormal (AT) | Present | Present | Affected | Partially refractory | Partially refractory | (Thiel et al., 2023) |
| c.625C>T (p.Arg209Cys) | NA | MD, E, DD | NA | 4m | Abnormal (AT, MY) | Present | Present | Affected | Partially refractory | Partially refractory | (Thiel et al., 2023) |
| c.625C>T (p.Arg209Cys) | F | MD, E, DD | NA | 6y | Abnormal (AT) | Absent | Present | Absent | Refractory | NA | (Koy et al., 2018) |
| c.625C>T (p.Arg209Cys) | M | MD, E, DD | 10y | 11y | Abnormal (AT) | Present | Present | Absent | Partially refractory | NA | (Koy et al., 2018) |
| c.625C>T (p.Arg209Cys) | M | MD, DD, deceased | NA | NA | Normal | NA | Present (Moderate) | NA | Symptom-free | NA | (Kwong et al., 2021) |
| c.625C>T (p.Arg209Cys) | F | MD, DD | NA | 17m | Abnormal | Present | Present (Moderate) | Affected | Partially refractory | NA | (Malaquias et al., 2019) |
| c.625C>T (p.Arg209Cys) | M | NA | NA | NA | NA | Absent | NA | NA | NA | NA | (Deciphering Developmental Disorders, 2017) |
| c.625C>T (p.Arg209Cys) | F | NA | NA | NA | NA | Present | NA | NA | NA | NA | (Deciphering Developmental Disorders, 2017) |
| c.625C>T (p.Arg209Cys) | M | MD, E | 8y | 10y | NA | Present | NA | NA | NA | NA | This study |
| c.625C>T (p.Arg209Cys) | F | MD, E, DD | 10-11y | 4y | Abnormal (AT, TCC) | NA | Present (Severe) | Affected | NA | NA | (Saitsu et al., 2016) |
| c.625C>T (p.Arg209Cys) | F | MD, DD | NA | 8y | Abnormal (MC, AT, MY) | Present | Present (Severe) | Affected | Partially refractory | NA | [(Schirinzi et al., 2019)](https://www.sciencedirect.com/science/article/pii/S1353802018304978?via%3Dihub) |
| c.625C>T (p.Arg209Cys) | M | MD, E, DD | NA | 1y | Normal | Present | Present | Affected | Partially refractory | Partially refractory | (Waak et al., 2018) |
| c.625C>T (p.Arg209Cys) | F | MD | NA | <12m | NA | NA | NA | NA | NA | NA | (Zech et al., 2020) |
| c.625C>T (p.Arg209Cys) | M | MD | NA | 3m | Abnormal (TCC) | Absent | NA | Affected | NA | NA | (Kelly et al., 2019) |
| c.625C>T (p.Arg209Cys) | M | MD, E, DD | 8m | 1y | Abnormal (MC, MY) | Present | Present (Severe) | Affected | NA | Symptom-free | (Novelli et al., 2023) |
| c.625C>T (p.Arg209Cys) | F | MD, E, DD | 4y | <1y | Normal | NA | Present | Absent | Refractory | Symptom-free | (Novelli et al., 2023) |
| c.626G>A (p.Arg209His) | F | MD, DD | NA | 1y | Normal | Present | Present (Severe) | Affected | NA | NA | [(Kim et al., 2020)](https://ojrd.biomedcentral.com/articles/10.1186/s13023-020-01594-3) |
| c.626G>A (p.Arg209His) | M | MD, DD | NA | 6m | Abnormal | Absent | Present (Moderate) | Absent | NA | NA | (Kelly et al., 2019) |
| c.626G>A (p.Arg209His) | M | MD, DD | NA | 3y | Abnormal (AT) | Present | Present (Moderate) | Affected | Symptom-free | NA | (Ananth et al., 2016) |
| c.626G>A (p.Arg209His) | M | MD, DD | NA | 6m | Abnormal (AT, MY, TCC) | Present | Present | Affected | NA | NA | (Blumkin et al., 2018) |
| c.626G>A (p.Arg209His) | M | MD, DD | NA | 10m | Normal | Present | Present | NA | Partially refractory | NA | (Dhamija, Mink, Shah, & Goodkin, 2016) |
| c.626G>A (p.Arg209His) | M | MD, E, DD | NA | 34m | Normal | Present | Present | NA | Refractory | NA | (Kulkarni et al., 2016) |
| c.626G>A (p.Arg209His) | M | MD, DD | NA | 18m | Normal | Absent | Present | NA | Refractory | NA | (Kulkarni et al., 2016) |
| c.626G>A (p.Arg209His) | M | MD, DD | NA | 15m | Normal | Present | Present | NA | Refractory | NA | (Marecos, Duarte, Alonso, Calado, & Moreira, 2018) |
| c.626G>A (p.Arg209His) | F | MD | NA | NA | NA | NA | NA | NA | NA | NA | (Brunet et al., 2021) |
| c.626G>A (p.Arg209His) | M | MD, DD | NA | 18m | Normal | Present | Present | Absent | NA | NA | (Menke et al., 2016) |
| c.626G>A (p.Arg209His) | F | MD, DD | NA | <12y | NA | NA | Present | NA | NA | NA | (Zech et al., 2020) |
| c.626G>A (p.Arg209His) | M | MD, DD | NA | NA | NA | NA | Present | Affected | NA | NA | (Hu W, 2023) |
| c.626G>A (p.Arg209His) | F | MD, DD | NA | 5y | Abnormal (AT) | NA | Present | Affected | Refractory | NA | (Novelli et al., 2023) |
| c.626G>A (p.Arg209His) | NA | MD, DD | NA | 4m | Normal | Present | Present (Mild) | Affected | Refractory | NA | (Thiel et al., 2023) |
| c.626G>C (p.Arg209Pro) | F | MD, E | NA | <2y | NA | NA | NA | Affected | NA | NA | (van der Ven et al., 2021) |
| c.626G>T (p.Arg209Leu) | M | MD, DD | NA | 18m | Abnormal (AT) | Absent | Present | NA | Refractory | NA | (Honey et al., 2018) |
| c.626G>T (p.Arg209Leu) | M | MD, DD | NA | NA | Normal | Present | Present | Affected | NA | NA | (Menke et al., 2016) |
| c.626G>T (p.Arg209Leu) | F | MD, DD | NA | 10y | Normal | NA | Present | Absent | Partially refractory | NA | (Schöne-Bake, 2018) |
| c.626G>T (p.Arg209Leu) | M | MD, E, DD | NA | NA | Abnormal | NA | Present (Moderate) | NA | NA | NA | (Matthews et al., 2019) |
| c.644G>A (p.Cys215Tyr) | M | MD | NA | 12y | Normal | NA | Absent | NA | Partially refractory | NA | (Carecchio et al., 2019) |
| c.644G>A (p.Cys215Tyr) | M | MD, DD | NA | 3y | Normal | Present | Present (Mild) | Affected | Partially refractory | NA | [(Wirth et al., 2022)](https://movementdisorders.onlinelibrary.wiley.com/doi/full/10.1002/mds.29074) |
| c.644G>A (p.Cys215Tyr) | F | MD | NA | 5y | Normal | Absent | Absent | Affected | NA | NA | [(Wirth et al., 2022)](https://movementdisorders.onlinelibrary.wiley.com/doi/full/10.1002/mds.29074) |
| c.644G>A (p.Cys215Tyr) | M | MD, DD | NA | 6y | Normal | Absent | Present (Mild) | Affected | Refractory | NA | [(Wirth et al., 2022)](https://movementdisorders.onlinelibrary.wiley.com/doi/full/10.1002/mds.29074) |
| c.644G>A (p.Cys215Tyr) | F | MD, DD | NA | 6y | Normal | Absent | Present (Mild) | Affected | Partially refractory | NA | [(Wirth et al., 2022)](https://movementdisorders.onlinelibrary.wiley.com/doi/full/10.1002/mds.29074) |
| c.662C>A (p. Ala221Asp) | F | MD, DD | NA | 9m | Normal | Present | Present (Mild) | Affected | NA | NA | [(Kelly et al., 2019)](https://onlinelibrary.wiley.com/doi/abs/10.1111/epi.14653) |
| c.662C>A (p. Ala221Asp) | NA | MD, DD | NA | NA | Normal | NA | Present | NA | NA | NA | (May et al., 2021) |
| c.674G>A (p.Cys225Tyr) | NA | MD | Neonatal | NA | Normal | Present | Present | Affected | NA | Symptom-free | (Thiel et al., 2023) |
| c.676_677delG1 (p.Val226Argfs*6) | NA | MD, E,DD | 1-5m | 2m | Abnormal | Present | Present | Affected | Partially refractory | Partially refractory | (Thiel et al., 2023) |
| c.680C>T (p.Ala227Val) | F | MD, E, DD | NA | NA | Normal | Present | Present (Severe) | Affected | NA | NA | [(Kim et al., 2020)](https://ojrd.biomedcentral.com/articles/10.1186/s13023-020-01594-3) |
| c.680C>T (p.Ala227Val) | F | DD | NA | NA | NA | NA | Present | NA | NA | NA | (Mahfouz et al., 2020) |
| c.680C>T (p.Ala227Val) | M | MD, E | 1m | NA | NA | NA | NA | NA | NA | Mild | This study |
| c.680C>T (p.Ala227Val) | M | E, DD | 1wk | NA | NA | Present | Present (Severe) | NA | NA | NA | This study |
| c.680C>T (p.Ala227Val) | F | MD, DEE, DD | 2m | NA | Abnormal (AT, TCC) | NA | Present (Severe) | Absent | NA | Refractory | (Saitsu et al., 2016) |
| c.683T>C (pLeu228Pro) | F | E, DD | 1m | NA | Abnormal (MC) | Present | Present | NA | NA | NA | (Nashabat et al., 2019) |
| c.687C>A (p.Ser229Arg) | F | DEE, DD, deceased | 2d | NA | Normal | Present | Present | NA | NA | Refractory | This study |
| c.687C>G (p.Ser229Arg) | M | MD, DEE, DD | 1.5m | NA | Abnormal (MY) | Present | Present (Moderate) | NA | NA | Partially refractory | [(Yang et al., 2021)](https://www.frontiersin.org/articles/10.3389/fneur.2021.662162/full) |
| c.692A>G (p.Tyr231Cys) | M | DEE, DD | 5d | NA | Abnormal | Present | Present | Affected | NA | NA | [(Kelly et al., 2019)](https://onlinelibrary.wiley.com/doi/abs/10.1111/epi.14653) |
| c.692A>G (p.Tyr231Cys) | F | MD, DEE, DD | 3m | NA | Abnormal (AT, MY, TCC) | NA | Present (Severe) | Absent | NA | NA | (Talvik et al., 2015) |
| c.692A>G (p.Tyr231Cys) | F | MD,DEE, DD | 3d | NA | Abnormal | Present | Present (Moderate) | NA | NA | Symptom-free | [(Yang et al., 2021)](https://www.frontiersin.org/articles/10.3389/fneur.2021.662162/full) |
| c.692A>G (p.Tyr231Cys) | M | MD, E | 5y | 2m | NA | Present | NA | NA | NA | NA | This study |
| c.692A>G (p.Tyr231Cys) | NA | MD, E, DD | NA | NA | NA | NA | Present | NA | NA | NA | (Rosello et al., 2021) |
| c.698A>C (p.Gln233Pro) | F | MD | NA | 2y | Normal | Present | Absent | NA | Partially refractory | NA | (Yilmaz et al., 2016) |
| c.698A>C (p.Gln233Pro) | M | MD, deceased | NA | NA | NA | Present | Absent | NA | NA | NA | (Yilmaz et al., 2016) |
| c.709G>A (p.Glu237Lys) | M | E | <1d | NA | Abnormal (MC) | Present | NA | NA | NA | NA | (Cordoba et al., 2018) |
| c.709G>A (p.Glu237Lys) | F | MD, E, DD | 5mo | NA | Normal | NA | Present | NA | NA | NA | (Fernandez-Marmiesse et al., 2019) |
| c.709G>A (p.Glu237Lys) | F | MD | NA | <6m | Normal | Present | NA | NA | NA | NA | This study |
| c.709G>A (p.Glu237Lys) | M | MD, DD | NA | 3m | Abnormal (AT) | Present | Present | Absent | Refractory | NA | (Koy et al., 2018) |
| c.709G>A (p.Glu237Lys) | M | MD, DD, deceased | NA | 4y | Normal | Present | Present | Absent | Refractory | NA | (Koy et al., 2018) |
| c.709G>A (p.Glu237Lys) | M | MD | NA | 1y | Normal | Present | NA | NA | NA | NA | (Marecos et al., 2018) |
| c.709G>A (p.Glu237Lys) | M | MD, DD | NA | NA | Normal | Present | Present | Affected | NA | NA | (Al Masseri, 2022) |
| c.709G>A (p.Glu237Lys) | M | MD, DD | NA | NA | NA | Present | Present | Affected | NA | NA | (Al Masseri, 2022) |
| c.709G>A (p.Glu237Lys) | M | MD, DD | NA | NA | NA | NA | Present | Affected | NA | NA | (Monies et al., 2019) |
| c.709G>A (p.Glu237Lys) | F | MD, DD | NA | 4y | Normal | Present | Present | Absent | NA | NA | (Okumura et al., 2018) |
| c.709G>A (p.Glu237Lys) | M | MD | NA | NA | NA | NA | NA | NA | NA | NA | (Pawłowicz, 2021) |
| c.709G>A (p.Glu237Lys) | F | MD | NA | 16m | Abnormal (MY) | Present | NA | NA | NA | NA | [(Schirinzi et al., 2019)](https://www.sciencedirect.com/science/article/pii/S1353802018304978?via%3Dihub) |
| c.709G>A (p.Glu237Lys) | NA | NA | NA | NA | NA | NA | NA | NA | NA | NA | (Turro et al., 2020) |
| c.709G>A (p.Glu237Lys) | NA | NA | NA | NA | NA | NA | NA | NA | NA | NA | (Turro et al., 2020) |
| c.709G>A (p.Glu237Lys) | M | MD, DD | NA | NA | Abnormal (AT) | Present | Present | Affected | Partially refractory | NA | (Waak et al., 2018) |
| c.709G>A (p.Glu237Lys) | F | MD | NA | NA | NA | Present | NA | Absent | Refractory | NA | (Fung et al., 2022) |
| c.709G>A (p.Glu237Lys) | NA | MD, DD | NA | NA | NA | NA | Present (Severe) | NA | NA | NA | (Zech et al., 2020) |
| c.709G>A [p.Glu237Lys]) | M | MD, DD | NA | NA | Abnormal | Present | Present (Moderate) | Affected | Partially refractory | NA | (Ling et al., 2022) |
| c.713A>G (p.Asp238Gly) | F | MD | NA | 3y | NA | Present | Absent | NA | Refractory | NA | (Bobylova et al., 2023) |
| c.723+1G>A | F | MD, DD, deceased | NA | 8m | Abnormal (MC, AT) | Present | Present (Severe) | Affected | NA | NA | (Danti et al., 2017) |
| c.723+1G>A | F | NA | NA | NA | NA | NA | NA | NA | NA | NA | (Deciphering Developmental Disorders, 2015) |
| c.723+1G>A | M | MD, DD | NA | <1y | Normal | Present | Present (Severe) | NA | Symptom-free | NA | (Novelli et al., 2023) |
| c.723+1G>T | F | MD, DD | NA | 3y | Abnormal (AT) | Present | Present | Absent | Refractory | NA | (Koy et al., 2018) |
| c.723+1G>T | NA | MD, DD | NA | 1m | Abnormal (AT) | Present | Present | Absent | Refractory | NA | (Thiel et al., 2023) |
| c.723+2T>A | M | MD | NA | 1y | NA | Present | NA | NA | NA | NA | This study |
| c.724-8G>A (pThr241_Asn-241insProGln) | NA | MD | NA | NA | NA | NA | Absent | NA | NA | NA | (Retterer et al., 2016) |
| c.724-8G>A (pThr241_Asn-241insProGln) | NA | MD, DD | NA | NA | NA | NA | Present | Affected | NA | NA | (Monies et al., 2019) |
| c.724-8G>A (pThr241_Asn-241insProGln) | M | MD, DD | NA | 2y | Normal | Present | Present (Mild) | Affected | Refractory | NA | [(Yang et al., 2021)](https://www.frontiersin.org/articles/10.3389/fneur.2021.662162/full) |
| c.724-8G>A (pThr241_Asn-241insProGln) | M | MD, DD | NA | 3y | Normal | Present | Present (Mild) | NA | Refractory | NA | [(Yang et al., 2021)](https://www.frontiersin.org/articles/10.3389/fneur.2021.662162/full) |
| c.724-8G>A (pThr241_Asn-241insProGln) | F | MD, DD | NA | NA | Normal | Absent | Present | Affected | Partially refractory | NA | (Al Masseri, 2022) |
| c.724-8G>A (pThr241_Asn-241insProGln) | F | MD, DD | NA | NA | Normal | Absent | Present | Affected | Partially refractory | NA | (Al Masseri, 2022) |
| c.724-8G>A (pThr241_Asn-241insProGln) | M | MD, DD | NA | NA | Normal | Absent | Present | Affected | NA | NA | (Al Masseri, 2022) |
| c.724-8G>A (pThr241_Asn-241insProGln) | F | MD, DD | NA | 4y | Normal | NA | Present (Mild) | Affected | Refractory | NA | (Miyamoto, Nakashima, Fukumura, Kumada, & Saitsu, 2022) |
| c.724-8G>A (pThr241_Asn-241insProGln) | M | MD, DD | NA | 5y | Normal | Absent | Present (Moderate) | Affected | Partially refractory | NA | [(Wirth et al., 2022)](https://movementdisorders.onlinelibrary.wiley.com/doi/full/10.1002/mds.29074) |
| c.724-8G>A (pThr241_Asn-241insProGln) | F | MD, DD | NA | 5y | Normal | Absent | Present (Mild) | Affected | Refractory | NA | [(Wirth et al., 2022)](https://movementdisorders.onlinelibrary.wiley.com/doi/full/10.1002/mds.29074) |
| c.724-8G>A (pThr241_Asn-241insProGln) | F | MD, DD | NA | 7y | Normal | Present | Present (Mild) | Affected | Refractory | NA | [(Wirth et al., 2022)](https://movementdisorders.onlinelibrary.wiley.com/doi/full/10.1002/mds.29074) |
| c.724-8G>A (pThr241_Asn-241insProGln) | F | MD, DD | NA | 7y | Normal | Absent | Present (Moderate) | Not affected | Refractory | NA | [(Wirth et al., 2022)](https://movementdisorders.onlinelibrary.wiley.com/doi/full/10.1002/mds.29074) |
| c.724-8G>A (pThr241_Asn-241insProGln) | M | MD, DD | NA | 11y | Normal | Present | Present (Moderate) | Affected | Partially refractory | NA | [(Wirth et al., 2022)](https://movementdisorders.onlinelibrary.wiley.com/doi/full/10.1002/mds.29074) |
| c.724-8G>A (pThr241_Asn-241insProGln) | M | MD, DD | NA | 11y | Normal | Present | Present (Mild) | Not affected | Partially refractory | NA | [(Wirth et al., 2022)](https://movementdisorders.onlinelibrary.wiley.com/doi/full/10.1002/mds.29074) |
| c.724-8G>A (pThr241_Asn-241insProGln) | F | MD, DD | NA | 2y | Normal | Present | Present (Mild) | Affected | Partially refractory | NA | [(Wirth et al., 2022)](https://movementdisorders.onlinelibrary.wiley.com/doi/full/10.1002/mds.29074) |
| c.724-8G>A (pThr241_Asn-241insProGln) | F | MD, DD | NA | 5y | Normal | Present | Present (Mild) | Affected | Partially refractory | NA | [(Wirth et al., 2022)](https://movementdisorders.onlinelibrary.wiley.com/doi/full/10.1002/mds.29074) |
| c.724-8G>A (pThr241_Asn-241insProGln) | M | MD, DD | NA | 2y | Normal | Absent | Present (Mild) | Affected | Refractory | NA | (Y. Liu et al., 2022) |
| c.724-8G>A (pThr241_Asn-241insProGln) | M | MD, DD | NA | 6m | Normal | Absent | Present (Mild) | Affected | Refractory | NA | (Y. Liu et al., 2022) |
| c.724-8G>A (pThr241_Asn-241insProGln) | M | NA | NA | NA | NA | Present | NA | Affected | NA | NA | This study |
| c.724-8G>A (pThr241_Asn-241insProGln) | F | MD, DD | NA | NA | NA | NA | Present (Mild) | Affected | NA | NA | This study |
| c.724-8G>A | M | MD, DD | NA | 11y | Normal | Present | Present | Affected | Partially refractory | NA | (Novelli et al., 2023) |
| c.725A>C (p.Asn242Thr) | M | MD, DD | NA | 2y | Normal | Absent | Present | Affected | Symptom-free | NA | [(Wirth et al., 2022)](https://movementdisorders.onlinelibrary.wiley.com/doi/full/10.1002/mds.29074) |
| c.730_731delATinsCG (p.Met244Arg) | NA | DEE, DD | NA | NA | Normal | NA | Present | NA | NA | Refractory | (Hamdan et al., 2017) |
| c.736G>A (p.Glu246Lys) | NA | DEE, DD | NA | NA | NA | NA | Present | NA | NA | NA | (Farwell et al., 2015) |
| c.736G>A (p.Glu246Lys) | M | MD, DD | NA | 4y | Normal | Present | Present | Absent | NA | NA | (Ananth et al., 2016) |
| c.736G>A (p.Glu246Lys) | F | MD, DD | NA | 4y | Abnormal (AT) | Present | Present | Absent | Partially refractory | NA | (Ananth et al., 2016) |
| c.736G>A (p.Glu246Lys) | F | MD, DD, deceased | NA | 6y | Abnormal (MC, AT) | Present | Present | Absent | Partially refractory | NA | (Ananth et al., 2016) |
| c.736G>A (p.Glu246Lys) | M | MD, DD | NA | 14y | Abnormal | Present | Present | Absent | Partially refractory | NA | (Ananth et al., 2016) |
| c.736G>A (p.Glu246Lys) | F | MD, DD | NA | NA | Normal | NA | Present (Severe) | Absent | NA | NA | (Saitsu et al., 2016) |
| c.736G>A (p.Glu246Lys) | F | MD, DD | NA | 4m | Abnormal (AT) | Present | Present | NA | Partially refractory | NA | (Waak et al., 2018) |
| c.736G>A (p.Glu246Lys) | F | MD, E, DD | 7m | 5m | Abnormal (AT) | Absent | Present | NA | NA | Symptom-free | [(Schorling, 2017)](https://pubmed.ncbi.nlm.nih.gov/28628939/) |
| c.736G>A (p.Glu246Lys) | M | MD | NA | 11m | Normal | Present | Absent | NA | Refractory | NA | [(Schorling, 2017)](https://pubmed.ncbi.nlm.nih.gov/28628939/) |
| c.736G>A (p.Glu246Lys) | F | MD, DD | NA | 16m | Abnormal (MY) | NA | Present | NA | NA | NA | (Takezawa et al., 2018) |
| c.736G>A (p.Glu246Lys) | F | MD, DD | NA | 4y | Abnormal (AT) | Present | Present (Severe) | NA | Refractory | NA | (Benato et al., 2019) |
| c.736G>A (p.Glu246Lys) | F | MD, DD | NA | 11m | NA | Absent | Present (Severe) | NA | Refractory | NA | (Benato et al., 2019) |
| c.736G>A (p.Glu246Lys) | M | MD, DD | NA | 2y | Abnormal (AT) | Present | Present (Severe) | Absent | NA | NA | [(Kim et al., 2020)](https://ojrd.biomedcentral.com/articles/10.1186/s13023-020-01594-3) |
| c.736G>A (p.Glu246Lys) | M | DD, deceased | NA | NA | Abnormal (MY) | Present | Present (Moderate) | NA | NA | NA | [(Yang et al., 2021)](https://www.frontiersin.org/articles/10.3389/fneur.2021.662162/full) |
| c.736G>A(p.Glu246Lys) | M | MD, DD | NA | 10m | NA | NA | Present | Affected | NA | NA | (Shah, 2022) |
| c.736G>A (p.Glu246Lys) | F | MD, E, DD | NA | NA | NA | NA | Present (Mild) | NA | NA | NA | Mor (personal communication) |
| c.736G>A (p.Glu246Lys) | NA | NA | NA | NA | NA | NA | NA | NA | NA | NA | This study |
| c.736G>A (p.Glu246Lys) | F | MD, DD | NA | 9m | NA | NA | Present | Affected | Partially refractory | NA | (Novelli et al., 2023) |
| c.736G>A (p.Glu246Lys) | F | MD, DD | NA | <1y | Normal | NA | Present | NA | NA | NA | (Novelli et al., 2023) |
| c.736G>A (p.Glu246Lys) | M | MD, E, DD | NA | 2y | Abnormal | Present | Present | NA | Partially refractory | Symptom-free | (Novelli et al., 2023) |
| c.736G>A (p.Glu246Lys) | M | MD, E, DD | 5y | 2y | Normal | Present | Present | Absent | NA | Symptom-free | (Novelli et al., 2023) |
| c.736G>A(p.Glu246Lys) | NA | MD, DD | NA | 1m | NA | Present | Present | Affected | NA | NA | (Thiel et al., 2023) |
| c.736G>A(p.Glu246Lys) | NA | MD, DD | NA | 4m | Normal | Present | Present | Absent | NA | NA | (Thiel et al., 2023) |
| c.736G>A(p.Glu246Lys) | NA | MD, E, DD | 10m | 5m | Abnormal | Present | Present (severe) | Affected | NA | Partially refractory | (Thiel et al., 2023) |
| c.736G>A(p.Glu246Lys) | NA | MD, DD | NA | 4m | Abnormal | Present | Present | Affected | Partially refractory | NA | (Thiel et al., 2023) |
| c.736G>C (p.Glu246Gln) | NA | NA | NA | NA | NA | NA | NA | NA | NA | NA | (P. Liu et al., 2019) |
| c.737_738insCGA (p.246_247insAsp /p.246_247insD) | M | DEE, DD | 3m | NA | NA | NA | Present | NA | NA | NA | (Zou et al., 2021) |
| c.737A>G (p.Glu246Gly) | M | MD, E, DD | 10y | 2y | Abnormal (AT) | Present | Present (Mild) | Affected | Refractory | NA | (Danti et al., 2017) |
| c.737A>T (p.Glu246Val) | F | MD, DD | NA | 6y | Normal | Present | Present (Mild) | Affected | Partially refractory | NA | [(Wirth et al., 2022)](https://movementdisorders.onlinelibrary.wiley.com/doi/full/10.1002/mds.29074) |
| c.765dupT (p.Asn256Term) | F | MD, E | NA | 6y | Normal | Absent | Absent | Affected | NA | NA | [(Wirth et al., 2022)](https://movementdisorders.onlinelibrary.wiley.com/doi/full/10.1002/mds.29074) |
| c.765dupT (p.Asn256Term) | F | MD | NA | 16y | Normal | Absent | Absent | Affected | Refractory | NA | [(Wirth et al., 2022)](https://movementdisorders.onlinelibrary.wiley.com/doi/full/10.1002/mds.29074) |
| c.808A>C (p.Asn270His) | F | DEE, DD | 3m | NA | Abnormal (AT) | Present | Present (Severe) | NA | NA | NA | (E.-R. E. S. C. E. a. e.-R. E. S. u. a. b. Euro et al., 2017) |
| c.808A>G (p.Asn270Asp) | NA | DEE, DD | NA | NA | NA | NA | Present | NA | NA | NA | (Takata et al., 2019) |
| c.810C>A (p.Asn270Lys) | M | MD, DEE, DD | 9d | NA | Normal | Present | Present (Moderate) | NA | NA | Refractory | [(Yang et al., 2021)](https://www.frontiersin.org/articles/10.3389/fneur.2021.662162/full) |
| c.817G>T (p.Asp273Tyr) | F | MD, DEE, DD | 2d | NA | Normal | Present | Present (Moderate) | NA | NA | Symptom-free | [(Yang et al., 2021)](https://www.frontiersin.org/articles/10.3389/fneur.2021.662162/full) |
| c.818A>T (p.Asp273Val)* | F | MD, DEE, DD | 2d | NA | Normal | Present | Present | Absent | NA | NA | [(Kelly et al., 2019)](https://onlinelibrary.wiley.com/doi/abs/10.1111/epi.14653) |
| c.818A>T (p.Asp273Val) | F | MD, DEE, DD | 2d | 26m | Normal | Present | Present | Absent | Partially refractory | Symptom-free | [(Schirinzi et al., 2019)](https://www.sciencedirect.com/science/article/pii/S1353802018304978?via%3Dihub) |
| c.824T>C (p.Phe275Ser) | F | DEE, DD | 3d | NA | NA | Present | Present | NA | NA | ref | (E.-R. E. S. C. Euro, Epilepsy Phenome/Genome, & Epi, 2014) |
| c.824T>C (p.Phe275Ser) | F | DEE, DD | NA | NA | NA | NA | Present (Severe) | NA | NA | NA | (Yuskaitis et al., 2018) |
| c.824T>C (p.Phe275Ser) | M | DEE, DD | 7m | NA | NA | NA | Present | NA | NA | NA | (Zou et al., 2021) |
| c.832_834delAAG (p.Lys278del) | F | DEE, DD | 2m | NA | NA | NA | Present | NA | NA | NA | (Baldridge et al., 2017) |
| c.832_834delAAG (p.Lys278del) | F | MD, DEE, DD | 2d | NA | Abnormal | Present | Present | NA | NA | Refractory | This study |
| c.832_834delAAG (p.Lys278del) | F | MD, DEE, DD | 2d | NA | Normal | Present | Present | NA | NA | Symptom-free | This study |
| c.836T>A (p.Ile279Asn) | F | DEE, DD | 4d | NA | Abnormal (AT) | Absent | Present (Severe) | Affected | NA | Refractory | [(Nakamura et al., 2013)](https://pubmed.ncbi.nlm.nih.gov/23993195/) |
| c.836T>A (p.Ile279Asn) | M | MD, DEE, DD | 9d | NA | NA | NA | Present (Severe) | NA | NA | NA | (Epi, 2016) |
| c.836T>A (p.Ile279Asn) | NA | MD, E, DD | NA | NA | Abnormal (MC) | Absent | Present | NA | NA | NA | (Karaca et al., 2018) |
| c.836T>A (p.Ile279Asn) | M | MD, DEE, DD | 1h | NA | Abnormal (AT, MY) | Present | Present | Affected | NA | NA | [(Kelly et al., 2019)](https://onlinelibrary.wiley.com/doi/abs/10.1111/epi.14653) |
| c.851T>C (p.Leu284Ser) | F | DEE, DD | 11d | NA | Abnormal (MC, AT) | Present | Present (Severe) | NA | NA | Refractory | (Gerald et al., 2018) |
| c.863T>C (p.Phe288Ser) | NA | MD, DD | NA | 1m | NA | Present | Present | Absent | NA | NA | (Thiel et al., 2023) |
| c.871T>A (p.Tyr291Asn) | F | DEE, DD | 2m | NA | Normal | Present | Present | Absent | NA | NA | [(Kelly et al., 2019)](https://onlinelibrary.wiley.com/doi/abs/10.1111/epi.14653) |
| c.871T>A (p.Tyr291Asn) | M | MD | NA | 15y | NA | Absent | NA | NA | NA | NA | This study |
| c.872A>G(p.Tyr291Cys) | M | DEE, DD | NA | NA | NA | NA | Present | NA | NA | Refractory | (Shah, 2022) |
| c.877+5A>G* | F | E, DD | NA | NA | NA | Absent | Present | NA | NA | NA | (Chuan et al., 2022) |
| c.901_903del (p.Ala301del) | M | MD, DD | NA | NA | Normal | Present | Present (Severe) | Affected | NA | NA | [(Kim et al., 2020)](https://ojrd.biomedcentral.com/articles/10.1186/s13023-020-01594-3) |
| c.901G>C (p.Val301Leu)* | M | MD, E | 6y | >6y | NA | NA | NA | NA | NA | NA | (Fernandez-Marmiesse et al., 2019) |
| c.980C > A, p.(Thr327Lys) | NA | MD, DD | NA | NA | NA | NA | Present | NA | NA | NA | (Caswell et al., 2022) |
| c.986del (p.Thr329Argfs*45) | NA | E, DD | 15m | NA | Normal | Absent | Present | Affected | NA | NA | (Thiel et al., 2023) |
| c.1013_1015del (p.Ala338del) | F | MD, DD | NA | 10d | Normal | Absent | Present | Affected | NA | NA | [(Kim et al., 2020)](https://ojrd.biomedcentral.com/articles/10.1186/s13023-020-01594-3) |
| c.1030_1032delATT (p.Ile344del) | F | MD, DD | NA | 1y | Normal | Present | Present (Mild) | Affected | NA | NA | (Kelly et al., 2019) |
| c.1030_1032delATT (p.Ile344del) | M | MD, E, DD | NA | NA | NA | Absent | Present | NA | NA | NA | This study |
| c.1030_1032delATT (p.Ile344del) | F | MD | NA | 7y | NA | Absent | NA | NA | NA | NA | This study |

F female, M male, MD movement disorder, DD developmental delay, DEE developmental epileptic encephalopathy, E epilepsy, AT atrophy, MY altered (de)myelination, MC microcephaly, TCC

thinned corpus callosum, NA not available

**Supplementary information references**

Akasaka, M., Kamei, A., Tanifuji, S., Asami, M., Ito, J., Mizuma, K., . . . Kosaki, K. (2021). GNAO1 mutation-related severe involuntary movements treated with gabapentin. Brain Dev, 43(4), 576-579. doi:10.1016/j.braindev.2020.12.002

Al Masseri, Z. A., M. (2022). Gonadal mosaicism in GNAO1 causing neurodevelopmental disorder with involuntary movements; two additional variants. Molecular Genetics and Metabolism Reports, 31.

Ananth, A. L., Robichaux-Viehoever, A., Kim, Y. M., Hanson-Kahn, A., Cox, R., Enns, G. M., . . . Bernstein, J. A. (2016). Clinical Course of Six Children With GNAO1 Mutations Causing a Severe and Distinctive Movement Disorder. Pediatr Neurol, 59, 81-84. doi:10.1016/j.pediatrneurol.2016.02.018

Arisaka, A., Nakashima, M., Kumada, S., Inoue, K., Nishida, H., Mashimo, H., . . . Fukuda, M. (2021). Association of early-onset epileptic encephalopathy with involuntary movements - Case series and literature review. Epilepsy Behav Rep, 15, 100417. doi:10.1016/j.ebr.2020.100417

Arya, R., Spaeth, C., Gilbert, D. L., Leach, J. L., & Holland, K. D. (2017). GNAO1-associated epileptic encephalopathy and movement disorders: c.607G>A variant represents a probable mutation hotspot with a distinct phenotype. Epileptic Disord, 19(1), 67-75. doi:10.1684/epd.2017.0888

Baldridge, D., Heeley, J., Vineyard, M., Manwaring, L., Toler, T. L., Fassi, E., . . . Shinawi, M. (2017). The Exome Clinic and the role of medical genetics expertise in the interpretation of exome sequencing results. Genet Med, 19(9), 1040-1048. doi:10.1038/gim.2016.224

Benato, A., Carecchio, M., Burlina, A., Paoloni, F., Sartori, S., Nosadini, M., . . . Antonini, A. (2019). Long-term effect of subthalamic and pallidal deep brain stimulation for status dystonicus in children with methylmalonic acidemia and GNAO1 mutation. J Neural Transm (Vienna), 126(6), 739-757. doi:10.1007/s00702-019-02010-2

Blumkin, L., Lerman-Sagie, T., Westenberger, A., Ben-Pazi, H., Zerem, A., Yosovich, K., & Lev, D. (2018). Multiple Causes of Pediatric Early Onset Chorea-Clinical and Genetic Approach. Neuropediatrics, 49(4), 246-255. doi:10.1055/s-0038-1645884

Bobylova, M. Y., Volkov, I. V., Gumennik, E. V., Rachmanina, O. A., Abramov, M. O., Volkova, O. K., . . . Petrukhin, A. S. (2023). [Encephalopathy GNAO1]. Zh Nevrol Psikhiatr Im S S Korsakova, 123(1), 122-130. doi:10.17116/jnevro2023123011122

Brunet, T., Jech, R., Brugger, M., Kovacs, R., Alhaddad, B., Leszinski, G., . . . Wagner, M. (2021). De novo variants in neurodevelopmental disorders-experiences from a tertiary care center. Clin Genet, 100(1), 14-28. doi:10.1111/cge.13946

Bruun, T. U. J., DesRoches, C. L., Wilson, D., Chau, V., Nakagawa, T., Yamasaki, M., . . . Mercimek-Andrews, S. (2018). Prospective cohort study for identification of underlying genetic causes in neonatal encephalopathy using whole-exome sequencing. Genet Med, 20(5), 486-494. doi:10.1038/gim.2017.129

Carecchio, M., Invernizzi, F., Gonzalez-Latapi, P., Panteghini, C., Zorzi, G., Romito, L., . . . Nardocci, N. (2019). Frequency and phenotypic spectrum of KMT2B dystonia in childhood: A single-center cohort study. Mov Disord, 34(10), 1516-1527. doi:10.1002/mds.27771

Caswell, R. C., Gunning, A. C., Owens, M. M., Ellard, S., & Wright, C. F. (2022). Assessing the clinical utility of protein structural analysis in genomic variant classification: experiences from a diagnostic laboratory. Genome Med, 14(1), 77. doi:10.1186/s13073-022-01082-2

Chuan, Z., Ruikun, C., Qian, L., Shiyue, M., Shengju, H., Yong, Y., . . . Xu, M. (2022). Genetic and Phenotype Analysis of a Chinese Cohort of Infants and Children With Epilepsy. Front Genet, 13, 869210. doi:10.3389/fgene.2022.869210

Cordoba, M., Rodriguez-Quiroga, S. A., Vega, P. A., Salinas, V., Perez-Maturo, J., Amartino, H., . . . Kauffman, M. A. (2018). Whole exome sequencing in neurogenetic odysseys: An effective, cost- and time-saving diagnostic approach. PLoS One, 13(2), e0191228. doi:10.1371/journal.pone.0191228

Danhofer, P., Zech, M., Balintova, Z., Balaz, M., Jech, R., & Oslejskova, H. (2021). Brittle Biballism-Dystonia in a Pediatric Patient with GNAO1 Mutation Managed Using Pallidal Deep Brain Stimulation. Mov Disord Clin Pract, 8(1), 153-155. doi:10.1002/mdc3.13118

Danti, F. R., Galosi, S., Romani, M., Montomoli, M., Carss, K. J., Raymond, F. L., . . . Guerrini, R. (2017). GNAO1 encephalopathy: Broadening the phenotype and evaluating treatment and outcome. Neurol Genet, 3(2), e143. doi:10.1212/NXG.0000000000000143

Deciphering Developmental Disorders, S. (2015). Large-scale discovery of novel genetic causes of developmental disorders. Nature, 519(7542), 223-228. doi:10.1038/nature14135

Deciphering Developmental Disorders, S. (2017). Prevalence and architecture of de novo mutations in developmental disorders. Nature, 542(7642), 433-438. doi:10.1038/nature21062

Dhamija, R., Mink, J. W., Shah, B. B., & Goodkin, H. P. (2016). GNAO1-Associated Movement Disorder. Mov Disord Clin Pract, 3(6), 615-617. doi:10.1002/mdc3.12344

Dzinovic, I., Skorvanek, M., Necpal, J., Boesch, S., Svantnerova, J., Wagner, M., . . . Zech, M. (2021). Dystonia as a prominent presenting feature in developmental and epileptic encephalopathies: A case series. Parkinsonism Relat Disord, 90, 73-78. doi:10.1016/j.parkreldis.2021.08.007

Epi, K. C. (2016). De Novo Mutations in SLC1A2 and CACNA1A Are Important Causes of Epileptic Encephalopathies. Am J Hum Genet, 99(2), 287-298. doi:10.1016/j.ajhg.2016.06.003

Euro, E.-R. E. S. C., Epilepsy Phenome/Genome, P., & Epi, K. C. (2014). De novo mutations in synaptic transmission genes including DNM1 cause epileptic encephalopathies. Am J Hum Genet, 95(4), 360-370. doi:10.1016/j.ajhg.2014.08.013

Euro, E.-R. E. S. C. E. a. e.-R. E. S. u. a. b., Epilepsy Phenome/Genome, P., Epi, K. C., & Euro, E.-R. E. S. C. (2017). De Novo Mutations in Synaptic Transmission Genes Including DNM1 Cause Epileptic Encephalopathies. Am J Hum Genet, 100(1), 179. doi:10.1016/j.ajhg.2016.12.012

Farwell, K. D., Shahmirzadi, L., El-Khechen, D., Powis, Z., Chao, E. C., Tippin Davis, B., . . . Tang, S. (2015). Enhanced utility of family-centered diagnostic exome sequencing with inheritance model-based analysis: results from 500 unselected families with undiagnosed genetic conditions. Genet Med, 17(7), 578-586. doi:10.1038/gim.2014.154

Fernandez-Marmiesse, A., Roca, I., Diaz-Flores, F., Cantarin, V., Perez-Poyato, M. S., Fontalba, A., . . . Martinez-Atienza, M. (2019). Rare Variants in 48 Genes Account for 42% of Cases of Epilepsy With or Without Neurodevelopmental Delay in 246 Pediatric Patients. Front Neurosci, 13, 1135. doi:10.3389/fnins.2019.01135

Froukh, T., Nafie, O., Al Hait, S. A. S., Laugwitz, L., Sommerfeld, J., Sturm, M., . . . Buchert, R. (2020). Genetic basis of neurodevelopmental disorders in 103 Jordanian families. Clin Genet, 97(4), 621-627. doi:10.1111/cge.13720

Fung, E. L., Mo, C. Y., Fung, S. T., Chan, A. Y., Lau, K. Y., Chan, E. K., . . . Poon, W. S. (2022). Deep brain stimulation in a young child with GNAO1 mutation - Feasible and helpful. Surg Neurol Int, 13, 285. doi:10.25259/SNI_166_2022

Gawlinski, P., Posmyk, R., Gambin, T., Sielicka, D., Chorazy, M., Nowakowska, B., . . . Wiszniewski, W. (2016). PEHO Syndrome May Represent Phenotypic Expansion at the Severe End of the Early-Onset Encephalopathies. Pediatric Neurology, 60, 83-87. doi:10.1016/j.pediatrneurol.2016.03.011

Gerald, B., Ramsey, K., Belnap, N., Szelinger, S., Siniard, A. L., Balak, C., . . . Narayanan, V. (2018). Neonatal epileptic encephalopathy caused by de novo GNAO1 mutation misdiagnosed as atypical Rett syndrome: Cautions in interpretation of genomic test results. Semin Pediatr Neurol, 26, 28-32. doi:10.1016/j.spen.2017.08.008

Graziola, F., Garone, G., Stregapede, F., Bosco, L., Vigevano, F., Curatolo, P., . . . Capuano, A. (2019). Diagnostic Yield of a Targeted Next-Generation Sequencing Gene Panel for Pediatric-Onset Movement Disorders: A 3-Year Cohort Study. Front Genet, 10, 1026. doi:10.3389/fgene.2019.01026

Hamdan, F. F., Myers, C. T., Cossette, P., Lemay, P., Spiegelman, D., Laporte, A. D., . . . Michaud, J. L. (2017). High Rate of Recurrent De Novo Mutations in Developmental and Epileptic Encephalopathies. Am J Hum Genet, 101(5), 664-685. doi:10.1016/j.ajhg.2017.09.008

Helbig, I., Barcia, G., Pendziwiat, M., Ganesan, S., Mueller, S. H., Helbig, K. L., . . . Group, F. G. S. (2020). Whole-exome and HLA sequencing in Febrile infection-related epilepsy syndrome. Ann Clin Transl Neurol, 7(8), 1429-1435. doi:10.1002/acn3.51062

Helbig, K. L., Farwell Hagman, K. D., Shinde, D. N., Mroske, C., Powis, Z., Li, S., . . . Helbig, I. (2016). Diagnostic exome sequencing provides a molecular diagnosis for a significant proportion of patients with epilepsy. Genet Med, 18(9), 898-905. doi:10.1038/gim.2015.186

Honey, C. M., Malhotra, A. K., Tarailo-Graovac, M., van Karnebeek, C. D. M., Horvath, G., & Sulistyanto, A. (2018). GNAO1 Mutation-Induced Pediatric Dystonic Storm Rescue With Pallidal Deep Brain Stimulation. J Child Neurol, 33(6), 413-416. doi:10.1177/0883073818756134

Hu W, F. H., Tang J, Zhou Z, Wu L. . (2023). [Genetic analysis of a child with early onset neurodevelopmental disorder with involuntary movement and a literature review]. Zhonghua Yi Xue Yi Chuan Xue Za Zhi., 40(4), 385-389.

Karaca, E., Posey, J. E., Coban Akdemir, Z., Pehlivan, D., Harel, T., Jhangiani, S. N., . . . Lupski, J. R. (2018). Phenotypic expansion illuminates multilocus pathogenic variation. Genet Med, 20(12), 1528-1537. doi:10.1038/gim.2018.33

Kehrl, J. M., Sahaya, K., Dalton, H. M., Charbeneau, R. A., Kohut, K. T., Gilbert, K., . . . Neubig, R. R. (2014). Gain-of-function mutation in Gnao1: a murine model of epileptiform encephalopathy (EIEE17)? Mamm Genome, 25(5-6), 202-210. doi:10.1007/s00335-014-9509-z

Kelly, M., Park, M., Mihalek, I., Rochtus, A., Gramm, M., Perez-Palma, E., . . . Poduri, A. (2019). Spectrum of neurodevelopmental disease associated with the GNAO1 guanosine triphosphate-binding region. Epilepsia, 60(3), 406-418. doi:10.1111/epi.14653

Kim, S. Y., Shim, Y., Ko, Y. J., Park, S., Jang, S. S., Lim, B. C., . . . Chae, J. H. (2020). Spectrum of movement disorders in GNAO1 encephalopathy: in-depth phenotyping and case-by-case analysis. Orphanet J Rare Dis, 15(1), 343. doi:10.1186/s13023-020-01594-3

Koy, A., Cirak, S., Gonzalez, V., Becker, K., Roujeau, T., Milesi, C., . . . Cif, L. (2018). Deep brain stimulation is effective in pediatric patients with GNAO1 associated severe hyperkinesia. J Neurol Sci, 391, 31-39. doi:10.1016/j.jns.2018.05.018

Kulkarni, N., Tang, S., Bhardwaj, R., Bernes, S., & Grebe, T. A. (2016). Progressive Movement Disorder in Brothers Carrying a GNAO1 Mutation Responsive to Deep Brain Stimulation. J Child Neurol, 31(2), 211-214. doi:10.1177/0883073815587945

Kwong, A. K., Tsang, M. H., Fung, J. L., Mak, C. C., Chan, K. L., Rodenburg, R. J. T., . . . Fung, C. W. (2021). Exome sequencing in paediatric patients with movement disorders. Orphanet J Rare Dis, 16(1), 32. doi:10.1186/s13023-021-01688-6

Lecoquierre F. (2018). Caractérisation des variations faux-sens à effet non-haploinsuffisant dans les maladies rares grâce à l’agrégation de données d’exome. Médecine humaine et pathologie.

Ling, W., Huang, D., Yang, F., Yang, Z., Liu, M., Zhu, Q., . . . Chen, X. (2022). Treating GNAO1 mutation-related severe movement disorders with oxcarbazepine: a case report. Transl Pediatr, 11(9), 1577-1587. doi:10.21037/tp-22-297

Liu, P., Meng, L., Normand, E. A., Xia, F., Song, X., Ghazi, A., . . . Yang, Y. (2019). Reanalysis of Clinical Exome Sequencing Data. N Engl J Med, 380(25), 2478-2480. doi:10.1056/NEJMc1812033

Liu, Y., Zhang, Q., Wang, J., Liu, J., Yang, W., Yan, X., . . . Yang, H. (2022). Both subthalamic and pallidal deep brain stimulation are effective for GNAO1-associated dystonia: three case reports and a literature review. Ther Adv Neurol Disord, 15, 17562864221093507. doi:10.1177/17562864221093507

Mahfouz, N. A., Kizhakkedath, P., Ibrahim, A., El Naofal, M., Ramaswamy, S., Harilal, D., . . . Tayoun, A. A. (2020). Utility of clinical exome sequencing in a complex Emirati pediatric cohort. Comput Struct Biotechnol J, 18, 1020-1027. doi:10.1016/j.csbj.2020.04.013

Malaquias, M. J., Fineza, I., Loureiro, L., Cardoso, L., Alonso, I., & Magalhaes, M. (2019). GNAO1 mutation presenting as dyskinetic cerebral palsy. Neurol Sci, 40(10), 2213-2216. doi:10.1007/s10072-019-03964-7

Marce-Grau, A., Dalton, J., Lopez-Pison, J., Garcia-Jimenez, M. C., Monge-Galindo, L., Cuenca-Leon, E., . . . Macaya, A. (2016). GNAO1 encephalopathy: further delineation of a severe neurodevelopmental syndrome affecting females. *Orphanet J Rare Dis, 11*, 38. doi:10.1186/s13023-016-0416-0

Marecos, C., Duarte, S., Alonso, I., Calado, E., & Moreira, A. (2018). [GNAO1: a new gene to consider on early-onset childhood dystonia]. Rev Neurol, 66(9), 321-322.

Matthews, A. M., Blydt-Hansen, I., Al-Jabri, B., Andersen, J., Tarailo-Graovac, M., Price, M., . . . the, C. S. (2019). Atypical cerebral palsy: genomics analysis enables precision medicine. Genet Med, 21(7), 1621-1628. doi:10.1038/s41436-018-0376-y

May, H. J., Fasheun, J. A., Bain, J. M., Baugh, E. H., Bier, L. E., Revah-Politi, A., . . . Carmel, J. B. (2021). Genetic testing in individuals with cerebral palsy. Dev Med Child Neurol, 63(12), 1448-1455. doi:10.1111/dmcn.14948

Menke, L. A., Engelen, M., Alders, M., Odekerken, V. J., Baas, F., & Cobben, J. M. (2016). Recurrent GNAO1 Mutations Associated With Developmental Delay and a Movement Disorder. J Child Neurol, 31(14), 1598-1601. doi:10.1177/0883073816666474

Miyamoto, S., Nakashima, M., Fukumura, S., Kumada, S., & Saitsu, H. (2022). An intronic GNAO1 variant leading to in-frame insertion cause movement disorder controlled by deep brain stimulation. Neurogenetics, 23(2), 129-135. doi:10.1007/s10048-022-00686-5

Monies, D., Abouelhoda, M., Assoum, M., Moghrabi, N., Rafiullah, R., Almontashiri, N., . . . Alkuraya, F. S. (2019). Lessons Learned from Large-Scale, First-Tier Clinical Exome Sequencing in a Highly Consanguineous Population. Am J Hum Genet, 105(4), 879. doi:10.1016/j.ajhg.2019.09.019

Muir, A. M., Myers, C. T., Nguyen, N. T., Saykally, J., Craiu, D., De Jonghe, P., . . . EuroEpinomics-Res Nles working group, S. W. (2019). Genetic heterogeneity in infantile spasms. Epilepsy Res, 156, 106181. doi:10.1016/j.eplepsyres.2019.106181

Nakamura, K., Kodera, H., Akita, T., Shiina, M., Kato, M., Hoshino, H., . . . Saitsu, H. (2013). De Novo mutations in GNAO1, encoding a Galphao subunit of heterotrimeric G proteins, cause epileptic encephalopathy. Am J Hum Genet, 93(3), 496-505. doi:10.1016/j.ajhg.2013.07.014

Nashabat, M., Al Qahtani, X. S., Almakdob, S., Altwaijri, W., Ba-Armah, D. M., Hundallah, K., . . . Alfadhel, M. (2019). The landscape of early infantile epileptic encephalopathy in a consanguineous population. Seizure, 69, 154-172. doi:10.1016/j.seizure.2019.04.018

Novelli, M., Galosi, S., Zorzi, G., Martinelli, S., Capuano, A., Nardecchia, F., . . . Leuzzi, V. (2023). GNAO1-related movement disorder: An update on phenomenology, clinical course, and response to treatments. Parkinsonism & related disorders.

Okumura, A., Maruyama, K., Shibata, M., Kurahashi, H., Ishii, A., Numoto, S., . . . Kojima, S. (2018). A patient with a GNAO1 mutation with decreased spontaneous movements, hypotonia, and dystonic features. Brain Dev, 40(10), 926-930. doi:10.1016/j.braindev.2018.06.005

Pawłowicz, M. (2021). Probable dysfunction of GABA-A receptors in patients with GNAO1-related syndromes. 2021;. eISSN 2391-8306. . Journal of Education, Health and Sport., 11(12), 241-253.

Powis, Z., Towne, M. C., Hagman, K. D. F., Blanco, K., Palmaer, E., Castro, A., . . . Sidiropoulos, C. (2020). Clinical diagnostic exome sequencing in dystonia: Genetic testing challenges for complex conditions. Clin Genet, 97(2), 305-311. doi:10.1111/cge.13657

Retterer, K., Juusola, J., Cho, M. T., Vitazka, P., Millan, F., Gibellini, F., . . . Bale, S. (2016). Clinical application of whole-exome sequencing across clinical indications. Genet Med, 18(7), 696-704. doi:10.1038/gim.2015.148

Rim, J. H., Kim, S. H., Hwang, I. S., Kwon, S. S., Kim, J., Kim, H. W., . . . Kang, H. C. (2018). Efficient strategy for the molecular diagnosis of intractable early-onset epilepsy using targeted gene sequencing. BMC Med Genomics, 11(1), 6. doi:10.1186/s12920-018-0320-7

Rosello, M., Caro-Llopis, A., Orellana, C., Oltra, S., Alemany-Albert, M., Marco-Hernandez, A. V., . . . Tomas, M. (2021). Hidden etiology of cerebral palsy: genetic and clinical heterogeneity and efficient diagnosis by next-generation sequencing. Pediatr Res, 90(2), 284-288. doi:10.1038/s41390-020-01250-3

Saitsu, H., Fukai, R., Ben-Zeev, B., Sakai, Y., Mimaki, M., Okamoto, N., . . . Matsumoto, N. (2016). Phenotypic spectrum of GNAO1 variants: epileptic encephalopathy to involuntary movements with severe developmental delay. Eur J Hum Genet, 24(1), 129-134. doi:10.1038/ejhg.2015.92

Schirinzi, T., Garone, G., Travaglini, L., Vasco, G., Galosi, S., Rios, L., . . . Leuzzi, V. (2019). Phenomenology and clinical course of movement disorder in GNAO1 variants: Results from an analytical review. Parkinsonism Relat Disord, 61, 19-25. doi:10.1016/j.parkreldis.2018.11.019

Schöne-Bake, J. A., B.; Jack, T.; Hartmann, H.;. (2018). GNAO1-Associated Hyperkinetic-Dystonic Movement Disorder and Developmental Delay in a 14-Year-Old Girl. Neuropediatrics, 49, S1-S69.

Schorling, D. C., Dietel, T., Evers, C., Hinderhofer, K., Korinthenberg, R., Ezzo, D., . . . Kirschner, J. (2017). Expanding Phenotype of De Novo Mutations in GNAO1: Four New Cases and Review of Literature. *Neuropediatrics, 48*(5), 371-377. doi:10.1055/s-0037-1603977

Shah, S. (2022). Phenotypic spectrum of GNAO1 mutation 7th International Symposium on Paediatric Movement Disorders.

Slepak, V. Z., Quick, M. W., Aragay, A. M., Davidson, N., Lester, H. A., & Simon, M. I. (1993). Random mutagenesis of G protein alpha subunit G(o)alpha. Mutations altering nucleotide binding. Journal of Biological Chemistry, 268(29), 21889-21894.

Solis, G. P., Kozhanova, T. V., Koval, A., Zhilina, S. S., Mescheryakova, T. I., Abramov, A. A., . . . Katanaev, V. L. (2021). Pediatric Encephalopathy: Clinical, Biochemical and Cellular Insights into the Role of Gln52 of GNAO1 and GNAI1 for the Dominant Disease. Cells, 10(10). doi:10.3390/cells10102749

Takata, A., Nakashima, M., Saitsu, H., Mizuguchi, T., Mitsuhashi, S., Takahashi, Y., . . . Matsumoto, N. (2019). Comprehensive analysis of coding variants highlights genetic complexity in developmental and epileptic encephalopathy. Nat Commun, 10(1), 2506. doi:10.1038/s41467-019-10482-9

Takezawa, Y., Kikuchi, A., Haginoya, K., Niihori, T., Numata-Uematsu, Y., Inui, T., . . . Kure, S. (2018). Genomic analysis identifies masqueraders of full-term cerebral palsy. Ann Clin Transl Neurol, 5(5), 538-551. doi:10.1002/acn3.551

Talvik, I., Moller, R. S., Vaher, M., Vaher, U., Larsen, L. H., Dahl, H. A., . . . Talvik, T. (2015). Clinical Phenotype of De Novo GNAO1 Mutation: Case Report and Review of Literature. Child Neurol Open, 2(2), 2329048X15583717.

Thiel, M., Bamborschke, D., Janzarik, W. G., Assmann, B., Zittel, S., Patzer, S., . . . Koy, A. (2023). Genotype-phenotype correlation and treatment effects in young patients with GNAO1-associated disorders. Journal of Neurology Neurosurgery and Psychiatry. doi:10.1136/jnnp-2022-330261doi:10.1177/2329048X15583717

Turro, E., Astle, W. J., Megy, K., Graf, S., Greene, D., Shamardina, O., . . . Ouwehand, W. H. (2020). Whole-genome sequencing of patients with rare diseases in a national health system. Nature, 583(7814), 96-102. doi:10.1038/s41586-020-2434-2

van der Ven, A. T., Johannsen, J., Kortum, F., Wagner, M., Tsiakas, K., Bierhals, T., . . . Hempel, M. (2021). Prevalence and clinical prediction of mitochondrial disorders in a large neuropediatric cohort. Clin Genet, 100(6), 766-770. doi:10.1111/cge.14061

Waak, M., Mohammad, S. S., Coman, D., Sinclair, K., Copeland, L., Silburn, P., . . . Malone, S. (2018). GNAO1-related movement disorder with life-threatening exacerbations: movement phenomenology and response to DBS. J Neurol Neurosurg Psychiatry, 89(2), 221-222. doi:10.1136/jnnp-2017-315653

Wirth, T., Garone, G., Kurian, M. A., Piton, A., Millan, F., Telegrafi, A., . . . Anheim, M. (2022). Highlighting the Dystonic Phenotype Related to GNAO1. Mov Disord. doi:10.1002/mds.29074

Xiong, J., Peng, J., Duan, H. L., Chen, C., Wang, X. L., Chen, S. M., & Yin, F. (2018). [Recurrent convulsion and pulmonary infection complicated by psychomotor retardation in an infant]. Zhongguo Dang Dai Er Ke Za Zhi, 20(2), 154-157.

Yamamoto, E. A., Berry, M., Harris, W., Shahin, M. N., Wilson, J. L., Safarpour, D., & Raslan, A. M. (2022). Good Response to Deep Brain Stimulation in Two Forms of Inherited Chorea Related to GNAO1 and Neuroacanthocystosis with Illustrative Videos. Mov Disord Clin Pract, 9(3), 401-403. doi:10.1002/mdc3.13383

Yamashita, Y., Ogawa, T., Ogaki, K., Kamo, H., Sukigara, T., Kitahara, E., . . . Hattori, N. (2020). Neuroimaging evaluation and successful treatment by using directional deep brain stimulation and levodopa in a patient with GNAO1-associated movement disorder: A case report. J Neurol Sci, 411, 116710. doi:10.1016/j.jns.2020.116710

Yang, X., Niu, X., Yang, Y., Cheng, M., Zhang, J., Chen, J., . . . Zhang, Y. (2021). Phenotypes of GNAO1 Variants in a Chinese Cohort. Front Neurol, 12, 662162. doi:10.3389/fneur.2021.662162

Yilmaz, S., Turhan, T., Ceylaner, S., Gokben, S., Tekgul, H., & Serdaroglu, G. (2016). Excellent response to deep brain stimulation in a young girl with GNAO1-related progressive choreoathetosis. Childs Nerv Syst, 32(9), 1567-1568. doi:10.1007/s00381-016-3139-6

Yuskaitis, C. J., Ruzhnikov, M. R. Z., Howell, K. B., Allen, I. E., Kapur, K., Dlugos, D. J., . . . Sherr, E. H. (2018). Infantile Spasms of Unknown Cause: Predictors of Outcome and Genotype-Phenotype Correlation. Pediatr Neurol, 87, 48-56. doi:10.1016/j.pediatrneurol.2018.04.012

Zech, M., Jech, R., Boesch, S., Skorvanek, M., Weber, S., Wagner, M., . . . Winkelmann, J. (2020). Monogenic variants in dystonia: an exome-wide sequencing study. Lancet Neurol, 19(11), 908-918. doi:10.1016/S1474-4422(20)30312-4

Zhu, X., Petrovski, S., Xie, P., Ruzzo, E. K., Lu, Y. F., McSweeney, K. M., . . . Goldstein, D. B. (2015). Whole-exome sequencing in undiagnosed genetic diseases: interpreting 119 trios. Genet Med, 17(10), 774-781. doi:10.1038/gim.2014.191

Zou, D., Wang, L., Liao, J., Xiao, H., Duan, J., Zhang, T., . . . Guo, J. (2021). Genome sequencing of 320 Chinese children with epilepsy: a clinical and molecular study. Brain, 144(12), 3623-3634. doi:10.1093/brain/awab233
